# Supplementary material for: Mental health issues and the association of mental health literacy among adolescents in urban Ethiopia
Source: PLoS One. 2024 Oct 24;19(10):e0295545. doi: 10.1371/journal.pone.0295545 (PMC11500858; doi:10.1371/journal.pone.0295545)
Supplement: Attachment — Submitted filename: PONE-D-23-13300_R2.pdf [file pone.0295545.s004.pdf]

PLOS ONE

Mental health issues and the association of mental health literacy among adolescents in urban Ethiopia

--Manuscript Draft--

**Manuscript Number:**	PONE-D-23-13300R2
**Article Type:**	Research Article
**Full Title:**	Mental health issues and the association of mental health literacy among adolescents in urban Ethiopia
**Short Title:**	Mental health issues and the association of mental health literacy among adolescents in urban Ethiopia
**Corresponding Author:**	Hailemariam Mamo Hassen, PhD<br>Dire Dawa University<br>Dire Dawa, --- Select One --- ETHIOPIA
**Keywords:**	Adolescent mental health; mental well-being; mental health literacy; Total strength difficulties Scores; Behavioral and Emotional problems
**Abstract:**	Background<br><br>Epidemiological evidence about the prevalence of adolescent mental health issues and their association with mental health literacy is crucial for sustained mental health promotion strategies. Adolescence is a critical life stage for mental health promotion. However, evidence is not available among Ethiopian school adolescents. Hence, the present study examined the prevalence of adolescents' mental health issues and their correlation with mental health literacy.<br><br>Materials and methods<br><br>A cross-sectional study was conducted among adolescents (grades 5-12) in Dire Dawa city, Eastern Ethiopia using multistage random sampling. Data was collected using the Strength and Difficulty Questionnaire, WHO-5 well-being index, and mental health literacy questionnaire. SPSS version 25 was used for the descriptive, Chi-square, binary logistic regression, and correlation analyses.<br><br>Results<br><br>About 15.9-28.4% of adolescents had reported mental health problems: internalizing problems (14.9-28.4%), emotional problems (10.4-63.9%), and peer relationship problems (17.8-25.8%). These mental health problems were significantly greater among adolescents who had either themselves or their family members used psychoactive substances (p≤0.05). Females from upper elementary (5-8 grade) and lower secondary (9-10) grade levels had a higher prevalence of mental health problems (AOR: 2.60 (0.95-7.10, p<0.05)). The effect of age, parental education, or employment status was insignificant (p>0.05). The prevalence of depression ranged from 18.0-25.5%. Mental health literacy was negatively correlated with total difficulties scores and positively associated with mental well-being scores (p<0.05).<br><br>Conclusion<br><br>The prevalence of adolescents' mental health problems was higher. It implied that promoting mental health literacy could enhance adolescents' positive mental health. Intervention programs should prioritize vulnerable groups and individuals reporting symptoms of mental health difficulties. Future studies should involve qualitative studies and consider effect of other determinants.
**Order of Authors:**	Hailemariam Mamo Hassen, PhD
	Manas Ranjan Behera, DrPH
	Deepanajli Behera, Assistant Professor

Powered by Editorial Manager® and ProduXion Manager® from Aries Systems Corporation

	Ranjit Kumar Dehury, PhD
**Response to Reviewers:**	Response to Reviewers<br>Title: Mental health issues and the association of mental health literacy among adolescents in urban Ethiopia (PONE-D-23-13300)<br>Dear Editor,<br>We thank you for your and the reviewers' constructive feedback and the opportunity to revise and resubmit our manuscript. We carefully considered each point raised by the academic editor and reviewers and revised the manuscript. We appreciate and thank you very much for the feedbacks and comments. We followed PLOS ONE's style requirements.<br>Point raised by the academic editor were regarding whether consent obtained from parents or guardians of the minors, ethics approval letter from two institutions of which one was missing and discrepancy in date difference for secretary and chair person for ethics approval letter obtained from Haramaya University and concern to ensure completeness and correctness of references.<br>Dear Academic editor, regarding consent from parents or guardians of the minors, written informed consent was obtained from parents of adolescents aged under fifteen years had offered their consent and adolescents of fifteen and above years were factored out following the school counsellors and school principals adequate explanation about their level of maturity and decisional capacity ((page 10) with due reference of the guideline International Ethical Guidelines for Health-related Research Involving Humans: Prepared by the Council for International Organizations of Medical Sciences (CIOMS) in collaboration with the World Health Organization (WHO,2016). However, respective school principals as a duly authorized representative had expressed their consent both orally and signed on consent forms for all participants.<br>Regarding the ethics approval letters, ethics approval letter from the two institutions (KIIT University and Haramaya University) are merged and attached in this revised submission. The discrepancy of the date of signature from the Chairperson (dated 11/02/2018) and Secretary (dated 11/02/2020) on same document was confirmed as typo while the typist was writing and the letter issued unnoticed. We understand that it requires a correction. However, our application for correction from the Institutional review board of Haramaya University failed because the Institutional review board has restructured and the Signers were changed by other individuals. We sincerely request your esteemed editor to understand that this discrepancy is typo while the typist was writing and we kindly request you recognize this issue.<br>We reviewed the reference lists to ensure it is complete and correct.<br>Dear editor, we appreciate the comments raised by the reviewers and we accommodate each comment one by one in the manuscript indicated by track change. Three separate papers are submitted to the system: one has a marked-up copy of the manuscript that highlights changes with track changes, an unmarked version of the revised paper without tracked changes, and a rebuttal letter for each point raised by the academic Editor and reviewers. We are confident that the updated version is appropriate for publishing and anticipate hearing from you soon.<br>With regards,<br>Hailemariam Mamo Hassen (PhD), (First and corresponding author)<br>On behalf of the authors.<br><br>Point-by-point response for Reviewers' comments<br>Dear reviewers, we are grateful for the feedback and constructive comments helpful for improving the manuscript quality.<br>Reviewer #2 Comments<br>Thank you very much, dear reviewers.<br>Reviewer #2 commented that we authors have addressed all the comments and suggestions stating that mental health literacy is an emerging issue which demand more attention for overall health and well-being.<br>Authors' response Thank you very much:<br>Reviewer #3 Comments<br>Author's response: Thank you. We appreciate your constructive comments and agree on the comments and addressed accordingly. .<br>Reviewer #3 comment: Would you please review your claims on the discussion section regarding government actions taken at school level with regard to mental health promotion and prevention as there are no promising activities? These are actually policy promises than actual practices.<br>Authors' response: Dear Reviewer, thank you so much for your essential concern. We

	agree that the government actions taken at school level with regard to mental health promotion and prevention are policy promises than actual practices. Accordingly, we discussed these points. Despite the delayed initiation of the policy and lack of established systems and practices, there has been growing recognition of the importance of adolescent mental health promotion. The Ethiopian government has shown commitment to promoting mental health and addressing mental health issues in schools, for instance, by employing trained school counselors and establishing clubs in schools in secondary schools. (stated on page 25)<br>Reviewer #3 comment: I think you better consider including the predictor variables in the method part.<br>Author's response: Thank you very much, dear reviewer. We included the predictor variables briefly in the method part (page 8). “The predictor variables mainly focusing on age, grade level, self or any family member experience with psychoactive substance use, parents practicing corporal punishment, perceived worry about family problems, parents education, and job status were collected by using questionnaires.”<br>Reviewer #3 comment: Your sampling strategy should discuss the clusters selected all the stages precisely.<br>Author's response: Thank you. We mentioned the sampling strategy briefly in the method part (page 8). “A combination of multistage (schools, classrooms, then individual students) and systematic and random (using the list of the students in fixed intervals of their roll numbers) was used to select study participants from public and private schools.”<br>Reviewer #3 comment: The statement on page 13 that begins with "These mental health problems were..." does not provide a clear comparison.<br>Author's response: Once again, we appreciate your concern. We have compared and indicated in the figure 1 in the revised manuscript, page 14. We stated that “These mental health problems were more prevalent for female and male adolescents compared to other age groups in extent of percentage as explicitly visualized in Figure 1 across the three age groups. Prevalence of depression ranged from 18.0%-25.5%, reportedly higher for female adolescents aged 14-16 years (page 14).<br><br>Reviewer #3 comment: Would you please mention any bases for your age categorization and its subsequent implication for mental health promotion and prevention?<br>Author's response: Thank you very much, dear reviewer. We already mentioned the bases for your age categorization and its subsequent implication on page 11 of the last 4 lines #6-9; “The age range for adolescents and grouping was based on UNICEF adolescents' age categorization as early, middle, and late adolescence age groups, which is helpful to target mental health promotion and prevention efforts during these stages to address their respective mental health demands.”
**Additional Information:**	
**Question**	**Response**
**Financial Disclosure**<br><br>Enter a financial disclosure statement that describes the sources of funding for the work included in this submission. Review the submission guidelines for detailed requirements. View published research articles from *PLOS ONE* for specific examples.<br><br>This statement is required for submission and **will appear in the published article** if the submission is accepted. Please make sure it is accurate.	The authors received no specific funding for this work.

**Unfunded studies**<br>Enter: *The author(s) received no specific funding for this work.*<br><br>**Funded studies**<br>Enter a statement with the following details:<br>• Initials of the authors who received each award<br>• Grant numbers awarded to each author<br>• The full name of each funder<br>• URL of each funder website<br>• Did the sponsors or funders play any role in the study design, data collection and analysis, decision to publish, or preparation of the manuscript?<br>• **NO** - Include this sentence at the end of your statement: *The funders had no role in study design, data collection and analysis, decision to publish, or preparation of the manuscript.*<br>• **YES** - Specify the role(s) played.<br><br>* typeset	
**Competing Interests**<br><br>Use the instructions below to enter a competing interest statement for this submission. On behalf of all authors, disclose any competing interests that could be perceived to bias this work—acknowledging all financial support and any other relevant financial or non-financial competing interests.<br><br>This statement is required for submission and **will appear in the published article** if the submission is accepted. Please make sure it is accurate and that any funding sources listed in your Funding Information later in the submission form are also declared in your Financial Disclosure statement.<br><br>View published research articles from *PLOS ONE* for specific examples.	The authors have declared that no competing interests exist.

**NO authors have competing interests**<br><br>Enter: *The authors have declared that no competing interests exist.*<br><br>**Authors with competing interests**<br><br>Enter competing interest details beginning with this statement:<br><br>*I have read the journal's policy and the authors of this manuscript have the following competing interests: [insert competing interests here]*<br><br>* typeset	
**Ethics Statement**<br><br>Enter an ethics statement for this submission. This statement is required if the study involved:<br><br>• Human participants<br>• Human specimens or tissue<br>• Vertebrate animals or cephalopods<br>• Vertebrate embryos or tissues<br>• Field research<br><br>Write "N/A" if the submission does not require an ethics statement.<br><br>General guidance is provided below. Consult the submission guidelines for detailed instructions. **Make sure that all information entered here is included in the Methods section of the manuscript.**	Ethical approval was obtained from KIIT University (Reference: KIIT/KIMS /IEC/63/2019) and Haramaya University (Reference: 00. HMS/10.0/3763/2020. Written informed consent was obtained from all participants. As a duly authorized representative, written informed consent was obtained from school principals.

Powered by Editorial Manager® and ProduXion Manager® from Aries Systems Corporation

**Format for specific study types**<br><br>**Human Subject Research (involving human participants and/or tissue)**<br>• Give the name of the institutional review board or ethics committee that approved the study<br>• Include the approval number and/or a statement indicating approval of this research<br>• Indicate the form of consent obtained (written/oral) or the reason that consent was not obtained (e.g. the data were analyzed anonymously)<br><br>**Animal Research (involving vertebrate animals, embryos or tissues)**<br>• Provide the name of the Institutional Animal Care and Use Committee (IACUC) or other relevant ethics board that reviewed the study protocol, and indicate whether they approved this research or granted a formal waiver of ethical approval<br>• Include an approval number if one was obtained<br>• If the study involved *non-human primates*, add *additional details* about animal welfare and steps taken to ameliorate suffering<br>• If anesthesia, euthanasia, or any kind of animal sacrifice is part of the study, include briefly which substances and/or methods were applied<br><br>**Field Research**<br><br>Include the following details if this study involves the collection of plant, animal, or other materials from a natural setting:<br>• Field permit number<br>• Name of the institution or relevant body that granted permission	
**Data Availability**<br><br>Authors are required to make all data underlying the findings described fully available, without restriction, and from the time of publication. PLOS allows rare exceptions to address legal and ethical concerns. See the PLOS Data Policy and FAQ for detailed information.	Yes - all data are fully available without restriction

Powered by Editorial Manager® and ProduXion Manager® from Aries Systems Corporation

A Data Availability Statement describing where the data can be found is required at submission. Your answers to this question constitute the Data Availability Statement and **will be published in the article**, if accepted.<br><br>**Important:** Stating 'data available on request from the author' is not sufficient. If your data are only available upon request, select 'No' for the first question and explain your exceptional situation in the text box.<br><br>Do the authors confirm that all data underlying the findings described in their manuscript are fully available without restriction?	
**Describe where the data may be found in full sentences. If you are copying our sample text, replace any instances of XXX with the appropriate details.**<br><br>• If the data are **held or will be held in a public repository**, include URLs, accession numbers or DOIs. If this information will only be available after acceptance, indicate this by ticking the box below. For example: *All XXX files are available from the XXX database (accession number(s) XXX, XXX.*).<br>• If the data are all contained **within the manuscript and/or Supporting Information files**, enter the following: *All relevant data are within the manuscript and its Supporting Information files.*<br>• If neither of these applies but you are able to provide **details of access elsewhere**, with or without limitations, please do so. For example:<br><br>*Data cannot be shared publicly because of [XXX]. Data are available from the XXX Institutional Data Access / Ethics Committee (contact via XXX) for researchers who meet the criteria for access to confidential data.*<br><br>*The data underlying the results presented in the study are available from (include the name of the third party*	All relevant data are within the paper and its Supporting Information files.

and contact information or URL).<br>• This text is appropriate if the data are owned by a third party and authors do not have permission to share the data.<br><br>* typeset	
Additional data availability information:	Tick here if your circumstances are not covered by the questions above and you need the journal's help to make your data available.

Powered by Editorial Manager® and ProduXion Manager® from Aries Systems Corporation

Manuscript

Click here to access/download;Manuscript;Revised manuscript without tracked changes.docx

Original Research

Mental health issues and the association of mental health literacy among adolescents in urban Ethiopia

Hailemariam Mamo Hassen[1], Manas Ranjan Behera[2*], Deepanajli Behera[2], Ranjit Kumar Dehury[3]

[1]Department of Biological Sciences, Dire Dawa University, Dire Dawa, Ethiopia

[2] School of Public Health, KIIT Deemed to be University, Bhubaneswar, India

[3]School of Management Studies, University of Hyderabad, Telangana, India

* manas.beherafph@kiit.ac.in

Abstract

Background

Epidemiological evidence about the prevalence of adolescent mental health issues and their association with mental health literacy is crucial for sustained mental health promotion strategies. Adolescence is a critical life stage for mental health promotion. However, evidence is not available among Ethiopian school adolescents. Hence, the present study examined the prevalence of adolescents' mental health issues and their correlation with mental health literacy.

Formatted: Justified

Materials and methods

A cross-sectional study was conducted among adolescents (grades 5-12) in Dire Dawa city, Eastern Ethiopia using multistage random sampling. Data was collected using the Strength and Difficulty Questionnaire, WHO-5 well-being index, and mental health literacy questionnaire**.** SPSS version 25 was used for the descriptive, Chi-square, binary logistic regression, and correlation analyses.

Results

About 15.9-28.4% of adolescents had reported mental health problems: internalizing problems (14.9-28.4%), emotional problems (10.4-63.9%), and peer relationship problems (17.8-25.8%). These mental health problems were significantly greater among adolescents who had either themselves or their family members used psychoactive substances ($p \leq 0.05$). Females from upper elementary (5-8 grade) and lower secondary (9-10) grade levels had a higher prevalence of mental health problems (AOR: 2.60 (0.95-7.10, $p<0.05$)). The effect of age, parental education, or employment status was insignificant ($p>0.05$). The prevalence of depression ranged from 18.0-25.5%. Mental health literacy was negatively correlated with total difficulties scores and positively associated with mental well-being scores ($p<0.05$).

Conclusion

The prevalence of adolescents' mental health problems was higher. It implied that promoting mental health literacy could enhance adolescents' positive mental health. Intervention programs should prioritize vulnerable groups and individuals reporting symptoms of mental health difficulties. Future studies should involve qualitative studies and consider effect of other determinants.

Formatted: Justified

1. Introduction

Globally, mental health problems disproportionately affect adolescents in low-income countries and they remain the leading causes of disease burden among children and young people.[1] Knowing about these mental health conditions, their symptoms, and treatments termed as mental health literacy is essential for everyone but is crucial for adolescents.[2] Mental health literacy is defined as understanding and applying information about mental health. [2] In this regard, much has been known about the mental health literacy of adolescents from high-income countries.[3] However, it is still a relatively new concept in Africa.[2] Additionally, studies revealed that access to mental health information or services in Africa is lower due to poverty, lack of education, and cultural stigma around mental illness.[1] Nevertheless, limited evidence in Africa, including Ethiopia, has indicated a growing awareness about the importance of mental health literacy. Despite the challenges associated with poverty, cultural stigma, lack of access to health facilities, and lower education coverage, countries are working to improve mental health literacy.[2]

The Ethiopian Ministry of Health has developed a mental health policy focusing on primary mental healthcare and promoting positive mental health, including a mental health awareness campaign[4]. The present study area is Dire Dawa, one of Ethiopian provinces known for its ethnic and cultural language diversity. A recent study revealed that adolescent mental health literacy from Dire Dawa city was lower, consistent with similar studies reports and showing variability across socio-demographic factors. [5]

Epidemiological evidence about the prevalence of mental health problems and their association with mental health literacy is crucial for sustained mental health promotion strategies, as adolescence is a critical life stage for mental health promotion.[6–10] However, such evidence is rarely available among Ethiopian school adolescents. Adolescent mental

Formatted: Justified

health has piqued the interest of public health professionals due to the unique nature of adolescence.[11] Therefore, adolescence remains a critical life stage for mental health promotion.[11] A popular saying goes, “there is no health without mental health.” [12] With multifaceted burdens, mental health is integrally linked to physical health and well-being.[13] The typical adolescent mental health problems include but are not limited to: substance use/abuse or disorders, depression/mood disorders, anxiety, schizophrenia, stress/neurosis, behavioral disorders, postpartum psychosis, and posttraumatic stress disorder (PTSD). [14,15] Several studies showed that these mental health problems in adulthood occur during adolescence. [14,15]

For multiple reasons, adolescents are among the most important groups for mental health studies. For example, adolescence is key to physical, emotional, and cognitive progress.[11,16,17] Fundamentally, mental, physical, and emotional developmental processes evolve during adolescence. [18] Also public health perspectives emphasizing mental health information during early childhood,[18] would help in later life in managing and preventing mental health problems.[18]

Mental health literacy is essential to preventing mental illnesses, consisting of knowledge about mental health problems and recognizing illnesses with their symptoms.[1,3] Studying the mental health status and its association with the mental health literacy of adolescents is paramount because it helps understand these variables across varied cultural settings.[1] The peak of the onset of mental disorders mainly occurs in the adolescent period and needs urgent attention. [19] In low-resource countries like Ethiopia, risk factors such as cigarette smoking, alcohol consumptions, early co-habitation, unhealthy lifestyles etc are higher among adolescents.[20,21] leading to various mental health problems.[11,16,17] Hence, epidemiological methods like cross-sectional studies provides empirical evidence about the mental health status and factors that contribute to mental health problems.[22] Early understanding of these

Formatted: Justified

evidence contribute for making informed and effective interventions to improve adolescents' mental health. [23]

As a result, there is an increasing demand for adolescents' mental health and well-being outcome measures to inform the public and policymakers regarding individual or service-level health promotion and therapeutic practices. In this regard, the strength difficulties questionnaire (SDQ) and the mental well-being index (WHO-5) have been essential to meet the criteria of valid and reliable measures.

The Strengths and Difficulties Questionnaire (SDQ) and the WHO-5 Well-Being Index are commonly used to assess adolescents' mental health.[24–34] The SDQ is a 25-item self-report questionnaire that assesses five mental health domains: conduct problems, emotional symptoms, peer problems, hyperactivity-inattention, and pro-social behavior. [24,25,27–29] The WHO-5 is a 5-item self-report questionnaire that assesses overall well-being. [26,30–34] Both instruments are good choices for assessing adolescent mental health in low-resource settings because they are brief, relatively inexpensive, and easy to administer, and shown to be reliable and valid in adolescents from low-resource settings have been validated in a variety of settings and translated into multiple languages for low-resource settings including in Ethiopia[35,36] Therefore, the SDQ and the WHO-5 are good choices for assessing adolescent mental health in low-resource settings. In this article, the concept of "normalcy" and "mentally sick" as two separate spheres was considered by taking the categorical approach than the dimensional approach of measuring mental health status. [37,38]

The assessment of mental health issues using the familiar Diagnostic Standard Manual – 5 (DSM-5) and International Classification of Disease (ICD-11) analysis has been published in previous studies.[37,38] Such an approach is marked by clustering disorders, namely, externalizing and internalizing problems. Instruments with high validity are routinely used to

Formatted: Justified

measure the internalizing groups (i.e., depressive, somatic, and anxiety symptoms) and the externalizing groups (substance use, disruptive, and conduct symptoms). Furthermore, the well-being state was examined using the WHO-5 index, with lower scores indicating depression.

The daily suffering of people with mental illness affects family members, mainly children, and adolescent populations.[39] Globally, about 25% of the total population and 10-20% of children and adolescents have mental disorders, of which half of all these mental health problems begin by the age of fourteen and three-fourths by the age of early twenties.[40,41] A worldwide-pooled prevalence meta-analysis on mental disorders from all regions of the world addressing 27 countries showed a prevalence of 13.4% ranging from 11.3 to 15.9 (CI: 95%), which indicates mental disorders is affecting a large proportion of the child and adolescent population.[42] According to studies that used the strengths and difficulties questionnaire (SDQ), around 10.5% (5.8-15%) of adolescents in developing countries had mental health concerns.[28]

The prevalence of mental health problems among adolescents in Africa including Ethiopia, was reportedly higher.[43–47] For instance, a meta-analysis study on adolescents in sub-Saharan Africa, including Ethiopia (N=9713), showed the proportion of adolescents having psychopathology was 14.3% (13.6%-15.0%), and the prevalence of psychological disorders was 19.8% (18.8%-20.7%).[45]

These mental health problems are exacerbated by modifiable factors such as low mental health service seeking, lower mental health service use, stigma, discrimination, and common human rights abuses. These factors are linked to immediate and intermediate mental health outcomes such as health literacy and behaviour. Mental health literacy is a changeable factor linked to improved psychological outcomes.[6–10] It refers to how well a person understands

Formatted: Justified

mental diseases and the associated factors.[6–10] Mental health literacy includes an individual's potential to identify mental distress.[9,48,49] Some qualitative descriptions revealed that mental health difficulties and mental well-being is associated with mental health literacy, despite the lack of quantitative evidence of the correlation between mental health literacy, mental health status, and mental well-being.[30]

There has been a scarcity of available epidemiological evidence about mental health conditions and literacy among Ethiopian school adolescents. There was also a lack of evidence about school adolescents' mental health status and the effect of socio-demographic characteristics in the existing literature. Therefore, the objectives of the study were:

- To assess the prevalence of mental health issues among urban adolescents in Ethiopia
- To evaluate the relationship between mental health literacy and mental health issues and
- To examine the impact of socio-demographic characteristics on mental health issues

2. Materials and methods

2.1. Study design and sampling

The present research was a cross-sectional study among public and private school adolescents in grades 5 through 12 from 11 to 19 years in Dire Dawa, Ethiopia. A multistage random sampling method was used to choose the respondents. The prevalence of self-reported mental health difficulty was obtained from the pilot study of the same population (p=0.378) [51], and the margin of sampling error (d=0.04) was taken to calculate the sample size.

$$n \geq \frac{(Za/2)^2 \text{ x } P(1-P)}{d^2} = \frac{1.96^2 \text{ x } 0.378(1-0.378)}{0.04^2} = 559.673 \approx 560$$

Formatted: Justified

Data was collected from June 2020 to July 2020 using the mental health literacy questionnaire (MHLQ), the Strength and Difficulty Questionnaire (SDQ), and the WHO-5 well-being index after cross-cultural validation in the study settings. Maximum sample size estimation (n= 924) was approached after taking design effect (d=1.5) and a 10% non-response. About 751 participants filled out the questionnaires out of the 924 potential participants.

2.2. Data collection tools

Data collection tools were the Strengths and Difficulties Questionnaire (SDQ), mental health literacy questionnaire (MHLQ), and mental well-being index (WHO-5). The Strengths and Difficulties Questionnaire (SDQ) is a valid, rapid measure for emotional and behavioral problems worldwide.[24,25,27–29] SDQ is a 25-item, 3-point Likert scale (0=not true, 1=somewhat true, and 2=certainly true). It measures subscale difficulty problems that are emotional(SDQ3,8,13,16&24), conduct (SDQ5,7,12,18&22), hyperactivity/inattention(SDQ 2,10,15,21&25), peer(SDQ6,11,14,19&23) and pro-social behaviour (SDQ 1,4,9,17&20) problems.[52] The total difficulties score combines conduct, hyperactivity, emotional, and peer relationship behaviour problems. The externalizing problem manifests emotional and peer behavioural relationship problems. Meanwhile, the internalizing problem manifests the conduct and hyperactivity/inattention problems.

Adding up the first four subscales leads to total difficulty scores (the higher the total difficulties score, the more significant the mental health difficulty).[52] The fifth sub-scale of the SDQ reflects pro-social behaviour (the higher the score, the better the pro-social behaviour).[52] SDQ is widely used in resource-poor countries to measure children and adolescents' mental and emotional problems. The baseline for cut-off scores definition of total and subscales strength and difficulty scores were from the United Kingdom population

Formatted: Justified

norms defined during the instrument development. [27,53] The self-administered SDQ is a validated and widely used measurement tool for determining mental health problems in children and adolescents.[24,25,27–29] It has already been translated into over 60 languages, including Ethiopian Amharic.[24,25,27–29] It has reportedly been an effective tool for assessing children's mental health in a recent scoping review in Africa. [35]

Mental health literacy was measured using the mental health literacy questionnaire (MHLQ). This MHLQ tool is freely available and validated for adults, [54] and adolescents [7,55–63] living in low-income countries to measure health literacy scores. In the context of the current investigation, its reliability was evaluated using Cronbach's alpha and found to be 0.834. MHLQ has 33 items with five Likert scale ratings *(1=strongly disagree, 2=slightly disagree, 3=neither agree nor disagree, 4=slightly agree, 5=strongly agree).* The MHLQ consists of 33 questions that measure factors like recognition (10 questions), knowledge (8 questions), attitudes (8 questions), and beliefs (7 questions) of the adolescents on mental health status. [6–10] The range of scores for the mental health literacy tool is 33 to 165. A higher score implies a better level of mental health literacy.

The mental well-being index (WHO-5) is an overarching expression of the quality of the various domains in the life of adolescents subjective to their mental and psychological well-being. Mental well-being was assessed using the WHO's well-being index, which consists of 5 items (WHO-5). [26,30–34] It is a Likert-type scale with a five-point scale (5=all of the time, 4=most of the time, 3=more than half the time, 2=less than half the time, 1= some of the time, 0=at no time).

2.3. Ethical approval

Ethical approval was obtained from KIIT University (Reference: KIIT/KIMS /IEC/63/2019) and Haramaya University (Reference: 00. HMS/10.0/3763/2020. Written informed consent

Formatted: Justified

was obtained from all participants. As a duly authorized representative, written informed consent was obtained from school principals.

2.4. Statistical analysis

SPSS version 25 was used to carry out the statistical analysis. Descriptive analysis was done to present the socio-demographic characteristics, cut-off score determination, and prevalence of mental health problems. Chi-square test and binary logistic regression analysis were used to estimate the difference and compare the mental health problems prevalence between males and females across some socio-demographic characteristics. Correlation analysis was performed to assess the associations between mental health literacy, strength difficulties questionnaire scale, and subjective mental well-being. Estimates used a confidence interval of 95 % ($p \leq 0.05$).

The cut-off score for mental health problems(cases) was defined using the original 3 band categorizations (cut-off scores at 80th & 90th).[64–66] Scores of every item in the scale are added from 0–10. The added scores (range of 0–40) were calculated by adding scores on the emotion components, conduct problems, hyperactivity, inattention, and peer problem scales.[27–29] The cut-off scores was defined by percentile into the highest first 10% (abnormal), the next 10% (borderline), and the remaining 80% (normal). The cut-off for mental health problems (caseness) was the highest at first 10 %.

3. Results

3.1. The socio-demographic characteristics of study participants

About 751 participants comprising 50.1% males and 49.9% females, completed the questionnaires, and statistical analysis was performed for the 731 participants after removing

Formatted: Justified

2.66% of respondents from the analysis for significant missing data. It did not affect the sample power because a greater number of participants were participated compared to the calculated minimum sample size (n=565). The mean age with standard deviation of male and female respondents was 16.27 ± 2.19 and 15.95 ± 2.02, respectively.

3.2. Strength difficulty scores and prevalence of mental health status

The original 3 bands (cut-off scores at 80th & 90th) and the newer 4 band (cut-off scores at 80th, 90th & 95th) categorization were reported just for comparison as reported in **Table 1**. The original 3 band was used to determine and estimate adolescents' mental health issues. The mental health problems expressed with the total difficulties and in subscales, in general, were higher for female adolescents than male adolescents. The prevalence of mental health problems (cases) was presented across gender and age categories.

Table 1: The cut of scores for baseline reference ranges (caseness) for both the original 3 band and the newer 4 band categorizations

Self-completed SDQ	Original 3 band categories(cut-off scores at 80th & 90th)						Newer 4 band categorisations (cut-off scores at 80th, 90th & 95th)							
	Normal		Borderline		Abnormal		Close to average		Slightly raised slightly lowered		High (/low)		Very high (/very low	
	Baseline	Present stud	Baseline	Present stud	Baseline	Present	Baseline	Present	Baseline	Present	Baseline	Present	Baseline	Present stud

Formatted: Justified

	norm	y range	norm	y range	norm	study range	norm	study range	norm	study range	norm	study range	norm	y range
Total difficulties score	0-15	0-14	16-19	15-17	20-40	18-40	0-14	0-14	15-17	15-17	18-19	18-20	20-40	20-40
Emotional problems score	0-5	0-5	6	6	7-10	7-10	0-4	0-5	5	6	6	7	7-10	8-10
Conduct problems score	0-3	0-3	4	4	5-10	5-10	0-3	0-3	4	4	5	5	6-10	6-10
Hyperactivity score	0-5	0-4	6	5	7-10	6-10	0-5	0-4	6	5	7	6	8-10	7-9
Peer problems score	0-3	0-4	4-5	5	6-10	6-10	0-2	0-4	3	5	4	6	5-10	7-9
Pro-social score	6-10	6-10	5	5	0-4	0-4	7-10	7-10	6	6	5	5	0-4	0-4
Externalising	0-5	0-5	6-10	6-7	11-20	8-20	0-5	0-5	6-10	6-7	11-12	8	13-20	9-20

Formatted: Justified

score														
Internalising score	0-4	0-8	5-8	9-10	9-20	11-20	0-4	0-8	5-8	9-10	9-10	11-12	11-20	13-20

As indicated in **Fig 1**, the prevalence of mental health total difficulties (15.9-25.5%), internalising (14.9-28.4%), emotional (10.4-23.9%), and peer relationship (17.8-25.8%) problems were higher compared to reports of previous studies. These mental health problems were more prevalent for female and male adolescents. Prevalence of depression ranged from 18.0%-25.5%, reportedly higher for female adolescents aged 14-16 years

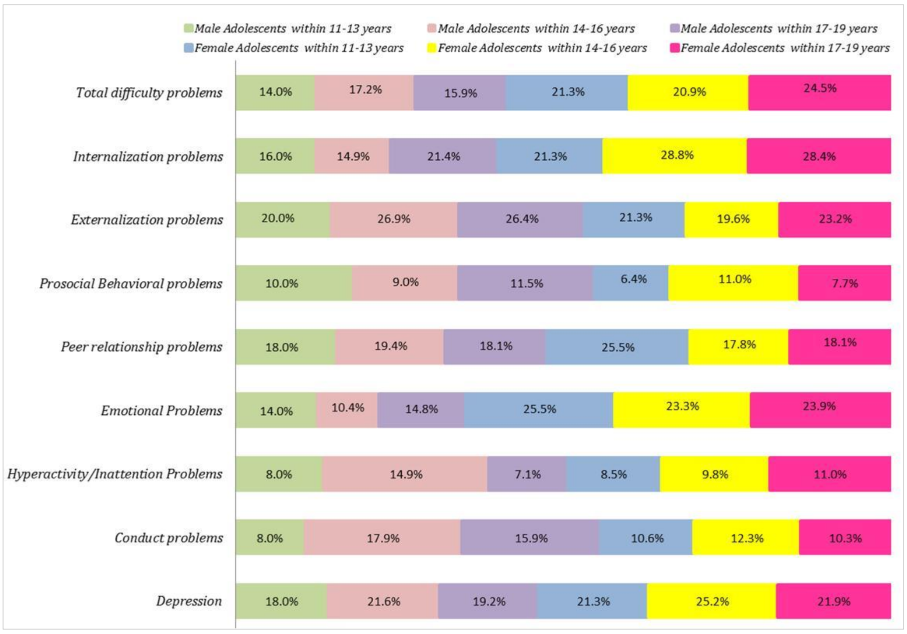

Figure 1: Prevalence of mental health problems among school adolescents in Dire Dawa, Ethiopia.

Formatted: Justified

3.3. Association of mental health status and socio-demographic factors

Statistical difference in prevalence was examined for each banding of subscale using the Chi-square test. The proportion of abnormal females with total difficulties and emotional and internalizing problems was significantly higher ($p<0.05$) than males. The prevalence of conduct and externalizing problems was highly associated with being male. The association of socio-demographic characteristics with mental health status was analyzed using binary logistic regression as reported in **Table 2**.

Table 2: Chi-square test of mental health problems prevalence as per the original 3 band categorization (cut-off scores at 80th & 90th) between males and females

Subscales and bandings	Males (n=366)		Females (n=365)		df	X^2	p
	n	%	n	%			
Total difficulties					2	6.913[a]	0.042
Normal(0-14)	307	83.9%	283	77.5%			
Borderline(15-17)	29	7.9%	48	13.2%			
Abnormal(18-40)	30	8.2%	34	9.3%			
Emotional problems score					2	14.442[a]	0.001
Normal0-5)	318	86.9%	278	76.2%			
Borderline=6	23	6.3%	36	9.9%			
Abnormal(7-10)	25	6.8%	51	14.0%			
Conduct problems							

Formatted: Justified

Normal(0-3)	309	84.4%	324	88.8%	2	3.674[a]	0.159
Borderline=4	30	8.2%	18	4.9%			
Abnormal(5-10)	25.0	7.4%	25.0	6.3%			
Hyperactivity score					2	0.488[a]	0.783
Normal (0-4)	329	89.9%	328	89.9%			
Borderline=5	21	5.7%	18	4.9%			
Abnormal(6-10)	16	4.4%	19	5.2%			
Peer problems score					2	0.930[a]	0.628
Normal(0-4)	298	81.4%	296	81.1%			
Borderline=5	41	11.2%	36	9.9%			
Abnormal(6-10)	27	7.4%	33	9.0%			
Internalising score					2	9.332[a]	0.009
Normal(0-8)	299	81.7%	264	72.3%			
Borderline(9-10)	36	9.8%	50	13.7%			
Abnormal(11-20)	31	8.5%	51	14.0%			
Externalising score					2	1.896[a]	0.387
Normal(0-5)	272	74.3%	287	78.6%			
Borderline(6-7)	50	13.7%	41	11.2%			
Abnormal(8-20)	44	12.0%	37	10.1%			
Pro-social score					2	0.549[a]	0.760
Normal(6-10)	328	89.6%	332	91.0%			
Borderline=5	18	4.9%	14	3.8%			
Abnormal(0-4)	20	5.5%	19	5.2%			

Formatted: Justified

A binary logistic regression model analysis showed that the odds of mental health problems were significantly associated with some socio-demographic characteristics ($p<0.05$); odds were different across these characteristics as reported in **Table 3**. The prevalence of mental health problems was significantly higher for females in upper elementary (AOR: 2.60 (0.95-7.10)) and lower secondary levels (AOR: 2.73 (1.19-6.29)) compared to upper secondary grade level ($p<0.05$); but it was not significantly different for males. It was twice higher among adolescents with a self and family members experiencing psychoactive substance use ($p<0.05$). Prevalence difference across age, maternal and paternal education, and employment status was insignificant ($p>0.05$).

Table 3: Association of mental health problems and socio-demographic determinants analyzed with binary logistic regression

		Prevalence of mental health problem %			
Predictors		Male		Female	
	Categories	UAOR(95% CI)	AOR(95% CI)	UAOR(95% CI)	AOR(95% CI)
	11-19	-	-		-
Age group(yrs)	11-13	1	1	1	1
	14-16	1.27(0.51-3.18)	0.959(0.25-3.7)	0.98 (0.44-2.16)	0.65 (0.20-2.06)
	17-19	1.16(0.48-2.84)	0.643(0.06-6.28)	1.20 (0.55-2.64)	0.53 (0.08-3.38)

Formatted: Justified

Grade level	Upper elementary (5-8)	1.08(0.51-2.31)	1.44(0.50-4.15)	1.57 (0.70-3.50)	2.60 (0.95-7.10)
	Lower Secondary (9-10)	1.02(0.48-2.18)	1.11(0.50-2.46)	2.22 (1.00-4.92)*	2.73 (1.19-6.29)*
	Upper secondary (11-12)	1	1	1	1
Experience on psychoactive substances use					
Self experience	No	1	1	1	1
	Yes	0.45(0.22-0.93*	2.20(1.07-4.52)*	2.17 (0.95-4.95)	2.16 (0.95-4.92)
Any family experience	No	1	1	1	1
	Yes	2.01(1.01-4.00)*	2.04(1.01-4.09)*	1.07(0.63-1.84)	1.06 (0.62-1.81)
Parents practicing corporal punishment	No	1	1	1	1
	Yes	1.24 (0.69-2.24)	1.24 (0.69-2.23)	1.43 (0.82-2.51)	1.33 (0.75-2.36)
Adolescents report perceived	No	1	1	1	1
	Yes	1.186(0.64-2.21)	1.17(0.62-2.20)	1.24 (0.73-2.12)	1.20 (0.70-

Formatted: Justified

worry about family problem					2.06)
Mother education level	Non-educated	1	1	1	1
	Elementary	1.31(0.50-3.43)	1.30 (0.49-3.41)	1.99 (0.57-6.96)	0.73 (0.29-1.87)
	Secondary	1.30(0.66-2.57)	1.30 (0.66-2.58)	1.50 (0.34-6.70)	1.37 (0.78-2.42)
	College or above	0.63(0.18-2.20)	0.64 (0.18-2.24)	2.71 (0.74-9.93)	0.52 (0.15-1.81)
Father education level	Non-educated	1	1	1	1
	Elementary	1.51 (0.47-4.82)	1.48 (0.46-4.74	1.03 (0.21-5.14)	1.00 (0.20-4.99)
	Secondary	1.60 (0.83-3.09)	1.61 (0.83-3.11	1.04 (0.58-1.84)	1.07 (0.60-1.90)
	college or above	1.42 (0.64-3.16)	1.45 (0.65-3.23	1.23 (0.61-2.46)	1.20 (0.60-2.41)
Mother job	House wife/Unemployed	1	1	1	1

Formatted: Justified

	Work in private	0.79 (0.41-1.51)	0.79 (0.41-1.51)	1.40 (0.77-2.53)	1.40 (0.77-2.54)
	Employed	0.44 (0.16-1.22)	0.44 (0.16-1.21)	0.55 (0.22-1.42)	0.55 (0.21-1.44)
Father job	Unemployed/Jobless	1	1	1	1
	Work in private	1.24 (0.66-2.32)	1.24 (0.66-2.33)	0.75(0.43-1.33)	0.77(0.44-1.36)
	Employed	2.56 (0.75-8.74)	2.52 (0.73-8.70)	ns	ns

** Significant at p<0.01 * Significant at p<0.05

However, the prevalence of mental health problems was not significantly different for males by education level. Prevalence difference was insignificant across the three age groups (11-13, 14-16, and 17-19 years), maternal and paternal education level, and types of jobs or level of employment (p>0.05). The prevalence of mental health problems was twice higher among psychoactive substance users (p<0.05) compared with non-users. Differences in prevalence existed but were insignificant across these three age groups, maternal and paternal education level, and types of jobs or level of employment (p>0.05).

Formatted: Justified

3.4. Correlation of mental health literacy with total difficulty scores and well-being index

The bivariate correlations showed that the mental health literacy score was negatively associated with the total and subscale strength difficulties scores (conduct problems, emotional problems, hyperactivity-inattention, and peer problems) as it is shown in **Table 4**.

Table 4: The correlations between mean mental health literacy score, strength difficulties questionnaire scale, and mental wellbeing index

Variables		Correlation coefficient (r)) with 95%CI								
		1	2	3	4	5	6	7	8	9
1.	SDQ Total difficulties problems score	1	-.319** (-.388-(-.250))	-.124** (-.198-(-.052))	.652** (.607-.694)	.700** (.656-.740)	.749** (.714-.783)	617** (.570-.659)	.825** (.798-.848)	.863** (.842-.882)
2.	Mental wellbeing index	-	1	.160** (.084-	-.140** (-	-.281** (-	-.293** (-	-.130** (-.200-	-.258** (-.329-	-.279** (-.347-

Formatted: Justified

				.234)	.206- (-.076))	.350- (-.210))	.366- (-.215))	(-.060	(-.186))	(-.209))
3.	MHL Total mean score	-	-	1	-.135** (-.211- (-.064))	-.136** (-.212- (-.058))	-0.006 (-.078- .058)	-.088* (-.165- (-.010))	-.166** (-.244- (-.087))	-0.051 (-.120- .016)
4.	SDQ Conduct problems subscale mean score	-	-	-	1	.345** (.275- .412)	.228** (.159- .301)	.294** (.220- .368)	.812** (.785- .837)	.319** (.249- .388)
5.	SDQ Hyperactivity problems subscale mean score	-	-	-	-	1	.385** (.310- .454)	.197** (.125- .265)	.828** (.802- .854)	.380** (.310- .446)
6.	SDQ Emotional problems subscale	-	-	-	-	-	1	.272** (.195- .342)	.376** (.301- .453)	.862** (.844- .880)

Formatted: Justified

	mean score									
7.	SDQ Peer problems subscale mean score	-	-	-	-	-	-	1	.298** (.231-.362)	.721** (.683-.756)
8.	SDQ Externalizing Problems subscale mean score	-	-	-	-	-	-	-	1	.427** (.360-.489)
9.	SDQ Internalizing problems subscale mean score	-	-	-	-	-	-	-	-	1

** Correlation is significant at the 0.01 level (2-tailed); * Correlation is significant at the 0.05 level (2-tailed

Formatted: Justified

4. Discussion

The Ethiopian government has taken several interventions to mitigate the adolescent mental health issue, establishing mental health facilities nationwide.[67] The government has also trained health professionals in mental health care who are better equipped to identify and treat mental health problems in adolescents—implementing psycho-education programs in schools, public awareness campaigns, and training for community leaders and media outlets for adolescents, among others.[4] The state mechanism in promoting mental health literacy in Ethiopia is that the Ministry of Health is responsible for developing and implementing mental health policies and programs and provides training for health professionals in mental health care.[4] Stakeholders including professional associations, such as The Ethiopian Mental Health Association and The Ethiopian Psychiatric Association, are involved in policy consultation, provide training and support for health professionals, and promote mental health research in Ethiopia. These policy and programs are cascaded along the government administrative levels for actual implementation. However, there is still a paucity of evidence about the mental health status of adolescents and factors that affect mental health problems and factors that contribute for effective interventions to improve adolescents' mental health.

The SDQ is a screening tool for mental health problems in children and adolescents. It is scored on a scale of 0 to 40, with higher scores indicating more problems. The original cut-off scores for the SDQ are 80 and 90, with scores above 90 being considered abnormal, scores between 80 and 90 being considered borderline, and scores below 80 being considered normal. [27,53] The cut-off score for the present study was slightly lower for total difficulties and subscales for hyperactivity scores than the baseline and relatively higher for internalizing scores (**Table 1**). These cut-off scores for the present study were almost similar to the

Formatted: Justified

baseline definition that was taken from the UK child and adolescent population during the first version of the Strengths and Difficulties Questionnaire (SDQ) development. [27,53]

Using cut-off values from local population scores ensures cultural equivalence; that supports relevancy and applicability of the instrument to the cultural groups.[68] As indicated in Table 1, the study showed that the reference scores range taken from a present study for hyperactivity and internalizing scores were higher compared to baseline reference scores ranges. [27,53] The reference scores from a present study using the original 3 band categories (cut-off scores at 80th & 90th) for conduct problems, emotional problems, peer problems, and pro-social scores were similar to the baseline reference scores ranges.[27,53] The reference scores range taken from a present study for total difficulties, hyperactivity, and externalizing scores were lower than baseline reference scores. [27,53] If the cut-off score for a given population is higher than the original cut-off score, it means that a more significant proportion of children in that population are experiencing mental health problems.[69] This could be due to factors such as poverty, social disadvantage, or exposure to trauma.[70]

It is important to note that the SDQ is a screening tool, not a diagnostic tool.[69] If a child scores above the cut-off score, it does not necessarily mean they have a mental health problem.[69] However, it does indicate that they are at an increased risk of having a problem, and further assessment is warranted. Considering these factors is essential when interpreting a lower cut-off score from the baseline. Some evidence indicated that lower cut-off values have been more effective than the higher cut-off values at identifying child and adolescent mental health problems.[68]

These findings implied that the adolescent population in urban Ethiopia is more disadvantaged than the population on which the original cut-off scores were based. Adolescents in urban Ethiopia have been exposed to stressors like poverty, violence, or

Formatted: Justified

trauma. This adolescent population has less access to mental health services and is more likely to underreport mental health problems.

As shown on **Fig. 1,** the present finding was within the same range of the Ethiopian national level prevalence of adolescent mental health problems, reportedly ranging from 17-23%, like other study results in the Ethiopian context.[71–73] Prevalence of total difficulties (15.9-25.5%), internalizing (14.9-28.4%), emotional (10.4-23.9%), and peer relationship (17.8-25.8%) was similar that was within the range of global prevalence(10-20%).[74]

Several studies showed discrepancies in adolescent mental health problems prevalence from region to region and country to country.[70,75–77] Similarly, these differences exist across socio-demographic characteristics. [78] From the present study, mental health problems expressed by the total strength difficulties and its subscales of problems were higher among female adolescents than male adolescents. A study among Indian children, the association between the female gender and total strength difficulties score was significant.[75] Being female was associated with emotional problems.[75] A cross-national study across 73 countries on the gender gap in adolescent mental health showed that girls have worse average mental health than boys.[79] The possible explanations include but are not limited to socio-cultural factors, gender stereotypes, trauma, and abuse that disproportionally affect girls.[22,80] In contrast, conduct and hyperactivity/inattention problems were significantly more severe among males than females.[66] Consistent with other studies, the prevalence of conduct and externalizing problems was more significant among male adolescents than females. For example, a study in Northeast china showed a similar result.[78] A study among children and adolescents from seven European countries (Italy, Netherlands, Germany, Romania, Bulgaria, Lithuania, and Turkey) reported that externalized problems were consistently higher in males than females and reversed for internalized problems.[76] One possible explanation might be that mental health status is shaped by social, economic, and physical environments leading to

Formatted: Justified

inequalities that disproportionally affect gender differences and are heavily associated with risk factors for many common mental disorders.[70] According to the WHO, the relationship between the prevalence of mental health problems and poverty indices was statistically significant.[77] These poverty indices are education disparity, low income, a lack of material goods, job, and housing obstacles. For several cultural and traditional reasons, these factors impact females more than boys.[77]

Odds of mental health problems were significantly associated with some socio-demographic characteristics ($p<0.05$) differing in magnitude across these characteristics. In this study, the prevalence of mental health problems was significantly higher for females in upper elementary and lower secondary levels than for upper secondary grade levels ($p<0.05$), consistent with reports of several studies.[70,77] According to WHO report, educational status is one of the major factors determining adolescent mental health outcomes.[77]

However, the difference was insignificant for male adolescents. Furthermore, differences in prevalence have existed but little across the three age groups, maternal and paternal education level, and job types or level of employment ($p>0.05$). The present study's prevalence of mental health problems was twice higher among adolescents with a self and/or family members' experience of psychoactive substance use ($p<0.05$). On the contrary, a study in Northeast China showed that the prevalence of any mental disorders and internalizing disorders was significantly lower in younger adolescents than in elders. Children aged 11–14 years had the highest prevalence of internalizing disorders.[78] It was also inconsistent with a study in developing countries showing that lower maternal education was significantly associated with abnormal total SDQ scores.[81]

The present study revealed mental health literacy was negatively correlated with strength difficulties scores and positively correlated with mental well-being; both were significantly

Formatted: Justified

correlated but weaker in magnitude. These findings were consistent with existing theories and the results of previous studies. For instance, a study demonstrated that an inadequate level of mental health literacy was negatively associated with depression. The prevalence of depression was 1.52 times higher in those with levels of inadequate MHL than in those who demonstrated an adequate level of MHL.[50] Another study depicted MHL as an explanatory variable of the mental well-being of adolescents having a positive correlation with it. [82]

A possible explanation for the negative association between mental health literacy and strength difficulties scores might be that low mental health literacy contributes to the mental health promotion and prevention gap.[45,83–85] Low mental health literacy exacerbates barriers and difficulties in accessing treatment for mental health problems.[86]

Mental health literacy significantly influences adolescents' perceptions and emotional responses [87], reflecting mental health status and subjective mental well-being. Several studies revealed that mental health literacy had a significant relationship with mental health status. [88–90] A higher level of mental well-being has been reported among adolescents with better know-how about obtaining mental health services.[87] Adolescents' awareness of symptoms, causes, and mental illness treatment contributes to favorable attitudes toward seeking help.[15] Similarly, adolescents with better mental health literacy are less likely to engage in problematic health behaviours and have better help-seeking intention and self-efficacy, contributing to a better mental health and well-being.[91] Mental health literacy is about the information, attitudes, and awareness of mental health concerns that improve self-efficacy, help-seeking intention, and health behaviour, influencing long-term health outcomes and quality of life. A lack of understanding of the symptoms and nature of mental health disorders and alternate sources of support could explain teenagers' low help-seeking intention and behaviour. [15]

Formatted: Justified

Of course, help-seeking intention and self-efficacy may reflect only one of several mechanisms. Better mental health literacy leads to better mental well-being. Higher help-seeking intention and self-efficacy would only partially mediate the relation between higher mental health literacy and better mental well-being. Components of health behavior expressed in actions, targets, contexts, and time are the most determining factors of mental health outcomes.[92,93]

Mental health literacy influences mental health services and help-seeking for treatment.[94] Hence, mental health literacy is essential to improve and often but not always corresponds to help-seeking behaviours. [95] Anthony F. Jorm and his colleagues revealed that changing knowledge in principle is vital and not problematic; however, changing heartily emotional reactions to practice caring and preventing mental disorders may be much more challenging.[10]

Help-seeking, interchangeably health-seeking, has become one of the essential perspectives in understanding causes and factors for patient delay from appropriate action across different health conditions.[96] Despite the prevalent mental health problems and poor well-being, adolescents' unwillingness and low intention to seek help usually result in delays in timely treatment.[43] Most adolescents with mental health problems are reluctant and miss the use of healthcare for mental health, which increases the complexity of social, mental, and general health outcomes.[97] The findings from this study implied that promoting mental health literacy can improve subjective mental well-being.[87]

The recently revised policy released by The Ethiopian Ministry of Health in 2017 aimed to improve adolescents' mental health by providing early identification, prevention, and treatment services. [4,21] Following the policy, programs were launched in 2018, including school-based mental health programs, community-based mental health services, and training

Formatted: Justified

for health care providers.[21] Empirical evidence on early identification of vulnerable groups, individuals reporting symptoms of mental health difficulties and the associated actors could enhance the impact and effectiveness of these programs implementation.[4,21] In this regard, demographic and socioeconomic sensitive approaches should be applied to mental health policies for mental health promotion and mental illness prevention.

5. Limitations

The study has some inherent limitations. It is not applicable to generalize these findings across the country; because Ethiopia exhibits multicultural and multiethnic diversity and multiple socio-demographic backgrounds. It could not even be generalized to all adolescents of the study area, because the samples were selected from the age group (11-19) and grade level (5-12), adolescents at the age of 10 years and lower grade levels were excluded during the first cycle of elementary schools during school sampling. The present study is conducted on the urban adolescents and did not address rural adolescents. The study was quantitative and lacked the qualitative aspect of how and why these findings were observed.

6. Conclusion

The cut-off scores for the present study were almost similar to the baseline definition of the first version of the Strengths and Difficulties Questionnaire (SDQ) development that was taken from the UK child and adolescent population. However, the strength difficulty cut-off score for total strength difficulty and hyperactivity scores was slightly lower and the cut-off score for internalizing scores was slightly higher. The finding was within the same range as the Ethiopian national level prevalence of adolescent mental health problems, reportedly ranging from 17-23%. The prevalence of total difficulties (15.9-25.5%), internalizing (14.9-28.4%), emotional (10.4-23.9%), and peer relationship problems (17.8-25.8%) were similar

Formatted: Justified

that was slightly within the range of the global prevalence(10-20%). These mental health problems were significantly greater among adolescents who had either themselves or their family members used psychoactive substances ($p \leq 0.05$) ($p<0.05$). As depicted from strength difficulties scores, the mental health problems were significantly associated with some socio-demographic characteristics ($p<0.05$) differing in magnitude.

Female adolescents scored higher scores on the total difficulty and its subscales (internalizing and externalizing scores) than males, implying that the prevalence of mental health problems was higher among females than their male counterparts, aligning with previous study findings. These findings highlight the need to improve adolescent mental health literacy in urban Ethiopia to improve mental health status. Gender, grade level, age, maternal and paternal education level, job types, and level of employment are factors that affect adolescents' mental health problems. Hence, promoting adolescent mental health through improving mental health literacy requires congruent approaches considering these socio-demographic factors.

The study showed a correlation between mental health literacy level, the strength difficulty scale, and mental well-being scores. It showed mental health literacy negatively correlated with strength difficulties scores and positively associated with mental well-being. These findings were consistent with existing theories and the results of previous studies. It implied that promoting mental health literacy could enhance adolescents' positive mental health, and priorities should be on vulnerable groups and individuals reporting symptoms of mental health difficulties. Future studies should involve qualitative and include the remaining determinants.

Formatted: Justified

Acknowledgments

The authors would like to thank the survey respondents for their participation and contributions to the study.

Authors' contributions

Hassen, H.M and Behera M.R conceptualized the study design. Hassen, H.M collected, analyzed, and interpreted the data used in this study and was a major contributor to writing the article. Behera, D, Behera, M.R. and Dehury, R. K. assisted in analyzing and interpreting the data and writing the paper. All the authors read, commented, and approved the final article.

References

1. Renwick L, Pedley R, Johnson I, Bell V, Lovell K, Bee P, et al. Mental health literacy in children and adolescents in low- and middle-income countries: a mixed studies systematic review and narrative synthesis. Eur Child Adolesc Psychiatry [Internet] 2022;(0123456789). Available from: https://doi.org/10.1007/s00787-022-01997-6
2. Sodi T, Quarshie ENB, Oppong Asante K, Radzilani-Makatu M, Makgahlela M, Nkoana S, et al. Mental health literacy of school-going adolescents in sub-Saharan Africa: a regional systematic review protocol. BMJ Open 2022;12(9):1–7.
3. Kucera M, Tomaskova H, Stodola M, Kagstrom A. A Systematic Review of Mental Health Literacy Measures for Children and Adolescents. Adolesc Res Rev [Internet] 2023;8(3):339–58. Available from: https://doi.org/10.1007/s40894-022-00202-8
4. Ministry of Health Federal Democratic Republic of Ethiopia. Ethiopian National Mental Health Strategy (2020-2025). Fed Minist Heal 2020;2025.
5. Hassen HM, Behera MR, Jena PK, Dewey RS DG. Mental Health Literacy of Adolescents and the Effect of Socio- demographic Characteristics : A Cross-sectional Study. Online J Heal allied Sci [Internet] 2021;2–8. Available from: https://www.ojhas.org/issue80/2021-4-6.html
6. Claire M Kelly, Anthony F Jorm, Anne Marie Wright. Improving mental health literacy as a strategy [Internet]. Med. J. Aust. Med. J. Aust.2007;187(187):25–729. Available from: www.mja.com.au
7. Kelly CM, Jorm AF, Wright A. Improving mental health literacy as a strategy to facilitate early intervention for mental disorders. 2007;187(7):1–5.
8. Jorm AF. Why We Need the Concept of "Mental Health Literacy." Health Commun 2015;30(12):1166–8.
9. Jorm AF, Korten AE, Jacomb PA, Christensen H, Rodgers B, Pollitt P. "Mental health

Formatted: Justified

literacy": A survey of the public's ability to recognise mental disorders and their beliefs about the effectiveness of treatment. Med J Aust 1997;166(4):182–6.
10. Jorm A. F. et al. Research on mental health literacy: What we know and what we still need to know. Aust N Z J Psychiatry [Internet] 2006;40(1):3–5. Available from: http://www.embase.com/search/results?subaction=viewrecord&from=export&id=L43337434%5Cnhttp://dx.doi.org/10.1111/j.1440-1614.2006.01734.x%5Cnhttp://sfx.metabib.ch/sfx_locater?sid=EMBASE&issn=00048674&id=doi:10.1111/j.1440-1614.2006.01734.x&atitle=Research+
11. Patton GC, Olsson CA, Skirbekk V, Saffery R, Wlodek ME, Azzopardi PS, et al. Adolescence and the next generation. Nature [Internet] 2018;554(7693):458–66. Available from: http://dx.doi.org/10.1038/nature25759
12. Steel Z, Marnane C, Iranpour C, Chey T, Jackson JW, Patel V, et al. The global prevalence of common mental disorders: A systematic review and meta-analysis 1980-2013. Int J Epidemiol 2014;43(2):476–93.
13. O'Sullivan J and AE. Healthy Lives: Widening The Focus On Mental Health Annual Public Health Report 2015 Camden and Islington. Camden Islingt Annu Public Heal Rep 2015;1–148.
14. David K, Park MJ, Mulye TP. The Mental Health of Adolescents: A National Profile, 2008. San Francisco: Natl. Adolesc. Health Inf. Cent. (NAHIC), Univ. Calif. 2008;
15. Eschenbeck H, Lehner L, Hofmann H, Bauer S, Becker K, Diestelkamp S, et al. The Mental Health of Adolescents: A National children and adolescents with StresSOS using online or face-to-face interventions: study protocol for a randomized controlled trial within the ProHEAD Consortium. Trials [Internet] 2019;20(1):94. Available from: https://trialsjournal.biomedcentral.com/articles/10.1186/s13063-018-3157-7
16. La Maison C, Munhoz TN, Santos IS, Anselmi L, Barros FC, Matijasevich A. Prevalence and risk factors of psychiatric disorders in early adolescence: 2004 Pelotas (Brazil) birth cohort. Soc Psychiatry Psychiatr Epidemiol [Internet] 2018;53(7):685–97. Available from: http://dx.doi.org/10.1007/s00127-018-1516-z
17. Mills KL. Social development in adolescence: brain and behavioural changes [Internet]. Univ. Coll. London2015;Available from: https://discovery.ucl.ac.uk/1468865/1/KateMills_Thesis_FINAL_Appendices.pdf
18. Manganello JA. Health literacy and adolescents: A framework and agenda for future research. Health Educ Res 2008;23(5):840–7.
19. Shahraki-Mohammadi A, Panahi S, Ashouri A, Sayarifard A. Important Systemic Factors for Improving Adolescent Mental Health Literacy. Iran J Psychiatry 2023;18(1):45–54.
20. Seboka BT, Hailegebreal S, Negash M, Mamo TT, Ewune HA, Gilano G, et al. Predictors of Mental Health Literacy and Information Seeking Behavior Toward Mental Health Among University Students in Resource-Limited Settings. Int J Gen Med 2022;15(November):8159–72.
21. Hanlon C, Alem A, Lund C, Hailemariam D, Assefa E, Giorgis TW, et al. Moving towards universal health coverage for mental disorders in Ethiopia. Int J Ment Health Syst [Internet] 2019;13(1):1–17. Available from: https://doi.org/10.1186/s13033-019-0268-9
22. Eaton NR, Keyes KM, Krueger RF, Balsis S, Skodol AE, Markon KE, et al. An invariant dimensional liability model of gender differences in mental disorder prevalence: evidence from a national sample. J Abnorm Psychol 2012;121(1):282–8.
23. Fok MSMM, Wong TKSS. What does health literacy mean to children? Contemp nurse a J Aust Nurs Prof 2002;13(2–3):249–58.
24. Goodman R, Ford T, Simmons H, Gatward R, Meltzer H. Using the Strengths and

Formatted: Justified

Difficulties Questionnaire (SDQ) to screen for child psychiatric disorders in a community sample. Int Rev Psychiatry 2003;15(1–2):166–72.
25. Skinner D, Sharp C, Marais L, Serekoane M, Lenka M. Assessing the Value of and Contextual and Cultural Acceptability of the Strength and Difficulties Questionnaire (SDQ) in Evaluating Mental Health Problems in HIV/AIDS Affected Children. Int J Ment Health 2014;43(4):76–89.
26. Chongwo E, Ssewanyana D, Nasambu C, Mwangala PN, Mwangi PM, Nyongesa MK, et al. Validation of a Swahili version of the World Health Organization 5-item well-being index among adults living with HIV and epilepsy in rural coastal Kenya. Glob Heal Res Policy 2018;3(1):3–9.
27. Goodman R. The strengths and difficulties questionnaire: A research note. J Child Psychol Psychiatry Allied Discip 1997;38(5):581–6.
28. Atilola O, Singh Balhara YP, Stevanovic D, Avicenna M, Kandemir H. Self-Reported Mental Health Problems Among Adolescents in Developing Countries. J Dev Behav Pediatr [Internet] 2013;34(2):129–37. Available from: http://content.wkhealth.com/linkback/openurl?sid=WKPTLP:landingpage&an=00004703-201302000-00008
29. Goodman A, Lamping DL, Ploubidis GB. When to use broader internalising and externalising subscales instead of the hypothesised five subscales on the strengths and difficulties questionnaire (SDQ): Data from british parents, teachers and children. J Abnorm Child Psychol 2010;38(8):1179–91.
30. Downs A, Boucher LA, Campbell DG, Polyakov A. Using the WHO–5 well-being index to identify college students at risk for mental health problems. J Coll Stud Dev 2017;58(1):113–7.
31. De Wit M, Pouwer F, Gemke RJBJ, Delemarre-Van De Waal HA, Snoek FJ. Validation of the WHO-5 well-being index in adolescents with type 1 diabetes. Diabetes Care 2007;30(8):2003–6.
32. Kusier AO, Folker AP. The Well-Being Index WHO-5: Hedonistic foundation and practical limitations. Med Humanit 2019;1–7.
33. Garland AF, Deyessa N, Desta M, Alem A, Zerihun T, Hall KG, et al. Use of the WHO’s perceived Well-Being Index (WHO-5) as an efficient and potentially valid screen for depression in a low income country. Fam Syst Heal 2018;36(2):148–58.
34. Topp CW, Østergaard SD, Søndergaard S, Bech P. 1. The WHO-5 well-being index: A systematic review of the literature. Psychother Psychosom 2015;84(3):167–76.
35. Hoosen N, Davids EL, Vries PJ De, King MS. The Strengths and Difficulties Questionnaire (SDQ) in Africa : a scoping review of its application and validation. Child Adolesc Psychiatry Ment Health [Internet] 2018;1–39. Available from: https://doi.org/10.1186/s13034-017-0212-1
36. Hassen HM, Behera MR, Jena PK, Dewey RS. Effectiveness and Implementation Outcome Measures of Mental Health Curriculum Intervention Using Social Media to Improve the Mental Health Literacy of Adolescents. J Multidiscip Healthc [Internet] 2022;15(May):979–97. Available from: http://www.ncbi.nlm.nih.gov/pmc/articles/pmc9078434/
37. Maher MS and BA. Philosophy and Psychopathology. Springer-Verlag New York Inc.: Springer-Verlag New York Heidelberg Berlin London Paris Tokyo Hong Kong; 1990.
38. Brown TA, Barlow DH. Dimensional Versus Categorical Classification of Mental Disorders in the Fifth Edition of the Diagnostic and Statistical Manual of Mental Disorders and Beyond : Comment on the Special Section. 2005;114(4):551–6.
39. Tusaie KR. Epidemiology of child psychiatric disorders in Addis Ababa, Ethiopia.

Formatted: Justified

Division of Child and Adolescent Psychiatry Department of Clinical Sciences; 2013.
40. Ling J, Tay YF, Klainin-Yobas P. Mental health literacy levels. Arch Psychiatr Nurs 2018;
41. World Health Organization. Adolescents: health risks and solutions: https://www.who.int/news-room/fact-sheets/detail/adolescents-health-risks-and-solutions. 2018;
42. Polanczyk G V., Salum GA, Sugaya LS, Caye A, Rohde LA. Annual research review: A meta-analysis of the worldwide prevalence of mental disorders in children and adolescents. J Child Psychol Psychiatry Allied Discip 2015;56(3):345–65.
43. Divin N, Harper P, Curran E, Corry D, Leavey G. Help-Seeking Measures and Their Use in Adolescents: A Systematic Review. Adolesc Res Rev [Internet] 2018;3(1):113–22. Available from: http://dx.doi.org/10.1007/s40894-017-0078-8
44. Atilola O. Level of community mental health literacy in sub-Saharan Africa: Current studies are limited in number, scope, spread, and cognizance of cultural nuances. Nord J Psychiatry 2015;69(2):93–101.
45. Cortina M, Sodha A, Fazel M, Ramchandani PG. Prevalence of Child Mental Health Problems in Sub-Saharan Africa _ Global Health _ JAMA Pediatrics _ The JAMA Network. Arch Pediatr Adolesc Med 2012;166(3):276–81.
46. Zeleke WA, Nichols LM, Wondie Y. Mental Health in Ethiopia: an Exploratory Study of Counseling Alignment with Culture. Int J Adv Couns 2019;41(2):214–29.
47. CSA and UNICEF Ethiopia. Multidimensional Child Deprivation in Ethiopia. 2018;Available from: www.unicef.org/Ethiopia and http://www.csa.gov.et
48. Jorm AF. Mental health literacy. Public knowledge and beliefs about mental disorders - University College London. Br J psychiatry J Ment Sci [Internet] 2000;177:396–401. Available from: http://ucl-primo.hosted.exlibrisgroup.com/primo_library/libweb/action/display.do?tabs=detailsTab&ct=display&fn=search&doc=TN_medline11059991&indx=1&recIds=TN_medline11059991&recIdxs=0&elementId=0&renderMode=poppedOut&displayMode=full&frbrVersion=&dscnt=1&
49. Johnson RL. Pathways to adolescent health: Early intervention. J Adolesc Heal [Internet] 2002;31(6 SUPPL.):240–50. Available from: http://ovidsp.ovid.com/ovidweb.cgi?T=JS&PAGE=reference&D=emed5&NEWS=N&AN=2002445167
50. Lam LT. Mental health literacy and mental health status in adolescents: A population-based survey. Child Adolesc Psychiatry Ment Health 2014;8(1):1–8.
51. Hassen HM, Behera MR, Jena PK, Satpathy SK. Validity and Reliability of the Amharic Version of the HLS-EU-Q47 Survey Questionnaire among Urban School Adolescents and University students in Dire Dawa, Ethiopia. Prepr Res Sq 2020;
52. Kersten P, Czuba K, McPherson K, Dudley M, Elder H, Tauroa R, et al. A systematic review of evidence for the psychometric properties of the Strengths and Difficulties Questionnaire. Int J Behav Dev 2016;40(1):64–75.
53. Mathai J, Anderson P, Bourne A. The Strengths and Difficulties Questionnaire (SDQ) as a screening measure prior to admission to a Child and Adolescent Mental Health Service (CAMHS). Aust e-Journal Adv Ment Heal 2002;1(3):235–46.
54. Heizomi H, Kouzekanani K, Jafarabadi MA, Allahverdipour H. Psychometric properties of the persian version of mental health literacy scale. Int J Womens Health 2020;12:513–20.
55. O’Connor M, Casey L, Clough B. Measuring mental health literacy-a review of scale-based measures. J Ment Heal 2014;23(4):197–204.
56. Campos L, Dias P, Palha F, Duarte A, Veiga E. Development and psychometric

Formatted: Justified

properties of a new questionnaire for assessing mental health literacy in young people. Univ Psychol 2016;15(2):61–72.
57. Bale J, Grové C, Costello S. I. Ment Heal Prev [Internet] 2018;12(February):26–35. Available from: https://doi.org/10.1016/j.mhp.2018.09.003
58. Lam LT. Mental health literacy and mental health status in adolescents: a population-based survey. Child Adolesc Psychiatry Ment Health 2014;8(26):1–8.
59. Dias P, Campos L, Almeida H, Palha F. Mental health literacy in young adults: Adaptation and psychometric properties of the mental health literacy questionnaire. Int J Environ Res Public Health 2018;15(7).
60. Wei Y, McGrath PJ, Hayden J, Kutcher S. Mental health literacy measures evaluating knowledge, attitudes and help-seeking: A scoping review. BMC Psychiatry [Internet] 2015;15(1). Available from: http://dx.doi.org/10.1186/s12888-015-0681-9
61. Tomé G, Gaspar de Matos M, Camacho I, Gomes P, Reis M, Branquinho C. Mental health promotion in school context – Validation of the ES ’ COOL scale for teachers. J Psychiatry Behav Sci [Internet] 2018;2:(1009). Available from: http://meddocsonline.org/
62. Bjørnsen HN, Eilertsen MEB, Ringdal R, Espnes GA, Moksnes UK. Positive mental health literacy: Development and validation of a measure among Norwegian adolescents. BMC Public Health 2017;17(1):1–10.
63. Reilly MO, Svirydzenka N, Adams S, Dogra N. Review of mental health promotion interventions in schools. Soc Psychiatry Psychiatr Epidemiol [Internet] 2018;53(7):647–62. Available from: http://dx.doi.org/10.1007/s00127-018-1530-1
64. Hoofs H, Jansen NWH, Mohren DCL, Jansen MWJ, Kant IJ. The context dependency of the self-report version of the Strength and Difficulties Questionnaire (SDQ): A cross-sectional study between two administration settings. PLoS One [Internet] 2015;10(4):1–21. Available from: http://dx.doi.org/10.1371/journal.pone.0120930
65. Koskelainen M, Sourander A, Kaljonen A. The Strengths and Difficulties Questionnaire among Finnish school-aged children and adolescents. Eur Child Adolesc Psychiatry 2000;9(4):277–84.
66. De Vries PJ, Davids EL, Mathews C, Aarø LE. Measuring adolescent mental health around the globe: Psychometric properties of the self-report Strengths and Difficulties Questionnaire in South Africa, and comparison with UK, Australian and Chinese data. Epidemiol Psychiatr Sci 2018;27(4):369–80.
67. Hughes TL, Quinn C, Tiberi A, Zeleke WA. Developing a Framework to Increase Access to Mental Health Services for Children With Special Needs in Ethiopia. Front Sociol 2020;5(December):1–6.
68. Renshaw TL. Screening Using the SDQ Total Difficulties Scale: An Analog Test of Three Cutoff Values. J Psychoeduc Assess 2019;37(8):1030–6.
69. Editor S, Kamphaus RW. Contemporary Issues in Psychological Assessment. Springer Berlin Heidelberg; 2015.
70. Carod-Artal FJ. Social determinants of mental health. Glob Ment Heal Prev Promot 2017;(2):33–46.
71. FDREMH. Federal Democratic Republic of Ethiopia Ministry of Health: National Mental Health Strategy (2012/13- 2015/16). Heal Sect Dev Program IV 2010/11 – 2014/15 2011;(October 2010):1–114.
72. Lu W. Child and Adolescent Mental Disorders and Health Care Disparities: Results from the National Survey of Children’s Health, 2011– 2012. 2017;28:988–1011.
73. Dachew BA, Bifftu BB, Tiruneh BT, Anlay DZ, Wassie MA, Betts K. Prevalence and determinants of mental distress among university students in Ethiopia : a systematic review protocol. 2019;8–11.

Formatted: Justified

74. Baker-henningham H, Belfer M, Conti G, Ertem I, Omigbodun O, Rohde LA, et al. Global Mental Health 2 Child and adolescent mental health worldwide : evidence. Lancet [Internet] 2011;378(9801):1515–25. Available from: http://dx.doi.org/10.1016/S0140-6736(11)60827-1
75. Nair S, Ganjiwale J, Kharod N, Varma J, Nimbalkar SM. Epidemiological survey of mental health in adolescent school children of Gujarat, India. BMJ Paediatr Open 2017;1(1):e000139.
76. Kovess V, Carta MG, Pez O, Bitfoi A, Koc C, Goelitz D, et al. The School Children Mental Health in Europe (SCMHE) Project: Design and First Results. Clin Pract Epidemiol Ment Heal 2015;11(1):113–23.
77. Patel V, Kleinman A. Poverty and common mental disorders in developing countries. Bull World Health Organ [Internet] 2003;81(8):609–15. Available from: http://www.ncbi.nlm.nih.gov/pubmed/14576893%0Ahttp://www.pubmedcentral.nih.gov/articlerender.fcgi?artid=PMC2572527
78. Lijuan T, Chao J, Wenming X, Huijuan M, Lianzheng Y, Ming L, et al. Prevalence of Psychiatric Disorders among Children and Adolescents in Northeast China. PLoS One 2014;9(10):e111223.
79. Campbell OLK, Bann D, Patalay P. SSM - Population Health The gender gap in adolescent mental health : A cross-national investigation of 566 , 829 adolescents across 73 countries. SSM - Popul Heal [Internet] 2021;13(December 2020). Available from: https://doi.org/10.1016/j.ssmph.2021.100742
80. Tedstone Doherty D, Kartalova-O'Doherty Y. Gender and self-reported mental health problems: Predictors of help seeking from a general practitioner. Br J Health Psychol 2010;15(1):213–28.
81. Atilola O, Pal Y, Balhara S, Avicenna M. Self-Reported Mental Health Problems Among Adolescents in Developing Countries : Results from an International Pilot. J Dev Behav Pediatr 2013;34(2):129–37.
82. Bjørnsen HN, Espnes GA, Eilertsen MEBEB, Ringdal R, Moksnes UK. The Relationship Between Positive Mental Health Literacy and Mental Well-Being Among Adolescents: Implications for School Health Services. J Sch Nurs [Internet] 2019;35(2):107–16. Available from: http://journals.sagepub.com/doi/10.1177/1059840517732125
83. World Health Organization. WHO Adolescent mental health [Internet]. 2018;Available from: http://www.who.int/news-room/fact-sheets/detail/adolescent-mental-health
84. Belfer ML. Child and adolescent mental disorders: The magnitude of the problem across the globe. J Child Psychol Psychiatry Allied Discip 2008;49(3):226–36.
85. Atilola O, Pal Y, Balhara S, Avicenna M, Singh Balhara YP, Stevanovic D, et al. Self-Reported Mental Health Problems Among Adolescents in Developing Countries. J Dev Behav Pediatr [Internet] 2013;34(2):129–37. Available from: http://content.wkhealth.com/linkback/openurl?sid=WKPTLP:landingpage&an=00004703-201302000-00008
86. Ibrahim N, Mohd Safien A, Siau CS, Shahar S. The Effectiveness of a Depression Literacy Program on Stigma and Mental Help-Seeking Among Adolescents in Malaysia: A Control Group Study With 3-Month Follow-Up. Inq (United States) 2020;57.
87. Bjørnsen HN, Espnes GA, Eilertsen MEB, Ringdal R, Moksnes UK. The Relationship Between Positive Mental Health Literacy and Mental Well-Being Among Adolescents: Implications for School Health Services. J Sch Nurs 2019;35(2):107–16.
88. Lam LT. Mental health literacy and mental health status in adolescents: a population-based survey. Child Adolesc Psychiatry Ment Health [Internet] 2014;8(26):126–9.

Formatted: Justified

Available from: http://www.capmh.com/content/8/1/26
89. Wu C, Liu S, Chang S. Surveys of medical seeking preference , mental health literacy , and attitudes toward mental illness in Taiwan , 1990 e 2000. J Formos Med Assoc [Internet] 2014;113(1):33–41. Available from: http://dx.doi.org/10.1016/j.jfma.2013.03.004
90. Furnham A, Lee V, Kolzeev V. Mental health literacy and borderline personality disorder (BPD): what do the public “make” of those with BPD? Soc Psychiatry Psychiatr Epidemiol 2015;50(2):317–24.
91. Oldfield J, Stevenson A, Ortiz E, Haley B. Promoting or suppressing resilience to mental health outcomes in at risk young people: The role of parental and peer attachment and school connectedness. J Adolesc 2018;64(April 2017):13–22.
92. Presseau J, Mccleary N, Lorencatto F, Patey AM, Grimshaw JM, Francis JJ. Action , actor , context , target , time (AACTT): a framework for specifying behaviour. 2019;1–13.
93. Kan MPH, Fabrigar LR, Fishbein M. Theory of Planned Behavior. 2017;1–8.
94. Kessler RC, M. A, Anthony JC, De Graaf R, Demyttenaere K, Gasque I, et al. Lifetime prevalence and age-of-onset distributions of mental disorders in the World Health Organization’ s. World Psychiatry 2007;6:168-176) 2007;6(October):168–76.
95. Wei Y, Mcgrath PJ, Hayden J, Kutcher S, Wei Y, Mcgrath PJ, et al. Measurement properties of mental health literacy tools measuring help-seeking : a systematic review. J Ment Heal [Internet] 2017;0(0):000. Available from: http://dx.doi.org/10.1080/09638237.2016.1276532
96. Cornally N, Mccarthy G. Help-seeking behaviour: A concept analysis. Int J Nurs Pract 2011;17(3):280–8.
97. Bonabi H, Müller M, Ajdacic-Gross V, Eisele J, Rodgers S, Seifritz E, et al. Mental health literacy, attitudes to help seeking, and perceived need as predictors of mental health service use: A longitudinal study. J Nerv Ment Dis 2016;204(4):321–4.

Formatted: Justified

Manuscript Click here to access/download;Manuscript;Manuscript.docx

Original Research

Mental health issues and the association of mental health literacy among adolescents in urban Ethiopia

Hailemariam Mamo Hassen[1*], Manas Ranjan Behera[2], Deepanajli Behera[2], Ranjit Kumar Dehury[3]

[1]Department of Biological Sciences, Dire Dawa University, Dire Dawa, Ethiopia

[2] School of Public Health, KIIT Deemed to be University, Bhubaneswar, India

[3]School of Management Studies, University of Hyderabad, Telangana, India

* hailemariammamo5@gmail.com

Abstract

Background

Epidemiological evidence about the prevalence of adolescent mental health issues and their association with mental health literacy is crucial for sustained mental health promotion strategies. Adolescence is a critical life stage for mental health promotion. However, evidence is not available among Ethiopian school adolescents. Hence, the present study examined the prevalence of adolescents' mental health issues and their correlation with mental health literacy.

Materials and methods

A cross-sectional study was conducted among adolescents (grades 5-12) in Dire Dawa city, Eastern Ethiopia using multistage random sampling. Data was collected using the Strength and Difficulty Questionnaire, WHO-5 well-being index, and mental health literacy questionnaire**.** SPSS version 25 was used for the descriptive, Chi-square, binary logistic regression, and correlation analyses.

Results

About 15.9-28.4% of adolescents had reported mental health problems: internalizing problems (14.9-28.4%), emotional problems (10.4-63.9%), and peer relationship problems (17.8-25.8%). These mental health problems were significantly greater among adolescents who had either themselves or their family members used psychoactive substances ($p \leq 0.05$). Females from upper elementary (5-8 grade) and lower secondary (9-10) grade levels had a higher prevalence of mental health problems (AOR: 2.60 (0.95-7.10, $p<0.05$)). The effect of age, parental education, or employment status was insignificant ($p>0.05$). The prevalence of depression ranged from 18.0-25.5%. Mental health literacy was negatively correlated with total difficulties scores and positively associated with mental well-being scores ($p<0.05$).

Conclusion

The prevalence of adolescents' mental health problems was higher. It implied that promoting mental health literacy could enhance adolescents' positive mental health. Intervention programs should prioritize vulnerable groups and individuals reporting symptoms of mental health difficulties. Future studies should involve qualitative studies and consider effect of other determinants.

1. Introduction

Globally, mental health problems disproportionately affect adolescents in low-income countries and they remain the leading causes of disease burden among children and young people.[1] Knowing about these mental health conditions, their symptoms, and treatments termed as mental health literacy is essential for everyone but is crucial for adolescents.[2] Mental health literacy is defined as understanding and applying information about mental health. [2] In this regard, much has been known about the mental health literacy of adolescents from high-income countries.[3] However, it is still a relatively new concept in Africa.[2] Additionally, studies revealed that access to mental health information or services in Africa is lower due to poverty, lack of education, and cultural stigma around mental illness.[1] Nevertheless, limited evidence in Africa, including Ethiopia, has indicated a growing awareness about the importance of mental health literacy. Despite the challenges associated with poverty, cultural stigma, lack of access to health facilities, and lower education coverage, countries are working to improve mental health literacy.[2]

The Ethiopian Ministry of Health has developed a mental health policy focusing on primary mental healthcare and promoting positive mental health, including a mental health awareness campaign[4]. The present study area is Dire Dawa, one of Ethiopian provinces known for its ethnic and cultural language diversity. A recent study revealed that adolescent mental health literacy from Dire Dawa city was lower, consistent with similar studies reports and showing variability across socio-demographic factors. [5]

Epidemiological evidence about the prevalence of mental health problems and their association with mental health literacy is crucial for sustained mental health promotion strategies, as adolescence is a critical life stage for mental health promotion.[6–10] However, such evidence is rarely available among Ethiopian school adolescents. Adolescent mental

health has piqued the interest of public health professionals due to the unique nature of adolescence.[11] Therefore, adolescence remains a critical life stage for mental health promotion.[11] A popular saying goes, “there is no health without mental health.” [12] With multifaceted burdens, mental health is integrally linked to physical health and well-being.[13] The typical adolescent mental health problems include but are not limited to: substance use/abuse or disorders, depression/mood disorders, anxiety, schizophrenia, stress/neurosis, behavioral disorders, postpartum psychosis, and posttraumatic stress disorder (PTSD). [14,15] Several studies showed that these mental health problems in adulthood occur during adolescence. [14,15]

For multiple reasons, adolescents are among the most important groups for mental health studies. For example, adolescence is key to physical, emotional, and cognitive progress.[11,16,17] Fundamentally, mental, physical, and emotional developmental processes evolve during adolescence. [18] Also public health perspectives emphasizing mental health information during early childhood,[18] would help in later life in managing and preventing mental health problems.[18]

Mental health literacy is essential to preventing mental illnesses, consisting of knowledge about mental health problems and recognizing illnesses with their symptoms.[1,3] Studying the mental health status and its association with the mental health literacy of adolescents is paramount because it helps understand these variables across varied cultural settings.[1] The peak of the onset of mental disorders mainly occurs in the adolescent period and needs urgent attention. [19] In low-resource countries like Ethiopia, risk factors such as cigarette smoking, alcohol consumptions, early co-habitation, unhealthy lifestyles etc are higher among adolescents.[20,21] leading to various mental health problems.[11,16,17] Hence,

epidemiological methods like cross-sectional studies provides empirical evidence about the mental health status and factors that contribute to mental health problems.[22] Early understanding of these evidence contribute for making informed and effective interventions to improve adolescents' mental health. [23]

As a result, there is an increasing demand for adolescents’ mental health and well-being outcome measures to inform the public and policymakers regarding individual or service-level health promotion and therapeutic practices. In this regard, the strength difficulties questionnaire (SDQ) and the mental well-being index (WHO-5) have been essential to meet the criteria of valid and reliable measures.

The Strengths and Difficulties Questionnaire (SDQ) and the WHO-5 Well-Being Index are commonly used to assess adolescents' mental health.[24–34] The SDQ is a 25-item self-report questionnaire that assesses five mental health domains: conduct problems, emotional symptoms, peer problems, hyperactivity-inattention, and pro-social behavior. [24,25,27–29] The WHO-5 is a 5-item self-report questionnaire that assesses overall well-being. [26,30–34] Both instruments are good choices for assessing adolescent mental health in low-resource settings because they are brief, relatively inexpensive, and easy to administer, and shown to be reliable and valid in adolescents from low-resource settings have been validated in a variety of settings and translated into multiple languages for low-resource settings including in Ethiopia[35,36] Therefore, the SDQ and the WHO-5 are good choices for assessing adolescent mental health in low-resource settings. In this article, the concept of “normalcy” and “mentally sick” as two separate spheres was considered by taking the categorical approach than the dimensional approach of measuring mental health status. [37,38]

The assessment of mental health issues using the familiar Diagnostic Standard Manual – 5 (DSM-5) and International Classification of Disease (ICD-11) analysis has been published in

previous studies.[37,38] Such an approach is marked by clustering disorders, namely, externalizing and internalizing problems. Instruments with high validity are routinely used to measure the internalizing groups (i.e., depressive, somatic, and anxiety symptoms) and the externalizing groups (substance use, disruptive, and conduct symptoms). Furthermore, the well-being state was examined using the WHO-5 index, with lower scores indicating depression.

The daily suffering of people with mental illness affects family members, mainly children, and adolescent populations.[39] Globally, about 25% of the total population and 10-20% of children and adolescents have mental disorders, of which half of all these mental health problems begin by the age of fourteen and three-fourths by the age of early twenties.[40,41] A worldwide-pooled prevalence meta-analysis on mental disorders from all regions of the world addressing 27 countries showed a prevalence of 13.4% ranging from 11.3 to 15.9 (CI: 95%), which indicates mental disorders is affecting a large proportion of the child and adolescent population.[42] According to studies that used the strengths and difficulties questionnaire (SDQ), around 10.5% (5.8-15%) of adolescents in developing countries had mental health concerns.[28]

The prevalence of mental health problems among adolescents in Africa including Ethiopia, was reportedly higher.[43–47] For instance, a meta-analysis study on adolescents in sub-Saharan Africa, including Ethiopia (N=9713), showed the proportion of adolescents having psychopathology was 14.3% (13.6%-15.0%), and the prevalence of psychological disorders was 19.8% (18.8%-20.7%).[45]

These mental health problems are exacerbated by modifiable factors such as low mental health service seeking, lower mental health service use, stigma, discrimination, and common human rights abuses. These factors are linked to immediate and intermediate mental health

outcomes such as health literacy and behaviour. Mental health literacy is a changeable factor linked to improved psychological outcomes.[6–10] It refers to how well a person understands mental diseases and the associated factors.[6–10] Mental health literacy includes an individual's potential to identify mental distress.[9,48,49] Some qualitative descriptions revealed that mental health difficulties and mental well-being is associated with mental health literacy, despite the lack of quantitative evidence of the correlation between mental health literacy, mental health status, and mental well-being.[30]

There has been a scarcity of available epidemiological evidence about mental health conditions and literacy among Ethiopian school adolescents. There was also a lack of evidence about school adolescents' mental health status and the effect of socio-demographic characteristics in the existing literature. Therefore, the objectives of the study were:

- To assess the prevalence of mental health issues among urban adolescents in Ethiopia
- To evaluate the relationship between mental health literacy and mental health issues and
- To examine the impact of socio-demographic characteristics on mental health issues

2. Materials and methods

2.1. Study design and sampling

The present research was a cross-sectional study among public and private school adolescents in grades 5 through 12 from 11 to 19 years in Dire Dawa, Ethiopia. A combination of multistage (schools, classrooms, then individual students) and systematic and random (using the list of the students in fixed intervals of their roll numbers) was used to select study participants from public and private schools. . The prevalence of self-reported mental health

difficulty was obtained from the pilot study of the same population (p=0.378) [51], and the margin of sampling error (d=0.04) was taken to calculate the sample size.

$$n \geq \frac{(Za/2)^2 \text{ x } P(1-P)}{d^2} = \frac{1.96^2 \text{ x } 0.378(1-0.378)}{0.04^2} = 559.673 \approx 560$$

Data was collected from June 2020 to July 2020 using the mental health literacy questionnaire (MHLQ), the Strength and Difficulty Questionnaire (SDQ), and the WHO-5 well-being index after cross-cultural validation in the study settings. Maximum sample size estimation (n= 924) was approached after taking design effect (d=1.5) and a 10% non-response. About 751 participants filled out the questionnaires out of the 924 potential participants.

2.2. Data collection tools

Data collection tools were the Strengths and Difficulties Questionnaire (SDQ), mental health literacy questionnaire (MHLQ), and mental well-being index (WHO-5). The predictor variables mainly focusing on age, grade level, self or any family member experience with psychoactive substance use, parents practicing corporal punishment, perceived worry about family problems, parents education, and job status were collected by using questionnaires. The Strengths and Difficulties Questionnaire (SDQ) is a valid, rapid measure for emotional and behavioral problems worldwide.[24,25,27–29] SDQ is a 25-item, 3-point Likert scale (0=not true, 1=somewhat true, and 2=certainly true). It measures subscale difficulty problems that are emotional(SDQ3,8,13,16&24), conduct (SDQ5,7,12,18&22), hyperactivity/inattention(SDQ 2,10,15,21&25), peer(SDQ6,11,14,19&23) and pro-social behaviour (SDQ 1,4,9,17&20) problems.[52] The total difficulties score combines conduct, hyperactivity, emotional, and peer relationship behaviour problems. The externalizing

problem manifests emotional and peer behavioural relationship problems. Meanwhile, the internalizing problem manifests the conduct and hyperactivity/inattention problems.

Adding up the first four subscales leads to total difficulty scores (the higher the total difficulties score, the more significant the mental health difficulty).[52] The fifth sub-scale of the SDQ reflects pro-social behaviour (the higher the score, the better the pro-social behaviour).[52] SDQ is widely used in resource-poor countries to measure children and adolescents' mental and emotional problems. The baseline for cut-off scores definition of total and subscales strength and difficulty scores were from the United Kingdom population norms defined during the instrument development. [27,53] The self-administered SDQ is a validated and widely used measurement tool for determining mental health problems in children and adolescents.[24,25,27–29] It has already been translated into over 60 languages, including Ethiopian Amharic.[24,25,27–29] It has reportedly been an effective tool for assessing children's mental health in a recent scoping review in Africa. [35]

Mental health literacy was measured using the mental health literacy questionnaire (MHLQ). This MHLQ tool is freely available and validated for adults, [54] and adolescents [7,55–63] living in low-income countries to measure health literacy scores. In the context of the current investigation, its reliability was evaluated using Cronbach's alpha and found to be 0.834. MHLQ has 33 items with five Likert scale ratings *(1=strongly disagree, 2=slightly disagree, 3=neither agree nor disagree, 4=slightly agree, 5=strongly agree).* The MHLQ consists of 33 questions that measure factors like recognition (10 questions), knowledge (8 questions), attitudes (8 questions), and beliefs (7 questions) of the adolescents on mental health status. [6–10] The range of scores for the mental health literacy tool is 33 to 165. A higher score implies a better level of mental health literacy.

The mental well-being index (WHO-5) is an overarching expression of the quality of the various domains in the life of adolescents subjective to their mental and psychological well-being. Mental well-being was assessed using the WHO's well-being index, which consists of 5 items (WHO-5). [26,30–34] It is a Likert-type scale with a five-point scale (5=all of the time, 4=most of the time, 3=more than half the time, 2=less than half the time, 1= some of the time, 0=at no time).

2.3. Ethical approval

Ethical approval was obtained from KIIT University and Haramaya University. Written informed consent was obtained from all participants. Written informed consent was obtained from parents of adolescents aged under fifteen years had offered their consent and adolescents of fifteen and above years were factored out following the school counsellors and school principals adequate explanation about their level of maturity and decisional capacity. As a duly authorized representative, written informed consent was obtained from school principals.

2.4. Statistical analysis

SPSS version 25 was used to carry out the statistical analysis. Descriptive analysis was done to present the socio-demographic characteristics, cut-off score determination, and prevalence of mental health problems. Chi-square test and binary logistic regression analysis were used to estimate the difference and compare the mental health problems prevalence between males and females across some socio-demographic characteristics. Correlation analysis was performed to assess the associations between mental health literacy, strength difficulties questionnaire scale, and subjective mental well-being. Estimates used a confidence interval of 95 % ($p \leq 0.05$).

The cut-off score for mental health problems(cases) was defined using the original 3 band categorizations (cut-off scores at 80th & 90th).[64–66] Scores of every item in the scale are added from 0–10. The added scores (range of 0–40) were calculated by adding scores on the emotion components, conduct problems, hyperactivity, inattention, and peer problem scales.[27–29] The cut-off scores was defined by percentile into the highest first 10% (abnormal), the next 10% (borderline), and the remaining 80% (normal). The cut-off for mental health problems (caseness) was the highest at first 10 %.

3. Results

3.1. The socio-demographic characteristics of study participants

About 751 participants comprising 50.1% males and 49.9% females, completed the questionnaires, and statistical analysis was performed for the 731 participants after removing 2.66% of respondents from the analysis for significant missing data. It did not affect the sample power because a greater number of participants were participated compared to the calculated minimum sample size (n=565). The mean age with standard deviation of male and female respondents was 16.27 ± 2.19 and 15.95 ± 2.02, respectively. The age range for adolescents and grouping was based on UNICEF adolescents' age categorization as early, middle, and late adolescence age groups, which is helpful to target mental health promotion and prevention efforts during these stages to address their respective mental health demands.

3.2. Strength difficulty scores and prevalence of mental health status

The original 3 bands (cut-off scores at 80th & 90th) and the newer 4 band (cut-off scores at 80th, 90th & 95th) categorization were reported just for comparison as reported in **Table 1**. The original 3 band was used to determine and estimate adolescents' mental health issues. The mental health problems expressed with the total difficulties and in subscales, in general, were higher for female adolescents than male adolescents. The prevalence of mental health problems (cases) was presented across gender and age categories.

Table 1: The cut of scores for baseline reference ranges (caseness) for both the original 3 band and the newer 4 band categorizations

Self-completed SDQ	Original 3 band categories(cut-off scores at 80th & 90th)						Newer 4 band categorisations (cut-off scores at 80th, 90th & 95th)							
	Normal		Borderline		Abnormal		Close to average		Slightly raised slightly lowered		High (/low)		Very high (/very low	
	Baseline norm	Present study range	Baseline norm	Present study range	Baseline norm	Present study ran	Baseline norm	Present study ran	Baseline norm	Present study ran	Baseline norm	Present study ran	Baseline norm	Present study range

						ge		ge		ge		ge		
Total difficulties score	0-15	0-14	16-19	15-17	20-40	18-40	0-14	0-14	15-17	15-17	18-19	18-20	20-40	20-40
Emotional problems score	0-5	0-5	6	6	7-10	7-10	0-4	0-5	5	6	6	7	7-10	8-10
Conduct problems score	0-3	0-3	4	4	5-10	5-10	0-3	0-3	4	4	5	5	6-10	6-10
Hyperactivity score	0-5	0-4	6	5	7-10	6-10	0-5	0-4	6	5	7	6	8-10	7-9
Peer problems score	0-3	0-4	4-5	5	6-10	6-10	0-2	0-4	3	5	4	6	5-10	7-9
Pro-social score	6-10	6-10	5	5	0-4	0-4	7-10	7-10	6	6	5	5	0-4	0-4
Externalising	0-5	0-5	6-10	6-7	11-20	8-20	0-5	0-5	6-10	6-7	11-12	8	13-20	9-20

score														
Internalising score	0-4	0-8	5-8	9-10	9-20	11-20	0-4	0-8	5-8	9-10	9-10	11-12	11-20	13-20

As indicated in **Fig 1**, the prevalence of mental health total difficulties (15.9-25.5%), internalising (14.9-28.4%), emotional (10.4-23.9%), and peer relationship (17.8-25.8%) problems were higher compared to reports of previous studies. These mental health problems were more prevalent for female and male adolescents compared to other age groups in extent of percentage as explicitly visualized in Figure 1 across the three age groups. Prevalence of depression ranged from 18.0%-25.5%, reportedly higher for female adolescents aged 14-16 years.

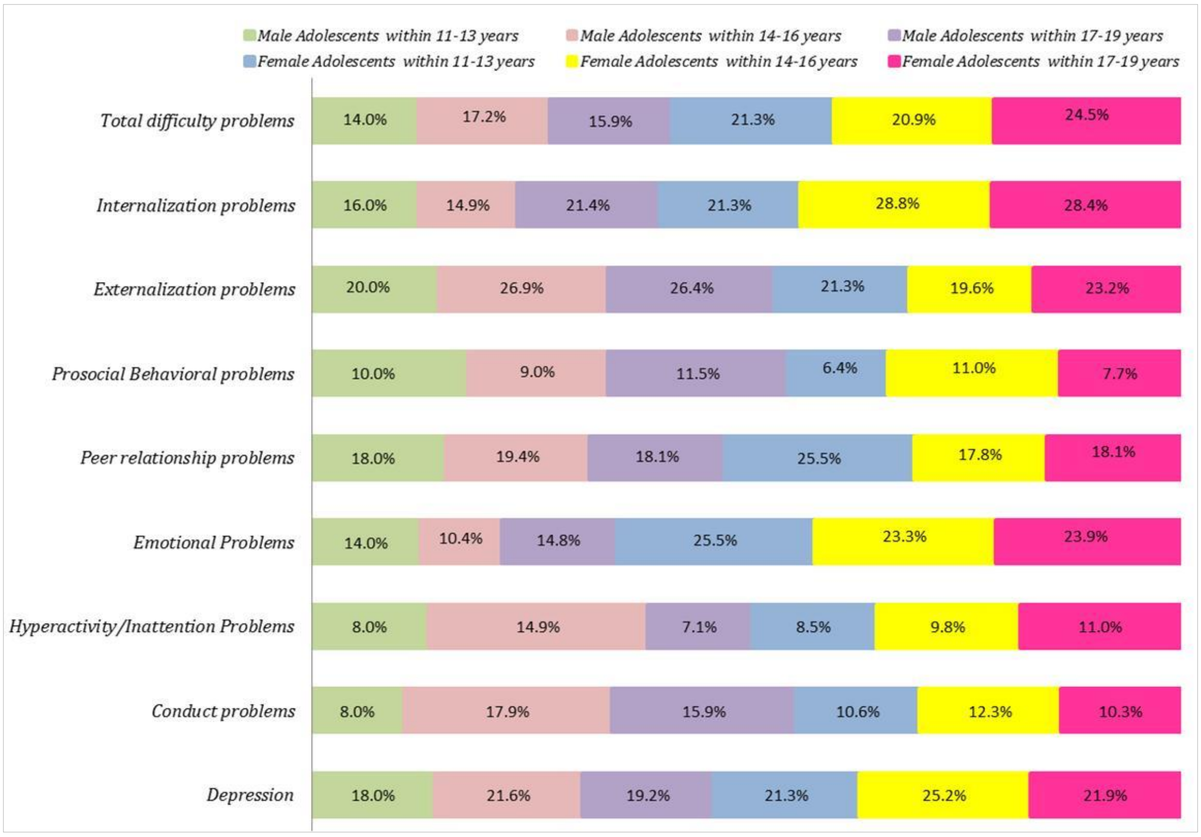

Figure 1: Prevalence of mental health problems among school adolescents in Dire Dawa, Ethiopia.

3.3. Association of mental health status and socio-demographic factors

Statistical difference in prevalence was examined for each banding of subscale using the Chi-square test. The proportion of abnormal females with total difficulties and emotional and internalizing problems was significantly higher ($p<0.05$) than males. The prevalence of conduct and externalizing problems was highly associated with being male. The association of socio-demographic characteristics with mental health status was analyzed using binary logistic regression as reported in **Table 2**.

Table 2: Chi-square test of mental health problems prevalence as per the original 3 band categorization (cut-off scores at 80th & 90th) between males and females

Subscales and bandings	Males (n=366)		Females (n=365)		df	X^2	p
	n	%	n	%			
Total difficulties					2	6.913[a]	0.042
Normal(0-14)	307	83.9%	283	77.5%			
Borderline(15-17)	29	7.9%	48	13.2%			
Abnormal(18-40)	30	8.2%	34	9.3%			
Emotional problems					2	14.442[a]	0.001

score							
Normal0-5)	318	86.9%	278	76.2%			
Borderline=6	23	6.3%	36	9.9%			
Abnormal(7-10)	25	6.8%	51	14.0%			
Conduct problems							
Normal(0-3)	309	84.4%	324	88.8%	2	3.674[a]	0.159
Borderline=4	30	8.2%	18	4.9%			
Abnormal(5-10)	25.0	7.4%	25.0	6.3%			
Hyperactivity score					2	0.488[a]	0.783
Normal (0-4)	329	89.9%	328	89.9%			
Borderline=5	21	5.7%	18	4.9%			
Abnormal(6-10)	16	4.4%	19	5.2%			
Peer problems score					2	0.930[a]	0.628
Normal(0-4)	298	81.4%	296	81.1%			
Borderline=5	41	11.2%	36	9.9%			
Abnormal(6-10)	27	7.4%	33	9.0%			
Internalising score					2	9.332[a]	0.009
Normal(0-8)	299	81.7%	264	72.3%			
Borderline(9-10)	36	9.8%	50	13.7%			
Abnormal(11-20)	31	8.5%	51	14.0%			
Externalising score					2	1.896[a]	0.387
Normal(0-5)	272	74.3%	287	78.6%			

Borderline(6-7)	50	13.7%	41	11.2%			
Abnormal(8-20)	44	12.0%	37	10.1%			
Pro-social score					2	0.549[a]	0.760
Normal(6-10)	328	89.6%	332	91.0%			
Borderline=5	18	4.9%	14	3.8%			
Abnormal(0-4)	20	5.5%	19	5.2%			

A binary logistic regression model analysis showed that the odds of mental health problems were significantly associated with some socio-demographic characteristics (p<0.05); odds were different across these characteristics as reported in **Table 3**. The prevalence of mental health problems was significantly higher for females in upper elementary (AOR: 2.60 (0.95-7.10)) and lower secondary levels (AOR: 2.73 (1.19-6.29)) compared to upper secondary grade level (p<0.05); but it was not significantly different for males. It was twice higher among adolescents with a self and family members experiencing psychoactive substance use (p<0.05). Prevalence difference across age, maternal and paternal education, and employment status was insignificant (p>0.05).

Table 3: Association of mental health problems and socio-demographic determinants analyzed with binary logistic regression

		Prevalence of mental health problem %			
Predictors		Male		Female	
	Categories	UAOR(95%	AOR(95% CI)	UAOR(95%	AOR(95% CI)

		CI)		CI)	
	11-19	-	-		-
Age group(yrs)	11-13	1	1	1	1
	14-16	1.27(0.51-3.18)	0.959(0.25-3.7)	0.98 (0.44-2.16)	0.65 (0.20-2.06)
	17-19	1.16(0.48-2.84)	0.643(0.06-6.28)	1.20 (0.55-2.64)	0.53 (0.08-3.38)
Grade level	Upper elementary (5-8)	1.08(0.51-2.31)	1.44(0.50-4.15)	1.57 (0.70-3.50)	2.60 (0.95-7.10)
	Lower Secondary (9-10)	1.02(0.48-2.18)	1.11(0.50-2.46)	2.22 (1.00-4.92)*	2.73 (1.19-6.29)*
	Upper secondary (11-12)	1	1	1	1
Experience on psychoactive substances use					
Self-experience	No	1	1	1	1
	Yes	0.45(0.22-0.93*	2.20(1.07-4.52)*	2.17 (0.95-4.95)	2.16 (0.95-

					4.92)
Any family experience	No	1	1	1	1
	Yes	2.01(1.01-4.00)*	2.04(1.01-4.09)*	1.07(0.63-1.84)	1.06 (0.62-1.81)
Parents practicing corporal punishment	No	1	1	1	1
	Yes	1.24 (0.69-2.24)	1.24 (0.69-2.23)	1.43 (0.82-2.51)	1.33 (0.75-2.36)
Adolescents report perceived worry about family problem	No	1	1	1	1
	Yes	1.186(0.64-2.21)	1.17(0.62-2.20)	1.24 (0.73-2.12)	1.20 (0.70-2.06)
Mother education level	Non-educated	1	1	1	1
	Elementary	1.31(0.50-3.43)	1.30 (0.49-3.41)	1.99 (0.57-6.96)	0.73 (0.29-1.87)
	Secondary	1.30(0.66-2.57)	1.30 (0.66-2.58)	1.50 (0.34-6.70)	1.37 (0.78-2.42)
	College or above	0.63(0.18-	0.64 (0.18-	2.71 (0.74-	0.52

		2.20)	2.24)	9.93)	(0.15-1.81)
Father education level	Non-educated	1	1	1	1
	Elementary	1.51 (0.47-4.82)	1.48 (0.46-4.74	1.03 (0.21-5.14)	1.00 (0.20-4.99)
	Secondary	1.60 (0.83-3.09)	1.61 (0.83-3.11	1.04 (0.58-1.84)	1.07 (0.60-1.90)
	college or above	1.42 (0.64-3.16)	1.45 (0.65-3.23	1.23 (0.61-2.46)	1.20 (0.60-2.41)
Mother job	House wife/Unemployed	1	1	1	1
	Work in private	0.79 (0.41-1.51)	0.79 (0.41-1.51)	1.40 (0.77-2.53)	1.40 (0.77-2.54)
	Employed	0.44 (0.16-1.22)	0.44 (0.16-1.21)	0.55 (0.22-1.42)	0.55 (0.21-1.44)
Father job	Unemployed/Jobless	1	1	1	1
	Work in private	1.24 (0.66-2.32)	1.24 (0.66-2.33)	0.75(0.43-1.33)	0.77(0.44-1.36)

	Employed	2.56 (0.75-8.74)	2.52 (0.73-8.70)	ns	ns

** Significant at p<0.01 * Significant at p<0.05

However, the prevalence of mental health problems was not significantly different for males by education level. Prevalence difference was insignificant across the three age groups (11-13, 14-16, and 17-19 years), maternal and paternal education level, and types of jobs or level of employment (p>0.05). The prevalence of mental health problems was twice higher among psychoactive substance users (p<0.05) compared with non-users. Differences in prevalence existed but were insignificant across these three age groups, maternal and paternal education level, and types of jobs or level of employment (p>0.05).

3.4. Correlation of mental health literacy with total difficulty scores and well-being index

The bivariate correlations showed that the mental health literacy score was negatively associated with the total and subscale strength difficulties scores (conduct problems, emotional problems, hyperactivity-inattention, and peer problems) as it is shown in **Table 4**.

Table 4: The correlations between mean mental health literacy score, strength difficulties questionnaire scale, and mental wellbeing index

Variables		Correlation coefficient (r)) with 95%CI								
		1	2	3	4	5	6	7	8	9
1.	SDQ Total difficulties	1	-.31	-.124**	.652**	.700**	.749**	617** (.570-	.825**	.863**

	problems score		9** (-.388-(-.250))	(-.198-(-.052))	(.607-.694)	(.656-.740)	(.714-.783)	.659)	(.798-.848)	(.842-.882)
2.	Mental wellbeing index	-	1	.160** (.084-.234)	-.140** (-.206-(-.076))	-.281** (-.350-(-.210))	-.293** (-.366-(-.215))	-.130** (-.200-(-.060	-.258** (-.329-(-.186))	-.279** (-.347-(-.209))
3.	MHL Total mean score	-	-	1	-.135** (-.211-(-.064))	-.136** (-.212-(-.058))	-0.006 (-.078-.058)	-.088* (-.165-(-.010))	-.166** (-.244-(-.087))	-0.051 (-.120-.016)
4.	SDQ Conduct problems	-	-	-	1	.345** (.275-.412)	.228** (.159-.301)	.294** (.220-.368)	.812** (.785-.837)	.319** (.249-.388)

	subscale mean score									
5.	SDQ Hyperactivity problems subscale mean score	-	-	-	-	1	.385** (.310-.454)	.197** (.125-.265)	.828** (.802-.854)	.380** (.310-.446)
6.	SDQ Emotional problems subscale mean score	-	-	-	-	-	1	.272** (.195-.342)	.376** (.301-.453)	.862** (.844-.880)
7.	SDQ Peer problems subscale mean score	-	-	-	-	-	-	1	.298** (.231-.362)	.721** (.683-.756)
8.	SDQ Externalizing Problems subscale mean score	-	-	-	-	-	-	-	1	.427** (.360-.489)

9.	SDQ Internalizing problems subscale mean score	-	-	-	-	-	-	-	-	1

** Correlation is significant at the 0.01 level (2-tailed); * Correlation is significant at the 0.05 level (2-tailed

4. Discussion

The Ethiopian government has taken several interventions to mitigate the adolescent mental health issue, establishing mental health facilities nationwide.[67] Despite the delayed initiation of the policy and lack of established systems and practices, there has been growing recognition of the importance of adolescent mental health promotion. The Ethiopian government has shown commitment to promoting mental health and addressing mental health issues in schools, for instance, by employing trained school counselors and establishing clubs in schools in secondary schools. The government has also trained health professionals in mental health care who are better equipped to identify and treat mental health problems in adolescents—implementing psycho-education programs in schools, public awareness campaigns, and training for community leaders and media outlets for adolescents, among others.[4] The state mechanism in promoting mental health literacy in Ethiopia is that the Ministry of Health is responsible for developing and implementing mental health policies and programs and provides training for health professionals in mental health care.[4] Stakeholders including professional associations, such as The Ethiopian Mental Health Association and The Ethiopian Psychiatric Association, are involved in policy consultation, provide training and support for health professionals, and promote mental health research in Ethiopia. These policy and programs are cascaded along the government administrative levels for actual implementation. However, there is still a paucity of evidence about the mental health status of adolescents and factors that affect mental health problems and factors that contribute for effective interventions to improve adolescents' mental health.

The SDQ is a screening tool for mental health problems in children and adolescents. It is scored on a scale of 0 to 40, with higher scores indicating more problems. The original cut-

off scores for the SDQ are 80 and 90, with scores above 90 being considered abnormal, scores between 80 and 90 being considered borderline, and scores below 80 being considered normal. [27,53] The cut-off score for the present study was slightly lower for total difficulties and subscales for hyperactivity scores than the baseline and relatively higher for internalizing scores (**Table 1**). These cut-off scores for the present study were almost similar to the baseline definition that was taken from the UK child and adolescent population during the first version of the Strengths and Difficulties Questionnaire (SDQ) development. [27,53]

Using cut-off values from local population scores ensures cultural equivalence; that supports relevancy and applicability of the instrument to the cultural groups.[68] As indicated in Table 1, the study showed that the reference scores range taken from a present study for hyperactivity and internalizing scores were higher compared to baseline reference scores ranges. [27,53] The reference scores from a present study using the original 3 band categories (cut-off scores at 80th & 90th) for conduct problems, emotional problems, peer problems, and pro-social scores were similar to the baseline reference scores ranges.[27,53] The reference scores range taken from a present study for total difficulties, hyperactivity, and externalizing scores were lower than baseline reference scores. [27,53] If the cut-off score for a given population is higher than the original cut-off score, it means that a more significant proportion of children in that population are experiencing mental health problems.[69] This could be due to factors such as poverty, social disadvantage, or exposure to trauma.[70]

It is important to note that the SDQ is a screening tool, not a diagnostic tool.[69] If a child scores above the cut-off score, it does not necessarily mean they have a mental health problem.[69] However, it does indicate that they are at an increased risk of having a problem, and further assessment is warranted. Considering these factors is essential when interpreting a lower cut-off score from the baseline. Some evidence indicated that lower cut-off values have

been more effective than the higher cut-off values at identifying child and adolescent mental health problems.[68]

These findings implied that the adolescent population in urban Ethiopia is more disadvantaged than the population on which the original cut-off scores were based. Adolescents in urban Ethiopia have been exposed to stressors like poverty, violence, or trauma. This adolescent population has less access to mental health services and is more likely to underreport mental health problems.

As shown on **Fig. 1,** the present finding was within the same range of the Ethiopian national level prevalence of adolescent mental health problems, reportedly ranging from 17-23%, like other study results in the Ethiopian context.[71–73] Prevalence of total difficulties (15.9-25.5%), internalising (14.9-28.4%), emotional (10.4-23.9%), and peer relationship (17.8-25.8%) was similar that was within the range of global prevalence(10-20%).[74]

Several studies showed discrepancies in adolescent mental health problems prevalence from region to region and country to country.[70,75–77] Similarly, these differences exist across socio-demographic characteristics. [78] From the present study, mental health problems expressed by the total strength difficulties and its subscales of problems were higher among female adolescents than male adolescents. A study among Indian children, the association between the female gender and total strength difficulties score was significant.[75] Being female was associated with emotional problems.[75] A cross-national study across 73 countries on the gender gap in adolescent mental health showed that girls have worse average mental health than boys.[79] The possible explanations include but are not limited to socio-cultural factors, gender stereotypes, trauma, and abuse that disproportionally affect girls. [22,80] In contrast, conduct and hyperactivity/inattention problems were significantly more severe among males than females.[66] Consistent with other studies, the prevalence of conduct

and externalizing problems was more significant among male adolescents than females. For example, a study in Northeast china showed a similar result.[78] A study among children and adolescents from seven European countries (Italy, Netherlands, Germany, Romania, Bulgaria, Lithuania, and Turkey) reported that externalized problems were consistently higher in males than females and reversed for internalized problems.[76] One possible explanation might be that mental health status is shaped by social, economic, and physical environments leading to inequalities that disproportionally affect gender differences and are heavily associated with risk factors for many common mental disorders.[70] According to the WHO, the relationship between the prevalence of mental health problems and poverty indices was statistically significant.[77] These poverty indices are education disparity, low income, a lack of material goods, job, and housing obstacles. For several cultural and traditional reasons, these factors impact females more than boys.[77]

Odds of mental health problems were significantly associated with some socio-demographic characteristics ($p<0.05$) differing in magnitude across these characteristics. In this study, the prevalence of mental health problems was significantly higher for females in upper elementary and lower secondary levels than for upper secondary grade levels ($p<0.05$), consistent with reports of several studies.[70,77] According to WHO report, educational status is one of the major factors determining adolescent mental health outcomes.[77]

However, the difference was insignificant for male adolescents. Furthermore, differences in prevalence have existed but little across the three age groups, maternal and paternal education level, and job types or level of employment ($p>0.05$). The present study's prevalence of mental health problems was twice higher among adolescents with a self and/or family members' experience of psychoactive substance use ($p<0.05$). On the contrary, a study in Northeast China showed that the prevalence of any mental disorders and internalizing disorders was significantly lower in younger adolescents than in elders. Children aged 11–14

years had the highest prevalence of internalizing disorders.[78] It was also inconsistent with a study in developing countries showing that lower maternal education was significantly associated with abnormal total SDQ scores.[81]

The present study revealed mental health literacy was negatively correlated with strength difficulties scores and positively correlated with mental well-being; both were significantly correlated but weaker in magnitude. These findings were consistent with existing theories and the results of previous studies. For instance, a study demonstrated that an inadequate level of mental health literacy was negatively associated with depression. The prevalence of depression was 1.52 times higher in those with levels of inadequate MHL than in those who demonstrated an adequate level of MHL.[50] Another study depicted MHL as an explanatory variable of the mental well-being of adolescents having a positive correlation with it. [82]

A possible explanation for the negative association between mental health literacy and strength difficulties scores might be that low mental health literacy contributes to the mental health promotion and prevention gap.[45,83–85] Low mental health literacy exacerbates barriers and difficulties in accessing treatment for mental health problems.[86]

Mental health literacy significantly influences adolescents' perceptions and emotional responses [87], reflecting mental health status and subjective mental well-being. Several studies revealed that mental health literacy had a significant relationship with mental health status. [88–90] A higher level of mental well-being has been reported among adolescents with better know-how about obtaining mental health services.[87] Adolescents' awareness of symptoms, causes, and mental illness treatment contributes to favorable attitudes toward seeking help.[15] Similarly, adolescents with better mental health literacy are less likely to engage in problematic health behaviours and have better help-seeking intention and self-efficacy, contributing to a better mental health and well-being.[91] Mental health literacy is

about the information, attitudes, and awareness of mental health concerns that improve self-efficacy, help-seeking intention, and health behaviour, influencing long-term health outcomes and quality of life. A lack of understanding of the symptoms and nature of mental health disorders and alternate sources of support could explain teenagers' low help-seeking intention and behaviour. [15]

Of course, help-seeking intention and self-efficacy may reflect only one of several mechanisms. Better mental health literacy leads to better mental well-being. Higher help-seeking intention and self-efficacy would only partially mediate the relation between higher mental health literacy and better mental well-being. Components of health behavior expressed in actions, targets, contexts, and time are the most determining factors of mental health outcomes.[92,93]

Mental health literacy influences mental health services and help-seeking for treatment.[94] Hence, mental health literacy is essential to improve and often but not always corresponds to help-seeking behaviours. [95] Anthony F. Jorm and his colleagues revealed that changing knowledge in principle is vital and not problematic; however, changing heartily emotional reactions to practice caring and preventing mental disorders may be much more challenging.[10]

Help-seeking, interchangeably health-seeking, has become one of the essential perspectives in understanding causes and factors for patient delay from appropriate action across different health conditions.[96] Despite the prevalent mental health problems and poor well-being, adolescents' unwillingness and low intention to seek help usually result in delays in timely treatment.[43] Most adolescents with mental health problems are reluctant and miss the use of healthcare for mental health, which increases the complexity of social, mental, and general

health outcomes.[97] The findings from this study implied that promoting mental health literacy can improve subjective mental well-being.[87]

The recently revised policy released by The Ethiopian Ministry of Health in 2017 aimed to improve adolescents' mental health by providing early identification, prevention, and treatment services. [4,21] Following the policy, programs were launched in 2018, including school-based mental health programs, community-based mental health services, and training for health care providers.[21] Empirical evidence on early identification of vulnerable groups, individuals reporting symptoms of mental health difficulties and the associated actors could enhance the impact and effectiveness of these programs implementation.[4,21] In this regard, demographic and socioeconomic sensitive approaches should be applied to mental health policies for mental health promotion and mental illness prevention.

5. Limitations

The study has some inherent limitations. It is not applicable to generalize these findings across the country; because Ethiopia exhibits multicultural and multiethnic diversity and multiple socio-demographic backgrounds. It could not even be generalized to all adolescents of the study area, because the samples were selected from the age group (11-19) and grade level (5-12), adolescents at the age of 10 years and lower grade levels were excluded during the first cycle of elementary schools during school sampling. The present study is conducted on the urban adolescents and did not address rural adolescents. The study was quantitative and lacked the qualitative aspect of how and why these findings were observed.

6. Conclusion

The cut-off scores for the present study were almost similar to the baseline definition of the first version of the Strengths and Difficulties Questionnaire (SDQ) development that was taken from the UK child and adolescent population. However, the strength difficulty cut-off score for total strength difficulty and hyperactivity scores was slightly lower and the cut-off score for internalizing scores was slightly higher. The finding was within the same range as the Ethiopian national level prevalence of adolescent mental health problems, reportedly ranging from 17-23%. The prevalence of total difficulties (15.9-25.5%), internalizing (14.9-28.4%), emotional (10.4-23.9%), and peer relationship problems (17.8-25.8%) were similar that was slightly within the range of the global prevalence(10-20%). These mental health problems were significantly greater among adolescents who had either themselves or their family members used psychoactive substances ($p \leq 0.05$) ($p < 0.05$). As depicted from strength difficulties scores, the mental health problems were significantly associated with some socio-demographic characteristics ($p < 0.05$) differing in magnitude.

Female adolescents scored higher scores on the total difficulty and its subscales (internalizing and externalizing scores) than males, implying that the prevalence of mental health problems was higher among females than their male counterparts, aligning with previous study findings. These findings highlight the need to improve adolescent mental health literacy in urban Ethiopia to improve mental health status. Gender, grade level, age, maternal and paternal education level, job types, and level of employment are factors that affect adolescents' mental health problems. Hence, promoting adolescent mental health through improving mental health literacy requires congruent approaches considering these socio-demographic factors.

The study showed a correlation between mental health literacy level, the strength difficulty scale, and mental well-being scores. It showed mental health literacy negatively correlated with strength difficulties scores and positively associated with mental well-being. These findings were consistent with existing theories and the results of previous studies. It implied that promoting mental health literacy could enhance adolescents' positive mental health, and priorities should be on vulnerable groups and individuals reporting symptoms of mental health difficulties. Future studies should involve qualitative and include the remaining determinants.

Acknowledgments

The authors would like to thank the survey respondents for their participation and contributions to the study.

Authors' contributions

Hassen, H.M and Behera M.R conceptualized the study design. Hassen, H.M collected, analyzed, and interpreted the data used in this study and was a major contributor to writing the article. Behera, D, Behera, M.R. and Dehury, R. K. assisted in analyzing and interpreting the data and writing the paper. All the authors read, commented, and approved the final article.

References

1. Renwick L, Pedley R, Johnson I, Bell V, Lovell K, Bee P, et al. Mental health literacy in children and adolescents in low- and middle-income countries: a mixed studies systematic review and narrative synthesis. Eur Child Adolesc Psychiatry

2022;(0123456789). https://doi.org/10.1007/s00787-022-01997-6
2. Sodi T, Quarshie ENB, Oppong Asante K, Radzilani-Makatu M, Makgahlela M, Nkoana S, et al. Mental health literacy of school-going adolescents in sub-Saharan Africa: a regional systematic review protocol. BMJ Open 2022;12(9):1–7.
3. Kucera M, Tomaskova H, Stodola M, Kagstrom A. A Systematic Review of Mental Health Literacy Measures for Children and Adolescents. Adolesc Res Rev 2023;8(3):339–58. A https://doi.org/10.1007/s40894-022-00202-8
4. Ministry of Health Federal Democratic Republic of Ethiopia. Ethiopian National Mental Health Strategy (2020-2025). Fed Minist Heal 2020;2025.
5. Hassen HM, Behera MR, Jena PK, Dewey RS DG. Mental Health Literacy of Adolescents and the Effect of Socio- demographic Characteristics : A Cross-sectional Study. Online J Heal allied Sci 2021;2–8. https://www.ojhas.org/issue80/2021-4-6.html
6. Claire M Kelly, Anthony F Jorm, Anne Marie Wright. Improving mental health literacy as a strategy [Internet]. Med. J. Aust. Med. J. Aust.2007;187(187):25–729.
7. Kelly CM, Jorm AF, Wright A. Improving mental health literacy as a strategy to facilitate early intervention for mental disorders. 2007;187(7):1–5.
8. Jorm AF. Why We Need the Concept of “Mental Health Literacy.” Health Commun 2015;30(12):1166–8.
9. Jorm AF, Korten AE, Jacomb PA, Christensen H, Rodgers B, Pollitt P. “Mental health literacy”: A survey of the public’s ability to recognise mental disorders and their beliefs about the effectiveness of treatment. Med J Aust 1997;166(4):182–6.
10. Jorm A. F. et al. Research on mental health literacy: What we know and what we still need to know. Aust N Z J Psychiatry [Internet] 2006;40(1):3–5.
11. Patton GC, Olsson CA, Skirbekk V, Saffery R, Wlodek ME, Azzopardi PS, et al. Adolescence and the next generation. Nature [Internet] 2018;554(7693):458–66. http://dx.doi.org/10.1038/nature25759
12. Steel Z, Marnane C, Iranpour C, Chey T, Jackson JW, Patel V, et al. The global prevalence of common mental disorders: A systematic review and meta-analysis 1980-2013. Int J Epidemiol 2014;43(2):476–93.
13. O’Sullivan J and AE. Healthy Lives: Widening The Focus On Mental Health Annual Public Health Report 2015 Camden and Islington. Camden Islingt Annu Public Heal Rep 2015;1–148.
14. David K, Park MJ, Mulye TP. The Mental Health of Adolescents: A National Profile, 2008. San Francisco: Natl. Adolesc. Health Inf. Cent. (NAHIC), Univ. Calif. 2008;
15. Eschenbeck H, Lehner L, Hofmann H, Bauer S, Becker K, Diestelkamp S, et al. The Mental Health of Adolescents: A National children and adolescents with StresSOS using online or face-to-face interventions: study protocol for a randomized controlled trial within the ProHEAD Consortium. Trials 2019;20(1):94. https://trialsjournal.biomedcentral.com/articles/10.1186/s13063-018-3157-7
16. La Maison C, Munhoz TN, Santos IS, Anselmi L, Barros FC, Matijasevich A. Prevalence and risk factors of psychiatric disorders in early adolescence: 2004 Pelotas (Brazil) birth cohort. Soc Psychiatry Psychiatr Epidemiol 2018;53(7):685–97. http://dx.doi.org/10.1007/s00127-018-1516-z
17. Mills KL. Social development in adolescence: brain and behavioural changes. Univ. Coll. London2015
18. Manganello JA. Health literacy and adolescents: A framework and agenda for future research. Health Educ Res 2008;23(5):840–7.
19. Shahraki-Mohammadi A, Panahi S, Ashouri A, Sayarifard A. Important Systemic Factors for Improving Adolescent Mental Health Literacy. Iran J Psychiatry

2023;18(1):45–54.
20. Seboka BT, Hailegebreal S, Negash M, Mamo TT, Ewune HA, Gilano G, et al. Predictors of Mental Health Literacy and Information Seeking Behavior Toward Mental Health Among University Students in Resource-Limited Settings. Int J Gen Med 2022;15:8159–72.
21. Hanlon C, Alem A, Lund C, Hailemariam D, Assefa E, Giorgis TW, et al. Moving towards universal health coverage for mental disorders in Ethiopia. Int J Ment Health Syst [Internet] 2019;13(1):1–17. https://doi.org/10.1186/s13033-019-0268-9
22. Eaton NR, Keyes KM, Krueger RF, Balsis S, Skodol AE, Markon KE, et al. An invariant dimensional liability model of gender differences in mental disorder prevalence: evidence from a national sample. J Abnorm Psychol 2012;121(1):282–8.
23. Fok MSMM, Wong TKSS. What does health literacy mean to children? Contemp nurse a J Aust Nurs Prof 2002;13(2–3):249–58.
24. Goodman R, Ford T, Simmons H, Gatward R, Meltzer H. Using the Strengths and Difficulties Questionnaire (SDQ) to screen for child psychiatric disorders in a community sample. Int Rev Psychiatry 2003;15(1–2):166–72.
25. Skinner D, Sharp C, Marais L, Serekoane M, Lenka M. Assessing the Value of and Contextual and Cultural Acceptability of the Strength and Difficulties Questionnaire (SDQ) in Evaluating Mental Health Problems in HIV/AIDS Affected Children. Int J Ment Health 2014;43(4):76–89.
26. Chongwo E, Ssewanyana D, Nasambu C, Mwangala PN, Mwangi PM, Nyongesa MK, et al. Validation of a Swahili version of the World Health Organization 5-item well-being index among adults living with HIV and epilepsy in rural coastal Kenya. Glob Heal Res Policy 2018;3(1):3–9.
27. Goodman R. The strengths and difficulties questionnaire: A research note. J Child Psychol Psychiatry Allied Discip 1997;38(5):581–6.
28. Atilola O, Singh Balhara YP, Stevanovic D, Avicenna M, Kandemir H. Self-Reported Mental Health Problems Among Adolescents in Developing Countries. J Dev Behav Pediatr2013;34(2):129–37.
29. Goodman A, Lamping DL, Ploubidis GB. When to use broader internalising and externalising subscales instead of the hypothesised five subscales on the strengths and difficulties questionnaire (SDQ): Data from british parents, teachers and children. J Abnorm Child Psychol 2010;38(8):1179–91.
30. Downs A, Boucher LA, Campbell DG, Polyakov A. Using the WHO–5 well-being index to identify college students at risk for mental health problems. J Coll Stud Dev 2017;58(1):113–7.
31. De Wit M, Pouwer F, Gemke RJBJ, Delemarre-Van De Waal HA, Snoek FJ. Validation of the WHO-5 well-being index in adolescents with type 1 diabetes. Diabetes Care 2007;30(8):2003–6.
32. Kusier AO, Folker AP. The Well-Being Index WHO-5: Hedonistic foundation and practical limitations. Med Humanit 2019;1–7.
33. Garland AF, Deyessa N, Desta M, Alem A, Zerihun T, Hall KG, et al. Use of the WHO's perceived Well-Being Index (WHO-5) as an efficient and potentially valid screen for depression in a low income country. Fam Syst Heal 2018;36(2):148–58.
34. Topp CW, Østergaard SD, Søndergaard S, Bech P. 1. The WHO-5 well-being index: A systematic review of the literature. Psychother Psychosom 2015;84(3):167–76.
35. Hoosen N, Davids EL, Vries PJ De, King MS. The Strengths and Difficulties Questionnaire (SDQ) in Africa : a scoping review of its application and validation. Child Adolesc Psychiatry Ment Health [Internet] 2018;1–39. 36. Hassen HM, Behera MR, Jena PK, Dewey RS. Effectiveness and Implementation Outcome

Measures of Mental Health Curriculum Intervention Using Social Media to Improve the Mental Health Literacy of Adolescents. J Multidiscip Healthc 2022;15:979–97. 37. Maher MS and BA. Philosophy and Psychopathology. Springer-Verlag New York Inc.: Springer-Verlag New York Heidelberg Berlin London Paris Tokyo Hong Kong; 1990.

38. Brown TA, Barlow DH. Dimensional Versus Categorical Classification of Mental Disorders in the Fifth Edition of the Diagnostic and Statistical Manual of Mental Disorders and Beyond : Comment on the Special Section. 2005;114(4):551–6.
39. Tusaie KR. Epidemiology of child psychiatric disorders in Addis Ababa, Ethiopia. Division of Child and Adolescent Psychiatry Department of Clinical Sciences; 2013.
40. Ling J, Tay YF, Klainin-Yobas P. Mental health literacy levels. Arch Psychiatr Nurs 2018;
41. World Health Organization. Adolescents: health risks and solutions: https://www.who.int/news-room/fact-sheets/detail/adolescents-health-risks-and-solutions. 2018;
42. Polanczyk G V., Salum GA, Sugaya LS, Caye A, Rohde LA. Annual research review: A meta-analysis of the worldwide prevalence of mental disorders in children and adolescents. J Child Psychol Psychiatry Allied Discip 2015;56(3):345–65.
43. Divin N, Harper P, Curran E, Corry D, Leavey G. Help-Seeking Measures and Their Use in Adolescents: A Systematic Review. Adolesc Res Rev2018;3(1):113–22. http://dx.doi.org/10.1007/s40894-017-0078-8
44. Atilola O. Level of community mental health literacy in sub-Saharan Africa: Current studies are limited in number, scope, spread, and cognizance of cultural nuances. Nord J Psychiatry 2015;69(2):93–101.
45. Cortina M, Sodha A, Fazel M, Ramchandani PG. Prevalence of Child Mental Health Problems in Sub-Saharan Africa _ Global Health _ JAMA Pediatrics _ The JAMA Network. Arch Pediatr Adolesc Med 2012;166(3):276–81.
46. Zeleke WA, Nichols LM, Wondie Y. Mental Health in Ethiopia: an Exploratory Study of Counseling Alignment with Culture. Int J Adv Couns 2019;41(2):214–29.
47. CSA and UNICEF Ethiopia. Multidimensional Child Deprivation in Ethiopia. 2018; www.unicef.org/Ethiopia and http://www.csa.gov.et
48. Jorm AF. Mental health literacy. Public knowledge and beliefs about mental disorders - University College London. Br J psychiatry J Ment Sci 2000;177:396–401. 49. Johnson RL. Pathways to adolescent health: Early intervention. J Adolesc Heal 2002;31(6 SUPPL.):240–50. 50. Lam LT. Mental health literacy and mental health status in adolescents: A population-based survey. Child Adolesc Psychiatry Ment Health 2014;8(1):1–8.
51. Hassen HM, Behera MR, Jena PK, Satpathy SK. Validity and Reliability of the Amharic Version of the HLS-EU-Q47 Survey Questionnaire among Urban School Adolescents and University students in Dire Dawa, Ethiopia. Prepr Res Sq 2020;
52. Kersten P, Czuba K, McPherson K, Dudley M, Elder H, Tauroa R, et al. A systematic review of evidence for the psychometric properties of the Strengths and Difficulties Questionnaire. Int J Behav Dev 2016;40(1):64–75.
53. Mathai J, Anderson P, Bourne A. The Strengths and Difficulties Questionnaire (SDQ) as a screening measure prior to admission to a Child and Adolescent Mental Health Service (CAMHS). Aust e-Journal Adv Ment Heal 2002;1(3):235–46.
54. Heizomi H, Kouzekanani K, Jafarabadi MA, Allahverdipour H. Psychometric properties of the persian version of mental health literacy scale. Int J Womens Health 2020;12:513–20.
55. O’Connor M, Casey L, Clough B. Measuring mental health literacy-a review of scale-

based measures. J Ment Heal 2014;23(4):197–204.
56. Campos L, Dias P, Palha F, Duarte A, Veiga E. Development and psychometric properties of a new questionnaire for assessing mental health literacy in young people. Univ Psychol 2016;15(2):61–72.
57. Bale J, Grové C, Costello S. I. Building a mental health literacy model and verbal scale for children: results of a Delphi study Ment Heal Prev 2018;12:26–35. 58. Lam LT. Mental health literacy and mental health status in adolescents: a population-based survey. Child Adolesc Psychiatry Ment Health 2014;8(26):1–8.
59. Dias P, Campos L, Almeida H, Palha F. Mental health literacy in young adults: Adaptation and psychometric properties of the mental health literacy questionnaire. Int J Environ Res Public Health 2018;15(7).
60. Wei Y, McGrath PJ, Hayden J, Kutcher S. Mental health literacy measures evaluating knowledge, attitudes and help-seeking: A scoping review. BMC Psychiatry [Internet] 2015;15(1).
61. Tomé G, Gaspar de Matos M, Camacho I, Gomes P, Reis M, Branquinho C. Mental health promotion in school context – Validation of the ES ' COOL scale for teachers. J Psychiatry Behav Sci 2018;2:(1009). 62. Bjørnsen HN, Eilertsen MEB, Ringdal R, Espnes GA, Moksnes UK. Positive mental health literacy: Development and validation of a measure among Norwegian adolescents. BMC Public Health 2017;17(1):1–10.
63. Reilly MO, Svirydzenka N, Adams S, Dogra N. Review of mental health promotion interventions in schools. Soc Psychiatry Psychiatr Epidemiol 2018;53(7):647–62.
64. Hoofs H, Jansen NWH, Mohren DCL, Jansen MWJ, Kant IJ. The context dependency of the self-report version of the Strength and Difficulties Questionnaire (SDQ): A cross-sectional study between two administration settings. PLoS One 2015;10(4):1–21.
65. Koskelainen M, Sourander A, Kaljonen A. The Strengths and Difficulties Questionnaire among Finnish school-aged children and adolescents. Eur Child Adolesc Psychiatry 2000;9(4):277–84.
66. De Vries PJ, Davids EL, Mathews C, Aarø LE. Measuring adolescent mental health around the globe: Psychometric properties of the self-report Strengths and Difficulties Questionnaire in South Africa, and comparison with UK, Australian and Chinese data. Epidemiol Psychiatr Sci 2018;27(4):369–80.
67. Hughes TL, Quinn C, Tiberi A, Zeleke WA. Developing a Framework to Increase Access to Mental Health Services for Children With Special Needs in Ethiopia. Front Sociol 2020;5:1–6.
68. Renshaw TL. Screening Using the SDQ Total Difficulties Scale: An Analog Test of Three Cutoff Values. J Psychoeduc Assess 2019;37(8):1030–6.
69. Editor S, Kamphaus RW. Contemporary Issues in Psychological Assessment. Springer Berlin Heidelberg; 2015.
70. Carod-Artal FJ. Social determinants of mental health. Glob Ment Heal Prev Promot 2017;(2):33–46.
71. FDREMH. Federal Democratic Republic of Ethiopia Ministry of Health: National Mental Health Strategy (2012/13- 2015/16). Heal Sect Dev Program IV 2010/11 – 2014/15 2011; 2010:1–114.
72. Lu W. Child and Adolescent Mental Disorders and Health Care Disparities: Results from the National Survey of Children's Health, 2011– 2012. 2017;28:988–1011.
73. Dachew BA, Bifftu BB, Tiruneh BT, Anlay DZ, Wassie MA, Betts K. Prevalence and determinants of mental distress among university students in Ethiopia : a systematic review protocol. 2019;8–11.
74. Baker-henningham H, Belfer M, Conti G, Ertem I, Omigbodun O, Rohde LA, et al.

Global Mental Health 2 Child and adolescent mental health worldwide : evidence. Lancet 2011;378(9801):1515–25. 75. Nair S, Ganjiwale J, Kharod N, Varma J, Nimbalkar SM. Epidemiological survey of mental health in adolescent school children of Gujarat, India. BMJ Paediatr Open 2017;1(1):e000139.

76. Kovess V, Carta MG, Pez O, Bitfoi A, Koc C, Goelitz D, et al. The School Children Mental Health in Europe (SCMHE) Project: Design and First Results. Clin Pract Epidemiol Ment Heal 2015;11(1):113–23.

77. Patel V, Kleinman A. Poverty and common mental disorders in developing countries. Bull World Health Organ 2003;81(8):609–15. 78. Lijuan T, Chao J, Wenming X, Huijuan M, Lianzheng Y, Ming L, et al. Prevalence of Psychiatric Disorders among Children and Adolescents in Northeast China. PLoS One 2014;9(10):e111223.

79. Campbell OLK, Bann D, Patalay P. SSM - Population Health The gender gap in adolescent mental health : A cross-national investigation of 566 , 829 adolescents across 73 countries. SSM - Popul Heal [Internet] 2021;132020). 80. Tedstone Doherty D, Kartalova-O’Doherty Y. Gender and self-reported mental health problems: Predictors of help seeking from a general practitioner. Br J Health Psychol 2010;15(1):213–28.

81. Atilola O, Pal Y, Balhara S, Avicenna M. Self-Reported Mental Health Problems Among Adolescents in Developing Countries : Results from an International Pilot. J Dev Behav Pediatr 2013;34(2):129–37.

82. Bjørnsen HN, Espnes GA, Eilertsen MEBEB, Ringdal R, Moksnes UK. The Relationship Between Positive Mental Health Literacy and Mental Well-Being Among Adolescents: Implications for School Health Services. J Sch Nurs 2019;35(2):107–16. 83. World Health Organization. WHO Adolescent mental health 2018; http://www.who.int/news-room/fact-sheets/detail/adolescent-mental-health

84. Belfer ML. Child and adolescent mental disorders: The magnitude of the problem across the globe. J Child Psychol Psychiatry Allied Discip 2008;49(3):226–36.

85. Atilola O, Pal Y, Balhara S, Avicenna M, Singh Balhara YP, Stevanovic D, et al. Self-Reported Mental Health Problems Among Adolescents in Developing Countries. J Dev Behav Pediatr [Internet] 2013;34(2):129–37. 86. Ibrahim N, Mohd Safien A, Siau CS, Shahar S. The Effectiveness of a Depression Literacy Program on Stigma and Mental Help-Seeking Among Adolescents in Malaysia: A Control Group Study With 3-Month Follow-Up. Inq (United States) 2020;57.

87. Bjørnsen HN, Espnes GA, Eilertsen MEB, Ringdal R, Moksnes UK. The Relationship Between Positive Mental Health Literacy and Mental Well-Being Among Adolescents: Implications for School Health Services. J Sch Nurs 2019;35(2):107–16.

88. Lam LT. Mental health literacy and mental health status in adolescents: a population-based survey. Child Adolesc Psychiatry Ment Health 2014;8(26):126–9. 89. Wu C, Liu S, Chang S. Surveys of medical seeking preference , mental health literacy , and attitudes toward mental illness in Taiwan , 1990 e 2000. J Formos Med Assoc [2014;113(1):33–41. 90. Furnham A, Lee V, Kolzeev V. Mental health literacy and borderline personality disorder (BPD): what do the public “make” of those with BPD? Soc Psychiatry Psychiatr Epidemiol 2015;50(2):317–24.

91. Oldfield J, Stevenson A, Ortiz E, Haley B. Promoting or suppressing resilience to mental health outcomes in at risk young people: The role of parental and peer attachment and school connectedness. J Adolesc 2018;64: 2017):13–22.

92. Presseau J, Mccleary N, Lorencatto F, Patey AM, Grimshaw JM, Francis JJ. Action , actor , context , target , time (AACTT): a framework for specifying behaviour. 2019;1–13.

93. Kan MPH, Fabrigar LR, Fishbein M. Theory of Planned Behavior. 2017;1–8.

94. Kessler RC, M. A, Anthony JC, De Graaf R, Demyttenaere K, Gasque I, et al. Lifetime prevalence and age-of-onset distributions of mental disorders in the World Health Organization' s. World Psychiatry 2007;6:168-176) 2007;6:168–76.
95. Wei Y, Mcgrath PJ, Hayden J, Kutcher S, Wei Y, Mcgrath PJ, et al. Measurement properties of mental health literacy tools measuring help-seeking : a systematic review. J Ment Heal 2017;0(0):000. 96. Cornally N, Mccarthy G. Help-seeking behaviour: A concept analysis. Int J Nurs Pract 2011;17(3):280–8.
97. Bonabi H, Müller M, Ajdacic-Gross V, Eisele J, Rodgers S, Seifritz E, et al. Mental health literacy, attitudes to help seeking, and perceived need as predictors of mental health service use: A longitudinal study. J Nerv Ment Dis 2016;204(4):321–4.

Click here to access/download
Supporting Information
information_sheet_and_consent_formpdf.pdf

Ethical certificate

Click here to access/download
Supporting Information
IRB_Approval_Certificate_.pdf

Click here to access/download
Supporting Information
Ethical Approval KIIT and Haramaya Universities Merged.pdf

Original Research

Mental health issues and the association of mental health literacy among adolescents in urban Ethiopia

Hailemariam Mamo Hassen[1], Manas Ranjan Behera[2*], Deepanajli Behera[2], Ranjit Kumar Dehury[3]

[1]Department of Biological Sciences, Dire Dawa University, Dire Dawa, Ethiopia

[2] School of Public Health, KIIT Deemed to be University, Bhubaneswar, India

[3]School of Management Studies, University of Hyderabad, Telangana, India

* manas.beherafph@kiit.ac.in

Abstract

Background

Epidemiological evidence about the prevalence of adolescent mental health issues and their association with mental health literacy is crucial for sustained mental health promotion strategies. Adolescence is a critical life stage for mental health promotion. However, evidence is not available among Ethiopian school adolescents. Hence, the present study examined the prevalence of adolescents' mental health issues and their correlation with mental health literacy.

Materials and methods

Formatted: Font: (Default) Cambria, 18 pt, Bold, Font color: Auto, (Asian) Chinese (PRC)

Formatted: Font: 18 pt, Font color: Auto

A cross-sectional study was conducted among adolescents (grades 5-12) ~~in Dire Dawa, Ethiopia~~ in Dire Dawa city, Eastern Ethiopia using multistage random sampling. Data was collected using the Strength and Difficulty Questionnaire, WHO-5 well-being index, and mental health literacy questionnaire**.** SPSS version 25 was used for the descriptive, Chi-square, binary logistic regression, and correlation analyses.

Formatted: Font color: Auto

Formatted: Space After: 10 pt

Results

Formatted: Font: (Default) Cambria, 18 pt, Bold, Font color: Auto, (Asian) Chinese (PRC)

Formatted: Font: 18 pt, Font color: Auto

About 15.9-28.4% of adolescents had reported mental health problems: internalizing problems (14.9-28.4%), emotional problems (10.4-63.9%), and peer relationship problems (17.8-25.8%). These mental health problems were significantly greater among adolescents who had either themselves or their family members used psychoactive substances ($p \leq 0.05$). Females from upper elementary (5-8 grade) and lower secondary (9-10) grade levels had a higher prevalence of mental health problems (AOR: 2.60 (0.95-7.10, $p<0.05$)). The effect of age, parental education, or employment status was insignificant ($p>0.05$). The prevalence of depression ranged from 18.0-25.5%. Mental health literacy was negatively correlated with total difficulties scores and positively associated with mental well-being scores ($p<0.05$).

Formatted: Font color: Auto

Formatted: Space After: 10 pt

Conclusion

The prevalence of adolescents' mental health problems was higher. It implied that promoting mental health literacy could enhance adolescents' positive mental health. Intervention programs should prioritize vulnerable groups and individuals reporting symptoms of mental health difficulties. Future studies should involve qualitative studies and consider effect of other determinants.

Formatted: Font color: Auto

Formatted: Justified

1. Introduction

Globally, mental health problems disproportionately affect adolescents in low-income countries and they remain the leading causes of disease burden among children and young people.[1] Knowing about these mental health conditions, their symptoms, and treatments termed as mental health literacy is essential for everyone but is crucial for adolescents.[2] Mental health literacy is defined as understanding and applying information about mental health. [2] In this regard, much has been known about the mental health literacy of adolescents from high-income countries.[3] However, it is still a relatively new concept in Africa.[2] Additionally, studies revealed that access to mental health information or services in Africa is lower due to poverty, lack of education, and cultural stigma around mental illness.[1] Nevertheless, limited evidence in Africa, including Ethiopia, has indicated a growing awareness about the importance of mental health literacy. Despite the challenges associated with poverty, cultural stigma, lack of access to health facilities, and lower education coverage, countries are working to improve mental health literacy.[2]

The Ethiopian Ministry of Health has developed a mental health policy focusing on primary mental healthcare and promoting positive mental health, including a mental health awareness campaign[4]. The present study area is Dire Dawa, one of Ethiopian provinces known for its ethnic and cultural language diversity. A recent study revealed that adolescent mental health literacy from Dire Dawa city was lower, consistent with similar studies reports and showing variability across socio-demographic factors. [5]

Epidemiological evidence about the prevalence of mental health problems and their association with mental health literacy is crucial for sustained mental health promotion strategies, as adolescence is a critical life stage for mental health promotion.[6–10] However, such evidence is rarely available among Ethiopian school adolescents. Adolescent mental

Formatted: Font: (Default) Cambria, 22 pt, Bold, (Asian) Chinese (PRC)

Formatted: Font color: Auto

Formatted: Font color: Auto

Formatted: Font color: Auto

Formatted: Font color: Auto

Formatted: Font color: Auto

Formatted: Font color: Auto

Field Code Changed

Formatted: Justified

health has piqued the interest of public health professionals due to the unique nature of adolescence.[11] Therefore, adolescence remains a critical life stage for mental health promotion.[11] A popular saying goes, “there is no health without mental health.” [12] With multifaceted burdens, mental health is integrally linked to physical health and well-being.[13] The typical adolescent mental health problems include but are not limited to: substance use/abuse or disorders, depression/mood disorders, anxiety, schizophrenia, stress/neurosis, behavioral disorders, postpartum psychosis, and posttraumatic stress disorder (PTSD). [14,15] Several studies showed that these mental health problems in adulthood occur during adolescence. [14,15]

For multiple reasons, adolescents are among the most important groups for mental health studies. For example, adolescence is key to physical, emotional, and cognitive progress.[11,16,17] Fundamentally, mental, physical, and emotional developmental processes evolve during adolescence. [18] Also public health perspectives emphasizing mental health information during early childhood,[18] would help in later life in managing and preventing mental health problems.[18]

Mental health literacy is essential to preventing mental illnesses, consisting of knowledge about mental health problems and recognizing illnesses with their symptoms.[1,3] Studying the mental health status and its association with the mental health literacy of adolescents is paramount because it helps understand these variables across varied cultural settings.[1] The peak of the onset of mental disorders mainly occurs in the adolescent period and needs urgent attention. [19] In low-resource countries like Ethiopia, risk factors such as cigarette smoking, alcohol consumptions, early co-habitation, unhealthy lifestyles etc are higher among adolescents.[20,21] leading to various mental health problems.[11,16,17] Hence,

Formatted: Font color: Auto
Formatted: Font color: Auto
Formatted: Font color: Auto
Formatted: Font color: Auto
Formatted: Font color: Auto
Formatted: Font color: Auto
Formatted: Space After: 10 pt
Formatted: Font color: Auto
Formatted: Font color: Auto
Formatted: Font color: Auto
Formatted: Font color: Auto
Field Code Changed
Field Code Changed
Formatted: Font color: Auto
Formatted: Font color: Auto
Formatted: Justified

epidemiological methods like cross-sectional studies provides empirical evidence about the mental health status and factors that contribute to mental health problems.[22] Early understanding of these evidence contribute for making informed and effective interventions to improve adolescents' mental health. [23]

As a result, there is an increasing demand for adolescents' mental health and well-being outcome measures to inform the public and policymakers regarding individual or service-level health promotion and therapeutic practices. In this regard, the strength difficulties questionnaire (SDQ) and the mental well-being index (WHO-5) have been essential to meet the criteria of valid and reliable measures.

The Strengths and Difficulties Questionnaire (SDQ) and the WHO-5 Well-Being Index are commonly used to assess adolescents' mental health.[24–34] The SDQ is a 25-item self-report questionnaire that assesses five mental health domains: conduct problems, emotional symptoms, peer problems, hyperactivity-inattention, and pro-social behavior. [24,25,27–29] The WHO-5 is a 5-item self-report questionnaire that assesses overall well-being. [26,30–34] Both instruments are good choices for assessing adolescent mental health in low-resource settings because they are brief, relatively inexpensive, and easy to administer, and shown to be reliable and valid in adolescents from low-resource settings have been validated in a variety of settings and translated into multiple languages for low-resource settings including in Ethiopia[35,36] Therefore, the SDQ and the WHO-5 are good choices for assessing adolescent mental health in low-resource settings. In this article, the concept of "normalcy" and "mentally sick" as two separate spheres was considered by taking the categorical approach than the dimensional approach of measuring mental health status. [37,38]

The assessment of mental health issues using the familiar Diagnostic Standard Manual – 5 (DSM-5) and International Classification of Disease (ICD-11) analysis has been published in

previous studies.[37,38] Such an approach is marked by clustering disorders, namely, externalizing and internalizing problems. Instruments with high validity are routinely used to measure the internalizing groups (i.e., depressive, somatic, and anxiety symptoms) and the externalizing groups (substance use, disruptive, and conduct symptoms). Furthermore, the well-being state was examined using the WHO-5 index, with lower scores indicating depression.

The daily suffering of people with mental illness affects family members, mainly children, and adolescent populations.[39] Globally, about 25% of the total population and 10-20% of children and adolescents have mental disorders, of which half of all these mental health problems begin by the age of fourteen and three-fourths by the age of early twenties.[40,41] A worldwide-pooled prevalence meta-analysis on mental disorders from all regions of the world addressing 27 countries showed a prevalence of 13.4% ranging from 11.3 to 15.9 (CI: 95%), which indicates mental disorders is affecting a large proportion of the child and adolescent population.[42] According to studies that used the strengths and difficulties questionnaire (SDQ), around 10.5% (5.8-15%) of adolescents in developing countries had mental health concerns.[28]

The prevalence of mental health problems among adolescents in Africa including Ethiopia, was reportedly higher.[43–47] For instance, a meta-analysis study on adolescents in sub-Saharan Africa, including Ethiopia (N=9713), showed the proportion of adolescents having psychopathology was 14.3% (13.6%-15.0%), and the prevalence of psychological disorders was 19.8% (18.8%-20.7%).[45]

These mental health problems are exacerbated by modifiable factors such as low mental health service seeking, lower mental health service use, stigma, discrimination, and common human rights abuses. These factors are linked to immediate and intermediate mental health

Formatted: Justified

outcomes such as health literacy and behaviour. Mental health literacy is a changeable factor linked to improved psychological outcomes.[6–10] It refers to how well a person understands mental diseases and the associated factors.[6–10] Mental health literacy includes an individual's potential to identify mental distress.[9,48,49] Some qualitative descriptions revealed that mental health difficulties and mental well-being is associated with mental health literacy, despite the lack of quantitative evidence of the correlation between mental health literacy, mental health status, and mental well-being.[30]

There has been a scarcity of available epidemiological evidence about mental health conditions and literacy among Ethiopian school adolescents. There was also a lack of evidence about school adolescents' mental health status and the effect of socio-demographic characteristics in the existing literature. Therefore, the objectives of the study were:

Formatted: Space After: 10 pt

- To assess the prevalence of mental health issues among urban adolescents in Ethiopia
- To evaluate the relationship between mental health literacy and mental health issues and
- To examine the impact of socio-demographic characteristics on mental health issues

2. Materials and methods

2.1. Study design and sampling

The present research was a cross-sectional study among public and private school adolescents in grades 5 through 12 from 11 to 19 years in Dire Dawa, Ethiopia. A multistage random sampling method was used to choose the respondents. The prevalence of self-reported mental health difficulty was obtained from the pilot study of the same population (p=0.378) [51], and the margin of sampling error (d=0.04) was taken to calculate the sample size.

$$n \geq \frac{(Za/2)^2 \text{ x } P(1-P)}{d^2} = \frac{1.96^2 \text{ x } 0.378(1-0.378)}{0.04^2} = 559.673 \approx 560$$

Formatted: Font color: Auto
Formatted: Font color: Auto
Formatted: Font color: Auto
Formatted: Font color: Auto
Formatted: Font color: Auto
Formatted: Font color: Auto
Formatted: Font color: Auto
Formatted: Font color: Auto
Formatted: Font color: Auto
Formatted: Font color: Auto
Formatted: Font color: Auto
Formatted: Font color: Auto
Formatted: Font color: Auto
Formatted: Font color: Auto
Formatted: Font color: Auto
Formatted: Justified

Data was collected from June 2020 to July 2020 using the mental health literacy questionnaire (MHLQ), the Strength and Difficulty Questionnaire (SDQ), and the WHO-5 well-being index after cross-cultural validation in the study settings. Maximum sample size estimation (n= 924) was approached after taking design effect (d=1.5) and a 10% non-response. About 751 participants filled out the questionnaires out of the 924 potential participants.

2.2. Data collection tools

Formatted: Font: (Default) Cambria, (Asian) Chinese (PRC)

Formatted: Font color: Auto

Data collection tools were the Strengths and Difficulties Questionnaire (SDQ), mental health literacy questionnaire (MHLQ), and mental well-being index (WHO-5). The Strengths and Difficulties Questionnaire (SDQ) is a valid, rapid measure for emotional and behavioral problems worldwide.[24,25,27–29] SDQ is a 25-item, 3-point Likert scale (0=not true, 1=somewhat true, and 2=certainly true). It measures subscale difficulty problems that are emotional(SDQ3,8,13,16&24), conduct (SDQ5,7,12,18&22), hyperactivity/inattention(SDQ 2,10,15,21&25), peer(SDQ6,11,14,19&23) and pro-social behaviour (SDQ 1,4,9,17&20) problems.[52] The total difficulties score combines conduct, hyperactivity, emotional, and peer relationship behaviour problems. The externalizing problem manifests emotional and peer behavioural relationship problems. Meanwhile, the internalizing problem manifests the conduct and hyperactivity/inattention problems.

Adding up the first four subscales leads to total difficulty scores (the higher the total difficulties score, the more significant the mental health difficulty).[52] The fifth sub-scale of the SDQ reflects pro-social behaviour (the higher the score, the better the pro-social behaviour).[52] SDQ is widely used in resource-poor countries to measure children and adolescents' mental and emotional problems. The baseline for cut-off scores definition of total and subscales strength and difficulty scores were from the United Kingdom population

Formatted: Justified

norms defined during the instrument development. [27,53] The self-administered SDQ is a validated and widely used measurement tool for determining mental health problems in children and adolescents.[24,25,27–29] It has already been translated into over 60 languages, including Ethiopian Amharic.[24,25,27–29] It has reportedly been an effective tool for assessing children's mental health in a recent scoping review in Africa. [35]

Mental health literacy was measured using the mental health literacy questionnaire (MHLQ). This MHLQ tool is freely available and validated for adults, [54] and adolescents [7,55–63] living in low-income countries to measure health literacy scores. In the context of the current investigation, its reliability was evaluated using Cronbach's alpha and found to be 0.834. MHLQ has 33 items with five Likert scale ratings *(1=strongly disagree, 2=slightly disagree, 3=neither agree nor disagree, 4=slightly agree, 5=strongly agree).* The MHLQ consists of 33 questions that measure factors like recognition (10 questions), knowledge (8 questions), attitudes (8 questions), and beliefs (7 questions) of the adolescents on mental health status. [6–10] The range of scores for the mental health literacy tool is 33 to 165. A higher score implies a better level of mental health literacy.

The mental well-being index (WHO-5) is an overarching expression of the quality of the various domains in the life of adolescents subjective to their mental and psychological well-being. Mental well-being was assessed using the WHO's well-being index, which consists of 5 items (WHO-5). [26,30–34] It is a Likert-type scale with a five-point scale (5=all of the time, 4=most of the time, 3=more than half the time, 2=less than half the time, 1= some of the time, 0=at no time).

2.3. Ethical approval

Ethical approval was obtained from KIIT University (Reference: KIIT/KIMS /IEC/63/2019) and Haramaya University (Reference: 00. HMS/10.0/3763/2020. Written informed consent

Formatted: Font color: Auto

Formatted: Justified

was obtained from all participants. As a duly authorized representative, written informed consent was obtained from school principals.

2.4. Statistical analysis

SPSS version 25 was used to carry out the statistical analysis. Descriptive analysis was done to present the socio-demographic characteristics, cut-off score determination, and prevalence of mental health problems. Chi-square test and binary logistic regression analysis were used to estimate the difference and compare the mental health problems prevalence between males and females across some socio-demographic characteristics. Correlation analysis was performed to assess the associations between mental health literacy, strength difficulties questionnaire scale, and subjective mental well-being. Estimates used a confidence interval of 95 % ($p \leq 0.05$).

The cut-off score for mental health problems(cases) was defined using the original 3 band categorizations (cut-off scores at 80th & 90th).[64–66] Scores of every item in the scale are added from 0–10. The added scores (range of 0–40) were calculated by adding scores on the emotion components, conduct problems, hyperactivity, inattention, and peer problem scales.[27–29] The cut-off scores was defined by percentile into the highest first 10% (abnormal), the next 10% (borderline), and the remaining 80% (normal). The cut-off for mental health problems (caseness) was the highest at first 10 %.

Formatted: Font: (Default) Cambria, (Asian) Chinese (PRC)

Formatted: Font color: Auto

Formatted: Font: (Default) Times New Roman, 12 pt, Not Bold, Font color: Auto

Formatted: Font color: Auto

Formatted: Justified

3. Results

3.1. The socio-demographic characteristics of study participants

About 751 participants comprising 50.1% males and 49.9% females, completed the questionnaires, and statistical analysis was performed for the 731 participants after removing 2.66% of respondents from the analysis for significant missing data. It did not affect the sample power because a greater number of participants were participated compared to the calculated minimum sample size (n=565). The mean age with standard deviation of male and female respondents was 16.27 ± 2.19 and 15.95 ± 2.02, respectively.

3.2. Strength difficulty scores and prevalence of mental health status

The original 3 bands (cut-off scores at 80th & 90th) and the newer 4 band (cut-off scores at 80th, 90th & 95th) categorization were reported just for comparison as reported in **Table 1**. The original 3 band was used to determine and estimate adolescents' mental health issues. The mental health problems expressed with the total difficulties and in subscales, in general, were higher for female adolescents than male adolescents. The prevalence of mental health problems (cases) was presented across gender and age categories.

Table 1: The cut of scores for baseline reference ranges (caseness) for both the original 3 band and the newer 4 band categorizations

Self-	Original 3 band categories(cut-off scores at 80th & 90th)	Newer 4 band categorisations (cut-off scores at 80th, 90th & 95th)

Formatted: Font: (Default) Cambria, Bold, (Asian) Chinese (PRC)

Formatted: Font color: Auto

Formatted: Font color: Auto

Formatted: Font: 12 pt, Font color: Auto, (Asian) Chinese (PRC)

Formatted: Font: 11 pt, Font color: Auto

Formatted: Font color: Auto

Formatted: Font color: Auto

Formatted: Justified

completed SDQ	Normal		Borderline		Abnormal		Close to average		Slightly raised slightly lowered		High (/low)		Very high (/very low	
	Baseline norm	Present study range	Baseline norm	Present study range	Baseline norm	Present study range	Baseline norm	Present study range	Baseline norm	Present study range	Baseline norm	Present study range	Baseline norm	Present study range
Total difficulties score	0-15	0-14	16-19	15-17	20-40	18-40	0-14	0-14	15-17	15-17	18-19	18-20	20-40	20-40
Emotional problems score	0-5	0-5	6	6	7-10	7-10	0-4	0-5	5	6	6	7	7-10	8-10
Conduct problems score	0-3	0-3	4	4	5-10	5-10	0-3	0-3	4	4	5	5	6-10	6-10
Hyperact	0-5	0-4	6	5	7-	6-	0-5	0-4	6	5	7	6	8-	7-9

Formatted: Font color: Auto
Formatted: Font color: Auto
Formatted: Font color: Auto
Formatted: Font color: Auto
Formatted: Font: 11 pt, Font color: Auto
Formatted: Font: 11 pt, Font color: Auto
Formatted: Font: 11 pt, Font color: Auto
Formatted: Font: 11 pt, Font color: Auto
Formatted: Font color: Auto
Formatted: Font color: Auto
Formatted: Font color: Auto
Formatted: Justified, Space After: 0 pt
Formatted: Font: 11 pt, Font color: Auto
Formatted: Font color: Auto
Formatted: Font color: Auto
Formatted: Justified, Space After: 0 pt
Formatted: Font: 11 pt, Font color: Auto
Formatted: Font color: Auto
Formatted: Font color: Auto
Formatted: Justified, Space After: 0 pt
Formatted: Font: 11 pt, Font color: Auto
Formatted: Font color: Auto
Formatted: Font color: Auto
Formatted: Justified, Space After: 0 pt
Formatted: Justified, Space After: 0 pt, Tab stops: Not at 3.25" + 6.5"
Formatted: Justified

ivity score					10	10							10	
Peer problems score	0-3	0-4	4-5	5	6-10	6-10	0-2	0-4	3	5	4	6	5-10	7-9
Pro-social score	6-10	6-10	5	5	0-4	0-4	7-10	7-10	6	6	5	5	0-4	0-4
Externalising score	0-5	0-5	6-10	6-7	11-20	8-20	0-5	0-5	6-10	6-7	11-12	8	13-20	9-20
Internalising score	0-4	0-8	5-8	9-10	9-20	11-20	0-4	0-8	5-8	9-10	9-10	11-12	11-20	13-20

As indicated in Fig 1, the prevalence of mental health total difficulties (15.9-25.5%), internalising (14.9-28.4%), emotional (10.4-23.9%), and peer relationship (17.8-25.8%) problems were higher compared to reports of previous studies. These mental health problems were more prevalent for female and male adolescents. Prevalence of depression ranged from 18.0%-25.5%, reportedly higher for female adolescents aged 14-16 years

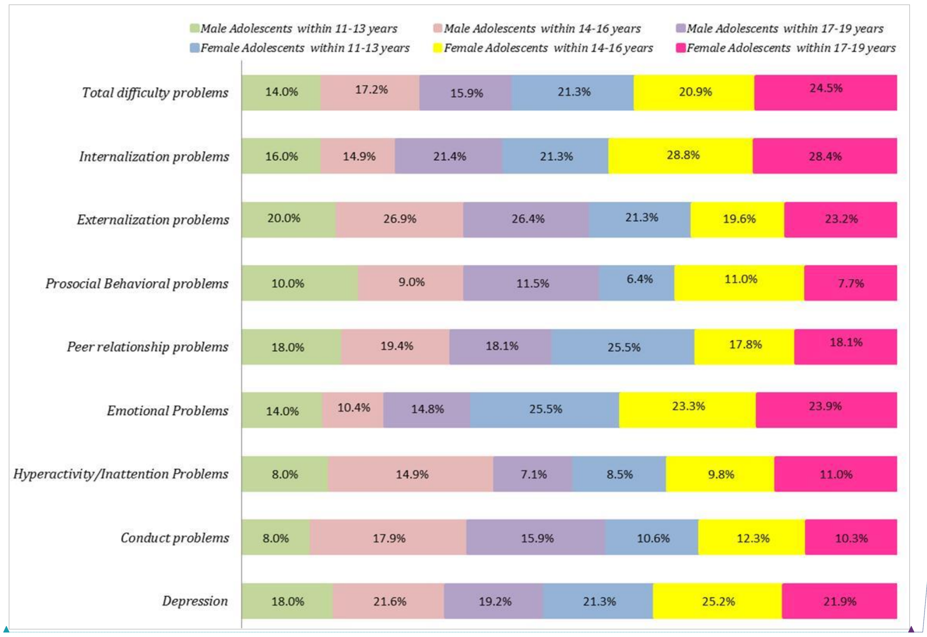

Formatted: Font color: Auto

Formatted: Font color: Auto

Figure 1: Prevalence of mental health problems among school adolescents in Dire Dawa, Ethiopia.

Formatted: Font: 12 pt, Font color: Auto, (Asian) Chinese (PRC)

3.3. Association of mental health status and socio-demographic factors

Statistical difference in prevalence was examined for each banding of subscale using the Chi-square test. The proportion of abnormal females with total difficulties and emotional and internalizing problems was significantly higher ($p<0.05$) than males. The prevalence of conduct and externalizing problems was highly associated with being male. The association of socio-demographic characteristics with mental health status was analyzed using binary logistic regression as reported in **Table 2**.

Formatted: Font color: Auto

Table 2: Chi-square test of mental health problems prevalence as per the original 3 band categorization (cut-off scores at 80th & 90th) between males and females

Formatted: Font: 12 pt, Font color: Auto, (Asian) Chinese (PRC)

Formatted: Justified

Subscales and bandings	Males (n=366)		Females (n=365)		df	X^2	p
	n	%	n	%			
Total difficulties					2	6.913[a]	0.042
Normal(0-14)	307	83.9%	283	77.5%			
Borderline(15-17)	29	7.9%	48	13.2%			
Abnormal(18-40)	30	8.2%	34	9.3%			
Emotional problems score					2	14.442[a]	0.001
Normal0-5)	318	86.9%	278	76.2%			
Borderline=6	23	6.3%	36	9.9%			
Abnormal(7-10)	25	6.8%	51	14.0%			
Conduct problems							
Normal(0-3)	309	84.4%	324	88.8%	2	3.674[a]	0.159
Borderline=4	30	8.2%	18	4.9%			
Abnormal(5-10)	25.0	7.4%	25.0	6.3%			
Hyperactivity score					2	0.488[a]	0.783
Normal (0-4)	329	89.9%	328	89.9%			
Borderline=5	21	5.7%	18	4.9%			
Abnormal(6-10)	16	4.4%	19	5.2%			

Formatted ...
Formatted ...
Formatted ...
Formatted ...
Formatted ...
Formatted ...
Formatted ...
Formatted ...
Formatted ...
Formatted ...
Formatted ...
Formatted ...
Formatted ...
Formatted ...
Formatted ...
Formatted ...
Formatted ...
Formatted ...
Formatted ...
Formatted ...
Formatted ...
Formatted ...
Formatted ...
Formatted ...
Formatted ...
Formatted ...
Formatted ...
Formatted ...
Formatted ...
Formatted ...
Formatted ...
Formatted ...
Formatted ...
Formatted ...
Formatted ...
Formatted ...
Formatted ...
Formatted ...
Formatted ...
Formatted ...
Formatted ...
Formatted ...
Formatted ...
Formatted ...
Formatted ...
Formatted ...
Formatted ...
Formatted ...
Formatted ...
Formatted ...
Formatted ...
Formatted ...
Formatted ...
Formatted ...
Formatted ...
Formatted ...
Formatted ...
Formatted ...
Formatted ...
Formatted ...

Peer problems score					2	0.930[a]	0.628
Normal(0-4)	298	81.4%	296	81.1%			
Borderline=5	41	11.2%	36	9.9%			
Abnormal(6-10)	27	7.4%	33	9.0%			
Internalising score					2	9.332[a]	0.009
Normal(0-8)	299	81.7%	264	72.3%			
Borderline(9-10)	36	9.8%	50	13.7%			
Abnormal(11-20)	31	8.5%	51	14.0%			
Externalising score					2	1.896[a]	0.387
Normal(0-5)	272	74.3%	287	78.6%			
Borderline(6-7)	50	13.7%	41	11.2%			
Abnormal(8-20)	44	12.0%	37	10.1%			
Pro-social score					2	0.549[a]	0.760
Normal(6-10)	328	89.6%	332	91.0%			
Borderline=5	18	4.9%	14	3.8%			
Abnormal(0-4)	20	5.5%	19	5.2%			

A binary logistic regression model analysis showed that the odds of mental health problems were significantly associated with some socio-demographic characteristics (p<0.05); odds were different across these characteristics as reported in **Table 3**. The prevalence of mental health problems was significantly higher for females in upper elementary (AOR: 2.60 (0.95-7.10)) and lower secondary levels (AOR: 2.73 (1.19-6.29)) compared to upper secondary grade level (p<0.05); but it was not significantly different for males. It was twice higher

among adolescents with a self and family members experiencing psychoactive substance use ($p<0.05$). Prevalence difference across age, maternal and paternal education, and employment status was insignificant ($p>0.05$).

Table 3: Association of mental health problems and socio-demographic determinants analyzed with binary logistic regression

		Prevalence of mental health problem %			
Predictors		Male		Female	
	Categories	UAOR(95% CI)	AOR(95% CI)	UAOR(95% CI)	AOR(95% CI)
	11-19	-	-		-
Age group(yrs)	11-13	1	1	1	1
	14-16	1.27(0.51-3.18)	0.959(0.25-3.7)	0.98 (0.44-2.16)	0.65 (0.20-2.06)
	17-19	1.16(0.48-2.84)	0.643(0.06-6.28)	1.20 (0.55-2.64)	0.53 (0.08-3.38)
Grade level	Upper elementary (5-8)	1.08(0.51-2.31)	1.44(0.50-4.15)	1.57 (0.70-3.50)	2.60 (0.95-7.10)
	Lower Secondary	1.02(0.48-	1.11(0.50-	2.22 (1.00-	2.73

Formatted: Font: 11 pt, Font color: Auto
Formatted: Font color: Auto
Formatted: Justified, Space After: 0 pt
Formatted: Font color: Auto
Formatted: Font: 11 pt, Font color: Auto
Formatted: Font color: Auto
Formatted: Justified, Space After: 0 pt
Formatted: Font: 11 pt, Font color: Auto
Formatted: Font color: Auto
Formatted: Justified, Space After: 0 pt
Formatted: Justified, Space After: 0 pt
Formatted: Font: 11 pt, Font color: Auto
Formatted: Font color: Auto
Formatted: Justified, Space After: 0 pt
Formatted: Font color: Auto
Formatted: Font color: Auto
Formatted: Justified, Space After: 0 pt
Formatted: Font: 11 pt, Font color: Auto
Formatted: Font color: Auto
Formatted: Indent: Left: 0", Right: 0", Space After: 0 pt, Adjust space between Latin and Asian text, Adjust space between Asian text and numbers
Formatted: Justified, Space After: 0 pt
Formatted: Font color: Auto
Formatted: Justified, Indent: Left: 0", Right: 0", Space After: 0 pt, Adjust space between Latin and Asian text, Adjust space between Asian text and numbers
Formatted: Justified, Space After: 0 pt
Formatted: Font color: Auto
Formatted: Font: 11 pt, Font color: Auto
Formatted: Font color: Auto
Formatted: Justified, Space After: 0 pt
Formatted: Font color: Auto
Formatted: Justified, Space After: 0 pt
Formatted: Justified

	(9-10)	2.18)	2.46)	4.92)*	(1.19-6.29)*
	Upper secondary (11-12)	1	1	1	1
Experience on psychoactive substances use					
Self experience	No	1	1	1	1
	Yes	0.45(0.22-0.93*	2.20(1.07-4.52)*	2.17 (0.95-4.95)	2.16 (0.95-4.92)
Any family experience	No	1	1	1	1
	Yes	2.01(1.01-4.00)*	2.04(1.01-4.09)*	1.07(0.63-1.84)	1.06 (0.62-1.81)
Parents practicing corporal punishment	No	1	1	1	1
	Yes	1.24 (0.69-2.24)	1.24 (0.69-2.23)	1.43 (0.82-2.51)	1.33 (0.75-2.36)
Adolescents report perceived worry about family	No	1	1	1	1
	Yes	1.186(0.64-2.21)	1.17(0.62-2.20)	1.24 (0.73-2.12)	1.20 (0.70-2.06)

Formatted: Font color: Auto
Formatted: Justified, Space After: 0 pt
Formatted: Font color: Auto
Formatted: Font color: Auto
Formatted: Font: 11 pt, Font color: Auto
Formatted: Font color: Auto
Formatted: Font color: Auto
Formatted: Justified, Space After: 0 pt
Formatted: Font: 11 pt, Font color: Auto
Formatted: Font color: Auto
Formatted: Justified, Space After: 0 pt
Formatted: Font color: Auto
Formatted: Font color: Auto
Formatted: Justified, Space After: 0 pt
Formatted: Font: 11 pt, Font color: Auto
Formatted: Font color: Auto
Formatted: Justified, Space After: 0 pt
Formatted: Font color: Auto
Formatted: Font color: Auto
Formatted: Justified, Space After: 0 pt
Formatted: Font color: Auto
Formatted: Justified, Space After: 0 pt
Formatted: Font: 11 pt, Font color: Auto
Formatted: Font color: Auto
Formatted: Font color: Auto
Formatted: Justified, Space After: 0 pt
Formatted: Font color: Auto
Formatted: Justified, Space After: 0 pt
Formatted: Justified

problem					
Mother education level	Non-educated	1	1	1	1
	Elementary	1.31(0.50-3.43)	1.30 (0.49-3.41)	1.99 (0.57-6.96)	0.73 (0.29-1.87)
	Secondary	1.30(0.66-2.57)	1.30 (0.66-2.58)	1.50 (0.34-6.70)	1.37 (0.78-2.42)
	College or above	0.63(0.18-2.20)	0.64 (0.18-2.24)	2.71 (0.74-9.93)	0.52 (0.15-1.81)
Father education level	Non-educated	1	1	1	1
	Elementary	1.51 (0.47-4.82)	1.48 (0.46-4.74	1.03 (0.21-5.14)	1.00 (0.20-4.99)
	Secondary	1.60 (0.83-3.09)	1.61 (0.83-3.11	1.04 (0.58-1.84)	1.07 (0.60-1.90)
	college or above	1.42 (0.64-3.16)	1.45 (0.65-3.23	1.23 (0.61-2.46)	1.20 (0.60-2.41)
Mother job	House wife/Unemployed	1	1	1	1

	Work in private	0.79 (0.41-1.51)	0.79 (0.41-1.51)	1.40 (0.77-2.53)	1.40 (0.77-2.54)
	Employed	0.44 (0.16-1.22)	0.44 (0.16-1.21)	0.55 (0.22-1.42)	0.55 (0.21-1.44)
Father job	Unemployed/Jobless	1	1	1	1
	Work in private	1.24 (0.66-2.32)	1.24 (0.66-2.33)	0.75(0.43-1.33)	0.77(0.44-1.36)
	Employed	2.56 (0.75-8.74)	2.52 (0.73-8.70)	ns	ns

** Significant at p<0.01 * Significant at p<0.05

However, the prevalence of mental health problems was not significantly different for males by education level. Prevalence difference was insignificant across the three age groups (11-13, 14-16, and 17-19 years), maternal and paternal education level, and types of jobs or level of employment (p>0.05). The prevalence of mental health problems was twice higher among psychoactive substance users (p<0.05) compared with non-users. Differences in prevalence existed but were insignificant across these three age groups, maternal and paternal education level, and types of jobs or level of employment (p>0.05).

Formatted: Font color: Auto
Formatted: Justified, Space After: 0 pt
Formatted: Font color: Auto
Formatted: Justified, Space After: 0 pt
Formatted: Font color: Auto
Formatted: Font: 11 pt, Font color: Auto
Formatted: Font color: Auto
Formatted: Justified, Space After: 0 pt
Formatted: Font color: Auto
Formatted: Justified, Space After: 0 pt
Formatted: Font color: Auto
Formatted: Justified, Space After: 0 pt
Formatted: Font color: Auto
Formatted: Space Before: 0 pt, After: 10 pt
Formatted: Space Before: 0 pt
Formatted: Justified

3.4. Correlation of mental health literacy with total difficulty scores and well-being index

The bivariate correlations showed that the mental health literacy score was negatively associated with the total and subscale strength difficulties scores (conduct problems, emotional problems, hyperactivity-inattention, and peer problems) as it is shown in **Table 4**.

Table 4: The correlations between mean mental health literacy score, strength difficulties questionnaire scale, and mental wellbeing index

Variables		Correlation coefficient (r)) with 95%CI								
		1	2	3	4	5	6	7	8	9
1.	SDQ Total difficulties problems score	1	-.319** (-.388-(-.250))	-.124** (-.198-(-.052))	.652** (.607-.694)	.700** (.656-.740)	.749** (.714-.783)	617** (.570-.659)	.825** (.798-.848)	.863** (.842-.882)
2.	Mental wellbeing index	-	1	.160** (.084-	-.140** (-	-.281** (-	-.293** (-	-.130** (-.200-	-.258** (-.329-	-.279** (-.347-

Formatted: Font: (Default) Cambria, 16 pt, Bold, Font color: Auto, (Asian) Chinese (PRC)
Formatted: Font: 16 pt, Font color: Auto
Formatted: Font color: Auto
Formatted: Space After: 10 pt
Formatted: Font: (Default) Times New Roman, Bold, Not Italic, Font color: Auto
Formatted: Font color: Auto
Formatted: Font: (Default) Times New Roman, Bold, Not Italic, Font color: Auto
Formatted: Space After: 6 pt
Formatted: Font color: Auto
Formatted: Font color: Auto
Formatted: Justified, Space After: 10 pt, Line spacing: Double
Formatted: Font: 11 pt, Font color: Auto
Formatted: Font color: Auto
Formatted: Justified, Space After: 10 pt, Line spacing: Double
Formatted: Font color: Auto
Formatted: Justified, Space After: 10 pt, Line spacing: Double
Formatted: Font color: Auto
Formatted: Space After: 10 pt, Line spacing: Double
Formatted: Font: 11 pt, Font color: Auto
Formatted: Justified, Space After: 10 pt, Line spacing: Double
Formatted: Justified, Space After: 10 pt, Line spacing: Double
Formatted: Font color: Auto
Formatted: Font color: Auto
Formatted: Space After: 10 pt, Line spacing: Double
Formatted: Font: 11 pt, Font color: Auto
Formatted: Justified, Space After: 10 pt, Line spacing: Double
Formatted: Justified, Space After: 10 pt, Line spacing: Double
Formatted: Justified

				.234)	.206-(-.076))	.350-(-.210))	.366-(-.215))	(-.060	(-.186))	(-.209))
3.	MHL Total mean score	-	-	1	-.135** (-.211-(-.064))	-.136** (-.212-(-.058))	-0.006 (-.078-.058)	-.088* (-.165-(-.010))	-.166** (-.244-(-.087))	-0.051 (-.120-.016)
4.	SDQ Conduct problems subscale mean score	-	-	-	1	.345** (.275-.412)	.228** (.159-.301)	.294** (.220-.368)	.812** (.785-.837)	.319** (.249-.388)
5.	SDQ Hyperactivity problems subscale mean score	-	-	-	-	1	.385** (.310-.454)	.197** (.125-.265)	.828** (.802-.854)	.380** (.310-.446)
6.	SDQ Emotional problems subscale	-	-	-	-	-	1	.272** (.195-.342)	.376** (.301-.453)	.862** (.844-.880)

Formatted: Font color: Auto
Formatted: Justified, Space After: 10 pt, Line spacing: Double
Formatted: Font color: Auto
Formatted: Font: 11 pt, Font color: Auto
Formatted: Space After: 10 pt, Line spacing: Double
Formatted: Justified, Space After: 10 pt, Line spacing: Double
Formatted: Font color: Auto
Formatted: Font color: Auto
Formatted: Space After: 10 pt, Line spacing: Double
Formatted: Font: 11 pt, Font color: Auto
Formatted: Justified, Space After: 10 pt, Line spacing: Double
Formatted: Justified, Space After: 10 pt, Line spacing: Double
Formatted: Font color: Auto
Formatted: Font color: Auto
Formatted: Font: 11 pt, Font color: Auto
Formatted: Space After: 10 pt, Line spacing: Double
Formatted: Justified, Space After: 10 pt, Line spacing: Double
Formatted: Justified, Space After: 10 pt, Line spacing: Double
Formatted: Font color: Auto
Formatted: Font color: Auto
Formatted: Space After: 10 pt, Line spacing: Double
Formatted: Font: 11 pt, Font color: Auto
Formatted: Justified, Space After: 10 pt, Line spacing: Double, Tab stops: Not at 3.25" + 6.5"
Formatted: Justified, Space After: 10 pt, Line spacing: Double
Formatted: Justified

	mean score									
7.	SDQ Peer problems subscale mean score	-	-	-	-	-	-	1	.298** (.231-.362)	.721** (.683-.756)
8.	SDQ Externalizing Problems subscale mean score	-	-	-	-	-	-	-	1	.427** (.360-.489)
9.	SDQ Internalizing problems subscale mean score	-	-	-	-	-	-	-	-	1

** Correlation is significant at the 0.01 level (2-tailed); * Correlation is significant at the 0.05 level (2-tailed

Formatted: Font color: Auto

Formatted: Font color: Auto

Formatted: Space After: 10 pt, Line spacing: Double

Formatted: Font: 11 pt, Font color: Auto

Formatted: Justified, Space After: 10 pt, Line spacing: Double

Formatted: Justified, Space After: 10 pt, Line spacing: Double

Formatted: Font color: Auto

Formatted: Font color: Auto

Formatted: Space After: 10 pt, Line spacing: Double

Formatted: Font: 11 pt, Font color: Auto

Formatted: Justified, Space After: 10 pt, Line spacing: Double

Formatted: Justified, Space After: 10 pt, Line spacing: Double

Formatted: Font color: Auto

Formatted: Font color: Auto

Formatted: Font: 11 pt, Font color: Auto

Formatted: Space After: 10 pt, Line spacing: Double

Formatted: Justified, Space After: 10 pt, Line spacing: Double

Formatted: Font color: Auto

Formatted: Justified

4. Discussion

The Ethiopian government has taken several interventions to mitigate the adolescent mental health issue, establishing mental health facilities nationwide.[67] The government has also trained health professionals in mental health care who are better equipped to identify and treat mental health problems in adolescents—implementing psycho-education programs in schools, public awareness campaigns, and training for community leaders and media outlets for adolescents, among others.[4] The state mechanism in promoting mental health literacy in Ethiopia is that the Ministry of Health is responsible for developing and implementing mental health policies and programs and provides training for health professionals in mental health care.[4] Stakeholders including professional associations, such as The Ethiopian Mental Health Association and The Ethiopian Psychiatric Association, are involved in policy consultation, provide training and support for health professionals, and promote mental health research in Ethiopia. These policy and programs are cascaded along the government administrative levels for actual implementation. However, there is still a paucity of evidence about the mental health status of adolescents and factors that affect mental health problems and factors that contribute for effective interventions to improve adolescents' mental health.

The SDQ is a screening tool for mental health problems in children and adolescents. It is scored on a scale of 0 to 40, with higher scores indicating more problems. The original cut-off scores for the SDQ are 80 and 90, with scores above 90 being considered abnormal, scores between 80 and 90 being considered borderline, and scores below 80 being considered normal. [27,53] The cut-off score for the present study was slightly lower for total difficulties and subscales for hyperactivity scores than the baseline and relatively higher for internalizing scores (**Table 1**). These cut-off scores for the present study were almost similar to the

Formatted: Font: 18 pt, Font color: Auto
Formatted: Font color: Auto
Formatted: Font: 18 pt, Font color: Auto
Formatted: Font color: Auto
Formatted: Space Before: 24 pt
Formatted: Normal (Web), Line spacing: Double
Formatted: Font: Not Bold, Font color: Auto
Formatted: Font color: Auto
Formatted: Font: English (United States)
Formatted: Font color: Auto
Formatted: Font color: Auto
Formatted: Font color: Auto
Formatted: Justified

baseline definition that was taken from the UK child and adolescent population during the first version of the Strengths and Difficulties Questionnaire (SDQ) development. [27,53]

Using cut-off values from local population scores ensures cultural equivalence; that supports relevancy and applicability of the instrument to the cultural groups.[68] As indicated in Table 1, the study showed that the reference scores range taken from a present study for hyperactivity and internalizing scores were higher compared to baseline reference scores ranges. [27,53] The reference scores from a present study using the original 3 band categories (cut-off scores at 80th & 90th) for conduct problems, emotional problems, peer problems, and pro-social scores were similar to the baseline reference scores ranges.[27,53] The reference scores range taken from a present study for total difficulties, hyperactivity, and externalizing scores were lower than baseline reference scores. [27,53] If the cut-off score for a given population is higher than the original cut-off score, it means that a more significant proportion of children in that population are experiencing mental health problems.[69] This could be due to factors such as poverty, social disadvantage, or exposure to trauma.[70]

It is important to note that the SDQ is a screening tool, not a diagnostic tool.[69] If a child scores above the cut-off score, it does not necessarily mean they have a mental health problem.[69] However, it does indicate that they are at an increased risk of having a problem, and further assessment is warranted. Considering these factors is essential when interpreting a lower cut-off score from the baseline. Some evidence indicated that lower cut-off values have been more effective than the higher cut-off values at identifying child and adolescent mental health problems.[68]

These findings implied that the adolescent population in urban Ethiopia is more disadvantaged than the population on which the original cut-off scores were based. Adolescents in urban Ethiopia have been exposed to stressors like poverty, violence, or

trauma. This adolescent population has less access to mental health services and is more likely to underreport mental health problems.

As shown on **Fig. 1,** the present finding was within the same range of the Ethiopian national level prevalence of adolescent mental health problems, reportedly ranging from 17-23%, like other study results in the Ethiopian context.[71–73] Prevalence of total difficulties (15.9-25.5%), internalising (14.9-28.4%), emotional (10.4-23.9%), and peer relationship (17.8-25.8%) was similar that was within the range of global prevalence(10-20%).[74]

Several studies showed discrepancies in adolescent mental health problems prevalence from region to region and country to country.[70,75–77] Similarly, these differences exist across socio-demographic characteristics. [78] From the present study, mental health problems expressed by the total strength difficulties and its subscales of problems were higher among female adolescents than male adolescents. A study among Indian children, the association between the female gender and total strength difficulties score was significant.[75] Being female was associated with emotional problems.[75] A cross-national study across 73 countries on the gender gap in adolescent mental health showed that girls have worse average mental health than boys.[79] The possible explanations include but are not limited to socio-cultural factors, gender stereotypes, trauma, and abuse that disproportionally affect girls.[22,80] In contrast, conduct and hyperactivity/inattention problems were significantly more severe among males than females.[66] Consistent with other studies, the prevalence of conduct and externalizing problems was more significant among male adolescents than females. For example, a study in Northeast china showed a similar result.[78] A study among children and adolescents from seven European countries (Italy, Netherlands, Germany, Romania, Bulgaria, Lithuania, and Turkey) reported that externalized problems were consistently higher in males than females and reversed for internalized problems.[76] One possible explanation might be that mental health status is shaped by social, economic, and physical environments leading to

inequalities that disproportionally affect gender differences and are heavily associated with risk factors for many common mental disorders.[70] According to the WHO, the relationship between the prevalence of mental health problems and poverty indices was statistically significant.[77] These poverty indices are education disparity, low income, a lack of material goods, job, and housing obstacles. For several cultural and traditional reasons, these factors impact females more than boys.[77]

Odds of mental health problems were significantly associated with some socio-demographic characteristics ($p<0.05$) differing in magnitude across these characteristics. In this study, the prevalence of mental health problems was significantly higher for females in upper elementary and lower secondary levels than for upper secondary grade levels ($p<0.05$), consistent with reports of several studies.[70,77] According to WHO report, educational status is one of the major factors determining adolescent mental health outcomes.[77]

However, the difference was insignificant for male adolescents. Furthermore, differences in prevalence have existed but little across the three age groups, maternal and paternal education level, and job types or level of employment ($p>0.05$). The present study's prevalence of mental health problems was twice higher among adolescents with a self and/or family members' experience of psychoactive substance use ($p<0.05$). On the contrary, a study in Northeast China showed that the prevalence of any mental disorders and internalizing disorders was significantly lower in younger adolescents than in elders. Children aged 11–14 years had the highest prevalence of internalizing disorders.[78] It was also inconsistent with a study in developing countries showing that lower maternal education was significantly associated with abnormal total SDQ scores.[81]

The present study revealed mental health literacy was negatively correlated with strength difficulties scores and positively correlated with mental well-being; both were significantly

Formatted: Justified

correlated but weaker in magnitude. These findings were consistent with existing theories and the results of previous studies. For instance, a study demonstrated that an inadequate level of mental health literacy was negatively associated with depression. The prevalence of depression was 1.52 times higher in those with levels of inadequate MHL than in those who demonstrated an adequate level of MHL.[50] Another study depicted MHL as an explanatory variable of the mental well-being of adolescents having a positive correlation with it. [82]

A possible explanation for the negative association between mental health literacy and strength difficulties scores might be that low mental health literacy contributes to the mental health promotion and prevention gap.[45,83–85] Low mental health literacy exacerbates barriers and difficulties in accessing treatment for mental health problems.[86]

Mental health literacy significantly influences adolescents' perceptions and emotional responses [87], reflecting mental health status and subjective mental well-being. Several studies revealed that mental health literacy had a significant relationship with mental health status. [88–90] A higher level of mental well-being has been reported among adolescents with better know-how about obtaining mental health services.[87] Adolescents' awareness of symptoms, causes, and mental illness treatment contributes to favorable attitudes toward seeking help.[15] Similarly, adolescents with better mental health literacy are less likely to engage in problematic health behaviours and have better help-seeking intention and self-efficacy, contributing to a better mental health and well-being.[91] Mental health literacy is about the information, attitudes, and awareness of mental health concerns that improve self-efficacy, help-seeking intention, and health behaviour, influencing long-term health outcomes and quality of life. A lack of understanding of the symptoms and nature of mental health disorders and alternate sources of support could explain teenagers' low help-seeking intention and behaviour. [15]

Formatted: Justified

Of course, help-seeking intention and self-efficacy may reflect only one of several mechanisms. Better mental health literacy leads to better mental well-being. Higher help-seeking intention and self-efficacy would only partially mediate the relation between higher mental health literacy and better mental well-being. Components of health behavior expressed in actions, targets, contexts, and time are the most determining factors of mental health outcomes.[92,93]

Mental health literacy influences mental health services and help-seeking for treatment.[94] Hence, mental health literacy is essential to improve and often but not always corresponds to help-seeking behaviours. [95] Anthony F. Jorm and his colleagues revealed that changing knowledge in principle is vital and not problematic; however, changing heartily emotional reactions to practice caring and preventing mental disorders may be much more challenging.[10]

Help-seeking, interchangeably health-seeking, has become one of the essential perspectives in understanding causes and factors for patient delay from appropriate action across different health conditions.[96] Despite the prevalent mental health problems and poor well-being, adolescents' unwillingness and low intention to seek help usually result in delays in timely treatment.[43] Most adolescents with mental health problems are reluctant and miss the use of healthcare for mental health, which increases the complexity of social, mental, and general health outcomes.[97] The findings from this study implied that promoting mental health literacy can improve subjective mental well-being.[87]

The recently revised policy released by The Ethiopian Ministry of Health in 2017 aimed to improve adolescents' mental health by providing early identification, prevention, and treatment services. [4,21] Following the policy, programs were launched in 2018, including school-based mental health programs, community-based mental health services, and training

Formatted: Space After: 10 pt

Formatted: Font: Not Bold

Formatted: Font color: Auto

Formatted: Font color: Auto

Formatted: Font color: Auto

Formatted: Justified

for health care providers.[21] Empirical evidence on early identification of vulnerable groups, individuals reporting symptoms of mental health difficulties and the associated actors could enhance the impact and effectiveness of these programs implementation.[4,21] In this regard, demographic and socioeconomic sensitive approaches should be applied to mental health policies for mental health promotion and mental illness prevention.

5. Limitations

The study has some inherent limitations. It is not applicable to generalize these findings across the country; because Ethiopia exhibits multicultural and multiethnic diversity and multiple socio-demographic backgrounds. It could not even be generalized to all adolescents of the study area, because the samples were selected from the age group (11-19) and grade level (5-12), adolescents at the age of 10 years and lower grade levels were excluded during the first cycle of elementary schools during school sampling. The present study is conducted on the urban adolescents and did not address rural adolescents. The study was quantitative and lacked the qualitative aspect of how and why these findings were observed.

6. Conclusion

The cut-off scores for the present study were almost similar to the baseline definition of the first version of the Strengths and Difficulties Questionnaire (SDQ) development that was taken from the UK child and adolescent population. However, the strength difficulty cut-off score for total strength difficulty and hyperactivity scores was slightly lower and the cut-off score for internalizing scores was slightly higher. The finding was within the same range as the Ethiopian national level prevalence of adolescent mental health problems, reportedly ranging from 17-23%. The prevalence of total difficulties (15.9-25.5%), internalizing (14.9-

Formatted: Font color: Auto

Formatted: Font color: Auto

Formatted: Font color: Auto

Formatted: Font color: Auto

Formatted: Font: (Default) Cambria, 18 pt, Bold, Font color: Auto, (Asian) Chinese (PRC)

Formatted: Font: 18 pt, Font color: Auto

Formatted: Font color: Auto

Formatted: Space After: 10 pt

Formatted: Font: (Default) Cambria, 18 pt, Bold, Font color: Auto, (Asian) Chinese (PRC)

Formatted: Font: 18 pt, Font color: Auto

Formatted: Font color: Auto

Formatted: Indent: First line: 0"

Formatted: Justified

28.4%), emotional (10.4-23.9%), and peer relationship problems (17.8-25.8%) were similar that was slightly within the range of the global prevalence(10-20%). These mental health problems were significantly greater among adolescents who had either themselves or their family members used psychoactive substances ($p \leq 0.05$) ($p < 0.05$). As depicted from strength difficulties scores, the mental health problems were significantly associated with some socio-demographic characteristics ($p < 0.05$) differing in magnitude.

Female adolescents scored higher scores on the total difficulty and its subscales (internalizing and externalizing scores) than males, implying that the prevalence of mental health problems was higher among females than their male counterparts, aligning with previous study findings. These findings highlight the need to improve adolescent mental health literacy in urban Ethiopia to improve mental health status. Gender, grade level, age, maternal and paternal education level, job types, and level of employment are factors that affect adolescents' mental health problems. Hence, promoting adolescent mental health through improving mental health literacy requires congruent approaches considering these socio-demographic factors.

The study showed a correlation between mental health literacy level, the strength difficulty scale, and mental well-being scores. It showed mental health literacy negatively correlated with strength difficulties scores and positively associated with mental well-being. These findings were consistent with existing theories and the results of previous studies. It implied that promoting mental health literacy could enhance adolescents' positive mental health, and priorities should be on vulnerable groups and individuals reporting symptoms of mental health difficulties. Future studies should involve qualitative and include the remaining determinants.

Formatted: Font: Times New Roman, Font color: Auto
Formatted: Font color: Auto
Formatted: Space After: 10 pt
Formatted: Justified

Acknowledgments

The authors would like to thank the survey respondents for their participation and contributions to the study.

Authors' contributions

Hassen, H.M and Behera M.R conceptualized the study design. Hassen, H.M collected, analyzed, and interpreted the data used in this study and was a major contributor to writing the article. Behera, D, Behera, M.R. and Dehury, R. K. assisted in analyzing and interpreting the data and writing the paper. All the authors read, commented, and approved the final article.

References

1. Renwick L, Pedley R, Johnson I, Bell V, Lovell K, Bee P, et al. Mental health literacy in children and adolescents in low- and middle-income countries: a mixed studies systematic review and narrative synthesis. Eur Child Adolesc Psychiatry [Internet] 2022;(0123456789). Available from: https://doi.org/10.1007/s00787-022-01997-6
2. Sodi T, Quarshie ENB, Oppong Asante K, Radzilani-Makatu M, Makgahlela M, Nkoana S, et al. Mental health literacy of school-going adolescents in sub-Saharan Africa: a regional systematic review protocol. BMJ Open 2022;12(9):1–7.
3. Kucera M, Tomaskova H, Stodola M, Kagstrom A. A Systematic Review of Mental Health Literacy Measures for Children and Adolescents. Adolesc Res Rev [Internet] 2023;8(3):339–58. Available from: https://doi.org/10.1007/s40894-022-00202-8
4. Ministry of Health Federal Democratic Republic of Ethiopia. Ethiopian National Mental Health Strategy (2020-2025). Fed Minist Heal 2020;2025.
5. Hassen HM, Behera MR, Jena PK, Dewey RS DG. Mental Health Literacy of Adolescents and the Effect of Socio- demographic Characteristics : A Cross-sectional Study. Online J Heal allied Sci [Internet] 2021;2–8. Available from: https://www.ojhas.org/issue80/2021-4-6.html
6. Claire M Kelly, Anthony F Jorm, Anne Marie Wright. Improving mental health literacy as a strategy [Internet]. Med. J. Aust. Med. J. Aust.2007;187(187):25–729. Available from: www.mja.com.au
7. Kelly CM, Jorm AF, Wright A. Improving mental health literacy as a strategy to facilitate early intervention for mental disorders. 2007;187(7):1–5.
8. Jorm AF. Why We Need the Concept of "Mental Health Literacy." Health Commun 2015;30(12):1166–8.

9. Jorm AF, Korten AE, Jacomb PA, Christensen H, Rodgers B, Pollitt P. “Mental health literacy”: A survey of the public’s ability to recognise mental disorders and their beliefs about the effectiveness of treatment. Med J Aust 1997;166(4):182–6.
10. Jorm A. F. et al. Research on mental health literacy: What we know and what we still need to know. Aust N Z J Psychiatry [Internet] 2006;40(1):3–5. Available from: http://www.embase.com/search/results?subaction=viewrecord&from=export&id=L43337434%5Cnhttp://dx.doi.org/10.1111/j.1440-1614.2006.01734.x%5Cnhttp://sfx.metabib.ch/sfx_locater?sid=EMBASE&issn=00048674&id=doi:10.1111/j.1440-1614.2006.01734.x&atitle=Research+
11. Patton GC, Olsson CA, Skirbekk V, Saffery R, Wlodek ME, Azzopardi PS, et al. Adolescence and the next generation. Nature [Internet] 2018;554(7693):458–66. Available from: http://dx.doi.org/10.1038/nature25759
12. Steel Z, Marnane C, Iranpour C, Chey T, Jackson JW, Patel V, et al. The global prevalence of common mental disorders: A systematic review and meta-analysis 1980-2013. Int J Epidemiol 2014;43(2):476–93.
13. O’Sullivan J and AE. Healthy Lives: Widening The Focus On Mental Health Annual Public Health Report 2015 Camden and Islington. Camden Islingt Annu Public Heal Rep 2015;1–148.
14. David K, Park MJ, Mulye TP. The Mental Health of Adolescents: A National Profile, 2008. San Francisco: Natl. Adolesc. Health Inf. Cent. (NAHIC), Univ. Calif. 2008;
15. Eschenbeck H, Lehner L, Hofmann H, Bauer S, Becker K, Diestelkamp S, et al. The Mental Health of Adolescents: A National children and adolescents with StresSOS using online or face-to-face interventions: study protocol for a randomized controlled trial within the ProHEAD Consortium. Trials [Internet] 2019;20(1):94. Available from: https://trialsjournal.biomedcentral.com/articles/10.1186/s13063-018-3157-7
16. La Maison C, Munhoz TN, Santos IS, Anselmi L, Barros FC, Matijasevich A. Prevalence and risk factors of psychiatric disorders in early adolescence: 2004 Pelotas (Brazil) birth cohort. Soc Psychiatry Psychiatr Epidemiol [Internet] 2018;53(7):685–97. Available from: http://dx.doi.org/10.1007/s00127-018-1516-z
17. Mills KL. Social development in adolescence: brain and behavioural changes [Internet]. Univ. Coll. London2015;Available from: https://discovery.ucl.ac.uk/1468865/1/KateMills_Thesis_FINAL_Appendices.pdf
18. Manganello JA. Health literacy and adolescents: A framework and agenda for future research. Health Educ Res 2008;23(5):840–7.
19. Shahraki-Mohammadi A, Panahi S, Ashouri A, Sayarifard A. Important Systemic Factors for Improving Adolescent Mental Health Literacy. Iran J Psychiatry 2023;18(1):45–54.
20. Seboka BT, Hailegebreal S, Negash M, Mamo TT, Ewune HA, Gilano G, et al. Predictors of Mental Health Literacy and Information Seeking Behavior Toward Mental Health Among University Students in Resource-Limited Settings. Int J Gen Med 2022;15(November):8159–72.
21. Hanlon C, Alem A, Lund C, Hailemariam D, Assefa E, Giorgis TW, et al. Moving towards universal health coverage for mental disorders in Ethiopia. Int J Ment Health Syst [Internet] 2019;13(1):1–17. Available from: https://doi.org/10.1186/s13033-019-0268-9
22. Eaton NR, Keyes KM, Krueger RF, Balsis S, Skodol AE, Markon KE, et al. An invariant dimensional liability model of gender differences in mental disorder prevalence: evidence from a national sample. J Abnorm Psychol 2012;121(1):282–8.
23. Fok MSMM, Wong TKSS. What does health literacy mean to children? Contemp nurse a J Aust Nurs Prof 2002;13(2–3):249–58.

Formatted: Justified

24. Goodman R, Ford T, Simmons H, Gatward R, Meltzer H. Using the Strengths and Difficulties Questionnaire (SDQ) to screen for child psychiatric disorders in a community sample. Int Rev Psychiatry 2003;15(1–2):166–72.
25. Skinner D, Sharp C, Marais L, Serekoane M, Lenka M. Assessing the Value of and Contextual and Cultural Acceptability of the Strength and Difficulties Questionnaire (SDQ) in Evaluating Mental Health Problems in HIV/AIDS Affected Children. Int J Ment Health 2014;43(4):76–89.
26. Chongwo E, Ssewanyana D, Nasambu C, Mwangala PN, Mwangi PM, Nyongesa MK, et al. Validation of a Swahili version of the World Health Organization 5-item well-being index among adults living with HIV and epilepsy in rural coastal Kenya. Glob Heal Res Policy 2018;3(1):3–9.
27. Goodman R. The strengths and difficulties questionnaire: A research note. J Child Psychol Psychiatry Allied Discip 1997;38(5):581–6.
28. Atilola O, Singh Balhara YP, Stevanovic D, Avicenna M, Kandemir H. Self-Reported Mental Health Problems Among Adolescents in Developing Countries. J Dev Behav Pediatr [Internet] 2013;34(2):129–37. Available from: http://content.wkhealth.com/linkback/openurl?sid=WKPTLP:landingpage&an=00004703-201302000-00008
29. Goodman A, Lamping DL, Ploubidis GB. When to use broader internalising and externalising subscales instead of the hypothesised five subscales on the strengths and difficulties questionnaire (SDQ): Data from british parents, teachers and children. J Abnorm Child Psychol 2010;38(8):1179–91.
30. Downs A, Boucher LA, Campbell DG, Polyakov A. Using the WHO–5 well-being index to identify college students at risk for mental health problems. J Coll Stud Dev 2017;58(1):113–7.
31. De Wit M, Pouwer F, Gemke RJBJ, Delemarre-Van De Waal HA, Snoek FJ. Validation of the WHO-5 well-being index in adolescents with type 1 diabetes. Diabetes Care 2007;30(8):2003–6.
32. Kusier AO, Folker AP. The Well-Being Index WHO-5: Hedonistic foundation and practical limitations. Med Humanit 2019;1–7.
33. Garland AF, Deyessa N, Desta M, Alem A, Zerihun T, Hall KG, et al. Use of the WHO's perceived Well-Being Index (WHO-5) as an efficient and potentially valid screen for depression in a low income country. Fam Syst Heal 2018;36(2):148–58.
34. Topp CW, Østergaard SD, Søndergaard S, Bech P. 1. The WHO-5 well-being index: A systematic review of the literature. Psychother Psychosom 2015;84(3):167–76.
35. Hoosen N, Davids EL, Vries PJ De, King MS. The Strengths and Difficulties Questionnaire (SDQ) in Africa : a scoping review of its application and validation. Child Adolesc Psychiatry Ment Health [Internet] 2018;1–39. Available from: https://doi.org/10.1186/s13034-017-0212-1
36. Hassen HM, Behera MR, Jena PK, Dewey RS. Effectiveness and Implementation Outcome Measures of Mental Health Curriculum Intervention Using Social Media to Improve the Mental Health Literacy of Adolescents. J Multidiscip Healthc [Internet] 2022;15(May):979–97. Available from: http://www.ncbi.nlm.nih.gov/pmc/articles/pmc9078434/
37. Maher MS and BA. Philosophy and Psychopathology. Springer-Verlag New York Inc.: Springer-Verlag New York Heidelberg Berlin London Paris Tokyo Hong Kong; 1990.
38. Brown TA, Barlow DH. Dimensional Versus Categorical Classification of Mental Disorders in the Fifth Edition of the Diagnostic and Statistical Manual of Mental Disorders and Beyond : Comment on the Special Section. 2005;114(4):551–6.

Formatted: Justified

39. Tusaie KR. Epidemiology of child psychiatric disorders in Addis Ababa, Ethiopia. Division of Child and Adolescent Psychiatry Department of Clinical Sciences; 2013.
40. Ling J, Tay YF, Klainin-Yobas P. Mental health literacy levels. Arch Psychiatr Nurs 2018;
41. World Health Organization. Adolescents: health risks and solutions: https://www.who.int/news-room/fact-sheets/detail/adolescents-health-risks-and-solutions. 2018;
42. Polanczyk G V., Salum GA, Sugaya LS, Caye A, Rohde LA. Annual research review: A meta-analysis of the worldwide prevalence of mental disorders in children and adolescents. J Child Psychol Psychiatry Allied Discip 2015;56(3):345–65.
43. Divin N, Harper P, Curran E, Corry D, Leavey G. Help-Seeking Measures and Their Use in Adolescents: A Systematic Review. Adolesc Res Rev [Internet] 2018;3(1):113–22. Available from: http://dx.doi.org/10.1007/s40894-017-0078-8
44. Atilola O. Level of community mental health literacy in sub-Saharan Africa: Current studies are limited in number, scope, spread, and cognizance of cultural nuances. Nord J Psychiatry 2015;69(2):93–101.
45. Cortina M, Sodha A, Fazel M, Ramchandani PG. Prevalence of Child Mental Health Problems in Sub-Saharan Africa _ Global Health _ JAMA Pediatrics _ The JAMA Network. Arch Pediatr Adolesc Med 2012;166(3):276–81.
46. Zeleke WA, Nichols LM, Wondie Y. Mental Health in Ethiopia: an Exploratory Study of Counseling Alignment with Culture. Int J Adv Couns 2019;41(2):214–29.
47. CSA and UNICEF Ethiopia. Multidimensional Child Deprivation in Ethiopia. 2018;Available from: www.unicef.org/Ethiopia and http://www.csa.gov.et
48. Jorm AF. Mental health literacy. Public knowledge and beliefs about mental disorders - University College London. Br J psychiatry J Ment Sci [Internet] 2000;177:396–401. Available from: http://ucl-primo.hosted.exlibrisgroup.com/primo_library/libweb/action/display.do?tabs=detailsTab&ct=display&fn=search&doc=TN_medline11059991&indx=1&recIds=TN_medline11059991&recIdxs=0&elementId=0&renderMode=poppedOut&displayMode=full&frbrVersion=&dscnt=1&
49. Johnson RL. Pathways to adolescent health: Early intervention. J Adolesc Heal [Internet] 2002;31(6 SUPPL.):240–50. Available from: http://ovidsp.ovid.com/ovidweb.cgi?T=JS&PAGE=reference&D=emed5&NEWS=N&AN=2002445167
50. Lam LT. Mental health literacy and mental health status in adolescents: A population-based survey. Child Adolesc Psychiatry Ment Health 2014;8(1):1–8.
51. Hassen HM, Behera MR, Jena PK, Satpathy SK. Validity and Reliability of the Amharic Version of the HLS-EU-Q47 Survey Questionnaire among Urban School Adolescents and University students in Dire Dawa, Ethiopia. Prepr Res Sq 2020;
52. Kersten P, Czuba K, McPherson K, Dudley M, Elder H, Tauroa R, et al. A systematic review of evidence for the psychometric properties of the Strengths and Difficulties Questionnaire. Int J Behav Dev 2016;40(1):64–75.
53. Mathai J, Anderson P, Bourne A. The Strengths and Difficulties Questionnaire (SDQ) as a screening measure prior to admission to a Child and Adolescent Mental Health Service (CAMHS). Aust e-Journal Adv Ment Heal 2002;1(3):235–46.
54. Heizomi H, Kouzekanani K, Jafarabadi MA, Allahverdipour H. Psychometric properties of the persian version of mental health literacy scale. Int J Womens Health 2020;12:513–20.
55. O’Connor M, Casey L, Clough B. Measuring mental health literacy-a review of scale-based measures. J Ment Heal 2014;23(4):197–204.

Formatted: Justified

56. Campos L, Dias P, Palha F, Duarte A, Veiga E. Development and psychometric properties of a new questionnaire for assessing mental health literacy in young people. Univ Psychol 2016;15(2):61–72.
57. Bale J, Grové C, Costello S. I. Ment Heal Prev [Internet] 2018;12(February):26–35. Available from: https://doi.org/10.1016/j.mhp.2018.09.003
58. Lam LT. Mental health literacy and mental health status in adolescents: a population-based survey. Child Adolesc Psychiatry Ment Health 2014;8(26):1–8.
59. Dias P, Campos L, Almeida H, Palha F. Mental health literacy in young adults: Adaptation and psychometric properties of the mental health literacy questionnaire. Int J Environ Res Public Health 2018;15(7).
60. Wei Y, McGrath PJ, Hayden J, Kutcher S. Mental health literacy measures evaluating knowledge, attitudes and help-seeking: A scoping review. BMC Psychiatry [Internet] 2015;15(1). Available from: http://dx.doi.org/10.1186/s12888-015-0681-9
61. Tomé G, Gaspar de Matos M, Camacho I, Gomes P, Reis M, Branquinho C. Mental health promotion in school context – Validation of the ES ' COOL scale for teachers. J Psychiatry Behav Sci [Internet] 2018;2:(1009). Available from: http://meddocsonline.org/
62. Bjørnsen HN, Eilertsen MEB, Ringdal R, Espnes GA, Moksnes UK. Positive mental health literacy: Development and validation of a measure among Norwegian adolescents. BMC Public Health 2017;17(1):1–10.
63. Reilly MO, Svirydzenka N, Adams S, Dogra N. Review of mental health promotion interventions in schools. Soc Psychiatry Psychiatr Epidemiol [Internet] 2018;53(7):647–62. Available from: http://dx.doi.org/10.1007/s00127-018-1530-1
64. Hoofs H, Jansen NWH, Mohren DCL, Jansen MWJ, Kant IJ. The context dependency of the self-report version of the Strength and Difficulties Questionnaire (SDQ): A cross-sectional study between two administration settings. PLoS One [Internet] 2015;10(4):1–21. Available from: http://dx.doi.org/10.1371/journal.pone.0120930
65. Koskelainen M, Sourander A, Kaljonen A. The Strengths and Difficulties Questionnaire among Finnish school-aged children and adolescents. Eur Child Adolesc Psychiatry 2000;9(4):277–84.
66. De Vries PJ, Davids EL, Mathews C, Aarø LE. Measuring adolescent mental health around the globe: Psychometric properties of the self-report Strengths and Difficulties Questionnaire in South Africa, and comparison with UK, Australian and Chinese data. Epidemiol Psychiatr Sci 2018;27(4):369–80.
67. Hughes TL, Quinn C, Tiberi A, Zeleke WA. Developing a Framework to Increase Access to Mental Health Services for Children With Special Needs in Ethiopia. Front Sociol 2020;5(December):1–6.
68. Renshaw TL. Screening Using the SDQ Total Difficulties Scale: An Analog Test of Three Cutoff Values. J Psychoeduc Assess 2019;37(8):1030–6.
69. Editor S, Kamphaus RW. Contemporary Issues in Psychological Assessment. Springer Berlin Heidelberg; 2015.
70. Carod-Artal FJ. Social determinants of mental health. Glob Ment Heal Prev Promot 2017;(2):33–46.
71. FDREMH. Federal Democratic Republic of Ethiopia Ministry of Health: National Mental Health Strategy (2012/13- 2015/16). Heal Sect Dev Program IV 2010/11 – 2014/15 2011;(October 2010):1–114.
72. Lu W. Child and Adolescent Mental Disorders and Health Care Disparities: Results from the National Survey of Children's Health, 2011– 2012. 2017;28:988–1011.
73. Dachew BA, Bifftu BB, Tiruneh BT, Anlay DZ, Wassie MA, Betts K. Prevalence and determinants of mental distress among university students in Ethiopia : a systematic

Formatted: Justified

review protocol. 2019;8–11.
74. Baker-henningham H, Belfer M, Conti G, Ertem I, Omigbodun O, Rohde LA, et al. Global Mental Health 2 Child and adolescent mental health worldwide : evidence. Lancet [Internet] 2011;378(9801):1515–25. Available from: http://dx.doi.org/10.1016/S0140-6736(11)60827-1
75. Nair S, Ganjiwale J, Kharod N, Varma J, Nimbalkar SM. Epidemiological survey of mental health in adolescent school children of Gujarat, India. BMJ Paediatr Open 2017;1(1):e000139.
76. Kovess V, Carta MG, Pez O, Bitfoi A, Koc C, Goelitz D, et al. The School Children Mental Health in Europe (SCMHE) Project: Design and First Results. Clin Pract Epidemiol Ment Heal 2015;11(1):113–23.
77. Patel V, Kleinman A. Poverty and common mental disorders in developing countries. Bull World Health Organ [Internet] 2003;81(8):609–15. Available from: http://www.ncbi.nlm.nih.gov/pubmed/14576893%0Ahttp://www.pubmedcentral.nih.gov/articlerender.fcgi?artid=PMC2572527
78. Lijuan T, Chao J, Wenming X, Huijuan M, Lianzheng Y, Ming L, et al. Prevalence of Psychiatric Disorders among Children and Adolescents in Northeast China. PLoS One 2014;9(10):e111223.
79. Campbell OLK, Bann D, Patalay P. SSM - Population Health The gender gap in adolescent mental health : A cross-national investigation of 566 , 829 adolescents across 73 countries. SSM - Popul Heal [Internet] 2021;13(December 2020). Available from: https://doi.org/10.1016/j.ssmph.2021.100742
80. Tedstone Doherty D, Kartalova-O'Doherty Y. Gender and self-reported mental health problems: Predictors of help seeking from a general practitioner. Br J Health Psychol 2010;15(1):213–28.
81. Atilola O, Pal Y, Balhara S, Avicenna M. Self-Reported Mental Health Problems Among Adolescents in Developing Countries : Results from an International Pilot. J Dev Behav Pediatr 2013;34(2):129–37.
82. Bjørnsen HN, Espnes GA, Eilertsen MEBEB, Ringdal R, Moksnes UK. The Relationship Between Positive Mental Health Literacy and Mental Well-Being Among Adolescents: Implications for School Health Services. J Sch Nurs [Internet] 2019;35(2):107–16. Available from: http://journals.sagepub.com/doi/10.1177/1059840517732125
83. World Health Organization. WHO Adolescent mental health [Internet]. 2018;Available from: http://www.who.int/news-room/fact-sheets/detail/adolescent-mental-health
84. Belfer ML. Child and adolescent mental disorders: The magnitude of the problem across the globe. J Child Psychol Psychiatry Allied Discip 2008;49(3):226–36.
85. Atilola O, Pal Y, Balhara S, Avicenna M, Singh Balhara YP, Stevanovic D, et al. Self-Reported Mental Health Problems Among Adolescents in Developing Countries. J Dev Behav Pediatr [Internet] 2013;34(2):129–37. Available from: http://content.wkhealth.com/linkback/openurl?sid=WKPTLP:landingpage&an=00004703-201302000-00008
86. Ibrahim N, Mohd Safien A, Siau CS, Shahar S. The Effectiveness of a Depression Literacy Program on Stigma and Mental Help-Seeking Among Adolescents in Malaysia: A Control Group Study With 3-Month Follow-Up. Inq (United States) 2020;57.
87. Bjørnsen HN, Espnes GA, Eilertsen MEB, Ringdal R, Moksnes UK. The Relationship Between Positive Mental Health Literacy and Mental Well-Being Among Adolescents: Implications for School Health Services. J Sch Nurs 2019;35(2):107–16.
88. Lam LT. Mental health literacy and mental health status in adolescents: a population-

Formatted: Justified

based survey. Child Adolesc Psychiatry Ment Health [Internet] 2014;8(26):126–9. Available from: http://www.capmh.com/content/8/1/26
89. Wu C, Liu S, Chang S. Surveys of medical seeking preference , mental health literacy , and attitudes toward mental illness in Taiwan , 1990 e 2000. J Formos Med Assoc [Internet] 2014;113(1):33–41. Available from: http://dx.doi.org/10.1016/j.jfma.2013.03.004
90. Furnham A, Lee V, Kolzeev V. Mental health literacy and borderline personality disorder (BPD): what do the public "make" of those with BPD? Soc Psychiatry Psychiatr Epidemiol 2015;50(2):317–24.
91. Oldfield J, Stevenson A, Ortiz E, Haley B. Promoting or suppressing resilience to mental health outcomes in at risk young people: The role of parental and peer attachment and school connectedness. J Adolesc 2018;64(April 2017):13–22.
92. Presseau J, Mccleary N, Lorencatto F, Patey AM, Grimshaw JM, Francis JJ. Action , actor , context , target , time (AACTT): a framework for specifying behaviour. 2019;1–13.
93. Kan MPH, Fabrigar LR, Fishbein M. Theory of Planned Behavior. 2017;1–8.
94. Kessler RC, M. A, Anthony JC, De Graaf R, Demyttenaere K, Gasque I, et al. Lifetime prevalence and age-of-onset distributions of mental disorders in the World Health Organization' s. World Psychiatry 2007;6:168-176) 2007;6(October):168–76.
95. Wei Y, Mcgrath PJ, Hayden J, Kutcher S, Wei Y, Mcgrath PJ, et al. Measurement properties of mental health literacy tools measuring help-seeking : a systematic review. J Ment Heal [Internet] 2017;0(0):000. Available from: http://dx.doi.org/10.1080/09638237.2016.1276532
96. Cornally N, Mccarthy G. Help-seeking behaviour: A concept analysis. Int J Nurs Pract 2011;17(3):280–8.
97. Bonabi H, Müller M, Ajdacic-Gross V, Eisele J, Rodgers S, Seifritz E, et al. Mental health literacy, attitudes to help seeking, and perceived need as predictors of mental health service use: A longitudinal study. J Nerv Ment Dis 2016;204(4):321–4.

Formatted: Font color: Auto

Formatted: Line spacing: single

Formatted: Space After: 0 pt

Formatted: Justified

Original Research

Mental health issues and the association of mental health literacy among adolescents in urban Ethiopia

Hailemariam Mamo Hassen[1*], Manas Ranjan Behera[2], Deepanajli Behera[2], Ranjit Kumar Dehury[3]

[1]Department of Biological Sciences, Dire Dawa University, Dire Dawa, Ethiopia

[2] School of Public Health, KIIT Deemed to be University, Bhubaneswar, India

[3]School of Management Studies, University of Hyderabad, Telangana, India

* hailemariammamo5@gmail.com

Abstract

Background

Epidemiological evidence about the prevalence of adolescent mental health issues and their association with mental health literacy is crucial for sustained mental health promotion strategies. Adolescence is a critical life stage for mental health promotion. However, evidence is not available among Ethiopian school adolescents. Hence, the present study examined the prevalence of adolescents' mental health issues and their correlation with mental health literacy.

Materials and methods

A cross-sectional study was conducted among adolescents (grades 5-12) in Dire Dawa city, Eastern Ethiopia using multistage random sampling. Data was collected using the Strength and Difficulty Questionnaire, WHO-5 well-being index, and mental health literacy questionnaire**.** SPSS version 25 was used for the descriptive, Chi-square, binary logistic regression, and correlation analyses.

Results

About 15.9-28.4% of adolescents had reported mental health problems: internalizing problems (14.9-28.4%), emotional problems (10.4-63.9%), and peer relationship problems (17.8-25.8%). These mental health problems were significantly greater among adolescents who had either themselves or their family members used psychoactive substances ($p \le 0.05$). Females from upper elementary (5-8 grade) and lower secondary (9-10) grade levels had a higher prevalence of mental health problems (AOR: 2.60 (0.95-7.10, $p<0.05$)). The effect of age, parental education, or employment status was insignificant ($p>0.05$). The prevalence of depression ranged from 18.0-25.5%. Mental health literacy was negatively correlated with total difficulties scores and positively associated with mental well-being scores ($p<0.05$).

Conclusion

The prevalence of adolescents' mental health problems was higher. It implied that promoting mental health literacy could enhance adolescents' positive mental health. Intervention programs should prioritize vulnerable groups and individuals reporting symptoms of mental health difficulties. Future studies should involve qualitative studies and consider effect of other determinants.

1. Introduction

Globally, mental health problems disproportionately affect adolescents in low-income countries and they remain the leading causes of disease burden among children and young people.[1] Knowing about these mental health conditions, their symptoms, and treatments termed as mental health literacy is essential for everyone but is crucial for adolescents.[2] Mental health literacy is defined as understanding and applying information about mental health. [2] In this regard, much has been known about the mental health literacy of adolescents from high-income countries.[3] However, it is still a relatively new concept in Africa.[2] Additionally, studies revealed that access to mental health information or services in Africa is lower due to poverty, lack of education, and cultural stigma around mental illness.[1] Nevertheless, limited evidence in Africa, including Ethiopia, has indicated a growing awareness about the importance of mental health literacy. Despite the challenges associated with poverty, cultural stigma, lack of access to health facilities, and lower education coverage, countries are working to improve mental health literacy.[2]

The Ethiopian Ministry of Health has developed a mental health policy focusing on primary mental healthcare and promoting positive mental health, including a mental health awareness campaign[4]. The present study area is Dire Dawa, one of Ethiopian provinces known for its ethnic and cultural language diversity. A recent study revealed that adolescent mental health literacy from Dire Dawa city was lower, consistent with similar studies reports and showing variability across socio-demographic factors. [5]

Epidemiological evidence about the prevalence of mental health problems and their association with mental health literacy is crucial for sustained mental health promotion strategies, as adolescence is a critical life stage for mental health promotion.[6–10] However, such evidence is rarely available among Ethiopian school adolescents. Adolescent mental

health has piqued the interest of public health professionals due to the unique nature of adolescence.[11] Therefore, adolescence remains a critical life stage for mental health promotion.[11] A popular saying goes, “there is no health without mental health.” [12] With multifaceted burdens, mental health is integrally linked to physical health and well-being.[13] The typical adolescent mental health problems include but are not limited to: substance use/abuse or disorders, depression/mood disorders, anxiety, schizophrenia, stress/neurosis, behavioral disorders, postpartum psychosis, and posttraumatic stress disorder (PTSD). [14,15] Several studies showed that these mental health problems in adulthood occur during adolescence. [14,15]

For multiple reasons, adolescents are among the most important groups for mental health studies. For example, adolescence is key to physical, emotional, and cognitive progress.[11,16,17] Fundamentally, mental, physical, and emotional developmental processes evolve during adolescence. [18] Also public health perspectives emphasizing mental health information during early childhood,[18] would help in later life in managing and preventing mental health problems.[18]

Mental health literacy is essential to preventing mental illnesses, consisting of knowledge about mental health problems and recognizing illnesses with their symptoms.[1,3] Studying the mental health status and its association with the mental health literacy of adolescents is paramount because it helps understand these variables across varied cultural settings.[1] The peak of the onset of mental disorders mainly occurs in the adolescent period and needs urgent attention. [19] In low-resource countries like Ethiopia, risk factors such as cigarette smoking, alcohol consumptions, early co-habitation, unhealthy lifestyles etc are higher among adolescents.[20,21] leading to various mental health problems.[11,16,17] Hence,

epidemiological methods like cross-sectional studies provides empirical evidence about the mental health status and factors that contribute to mental health problems.[22] Early understanding of these evidence contribute for making informed and effective interventions to improve adolescents' mental health. [23]

As a result, there is an increasing demand for adolescents' mental health and well-being outcome measures to inform the public and policymakers regarding individual or service-level health promotion and therapeutic practices. In this regard, the strength difficulties questionnaire (SDQ) and the mental well-being index (WHO-5) have been essential to meet the criteria of valid and reliable measures.

The Strengths and Difficulties Questionnaire (SDQ) and the WHO-5 Well-Being Index are commonly used to assess adolescents' mental health.[24–34] The SDQ is a 25-item self-report questionnaire that assesses five mental health domains: conduct problems, emotional symptoms, peer problems, hyperactivity-inattention, and pro-social behavior. [24,25,27–29] The WHO-5 is a 5-item self-report questionnaire that assesses overall well-being. [26,30–34] Both instruments are good choices for assessing adolescent mental health in low-resource settings because they are brief, relatively inexpensive, and easy to administer, and shown to be reliable and valid in adolescents from low-resource settings have been validated in a variety of settings and translated into multiple languages for low-resource settings including in Ethiopia[35,36] Therefore, the SDQ and the WHO-5 are good choices for assessing adolescent mental health in low-resource settings. In this article, the concept of "normalcy" and "mentally sick" as two separate spheres was considered by taking the categorical approach than the dimensional approach of measuring mental health status. [37,38]

The assessment of mental health issues using the familiar Diagnostic Standard Manual – 5 (DSM-5) and International Classification of Disease (ICD-11) analysis has been published in

previous studies.[37,38] Such an approach is marked by clustering disorders, namely, externalizing and internalizing problems. Instruments with high validity are routinely used to measure the internalizing groups (i.e., depressive, somatic, and anxiety symptoms) and the externalizing groups (substance use, disruptive, and conduct symptoms). Furthermore, the well-being state was examined using the WHO-5 index, with lower scores indicating depression.

The daily suffering of people with mental illness affects family members, mainly children, and adolescent populations.[39] Globally, about 25% of the total population and 10-20% of children and adolescents have mental disorders, of which half of all these mental health problems begin by the age of fourteen and three-fourths by the age of early twenties.[40,41] A worldwide-pooled prevalence meta-analysis on mental disorders from all regions of the world addressing 27 countries showed a prevalence of 13.4% ranging from 11.3 to 15.9 (CI: 95%), which indicates mental disorders is affecting a large proportion of the child and adolescent population.[42] According to studies that used the strengths and difficulties questionnaire (SDQ), around 10.5% (5.8-15%) of adolescents in developing countries had mental health concerns.[28]

The prevalence of mental health problems among adolescents in Africa including Ethiopia, was reportedly higher.[43–47] For instance, a meta-analysis study on adolescents in sub-Saharan Africa, including Ethiopia (N=9713), showed the proportion of adolescents having psychopathology was 14.3% (13.6%-15.0%), and the prevalence of psychological disorders was 19.8% (18.8%-20.7%).[45]

These mental health problems are exacerbated by modifiable factors such as low mental health service seeking, lower mental health service use, stigma, discrimination, and common human rights abuses. These factors are linked to immediate and intermediate mental health

outcomes such as health literacy and behaviour. Mental health literacy is a changeable factor linked to improved psychological outcomes.[6–10] It refers to how well a person understands mental diseases and the associated factors.[6–10] Mental health literacy includes an individual's potential to identify mental distress.[9,48,49] Some qualitative descriptions revealed that mental health difficulties and mental well-being is associated with mental health literacy, despite the lack of quantitative evidence of the correlation between mental health literacy, mental health status, and mental well-being.[30]

There has been a scarcity of available epidemiological evidence about mental health conditions and literacy among Ethiopian school adolescents. There was also a lack of evidence about school adolescents' mental health status and the effect of socio-demographic characteristics in the existing literature. Therefore, the objectives of the study were:

- To assess the prevalence of mental health issues among urban adolescents in Ethiopia
- To evaluate the relationship between mental health literacy and mental health issues and
- To examine the impact of socio-demographic characteristics on mental health issues

2. Materials and methods

2.1. Study design and sampling

The present research was a cross-sectional study among public and private school adolescents in grades 5 through 12 from 11 to 19 years in Dire Dawa, Ethiopia. A combination of multistage (schools, classrooms, then individual students) and systematic and random (using the list of the students in fixed intervals of their roll numbers) was used to select study participants from public and private schools. ~~A multistage random sampling method was used to choose the respondents~~. The prevalence of self-reported mental health difficulty was

obtained from the pilot study of the same population (p=0.378) [51], and the margin of sampling error (d=0.04) was taken to calculate the sample size.

$$n \geq \frac{(Za/2)^2 \text{ x } P(1-P)}{d^2} = \frac{1.96^2 \text{ x } 0.378(1-0.378)}{0.04^2} = 559.673 \approx 560$$

Data was collected from June 2020 to July 2020 using the mental health literacy questionnaire (MHLQ), the Strength and Difficulty Questionnaire (SDQ), and the WHO-5 well-being index after cross-cultural validation in the study settings. Maximum sample size estimation (n= 924) was approached after taking design effect (d=1.5) and a 10% non-response. About 751 participants filled out the questionnaires out of the 924 potential participants.

2.2. Data collection tools

Data collection tools were the Strengths and Difficulties Questionnaire (SDQ), mental health literacy questionnaire (MHLQ), and mental well-being index (WHO-5). The predictor variables mainly focusing on age, grade level, self or any family member experience with psychoactive substance use, parents practicing corporal punishment, perceived worry about family problems, parents education, and job status were collected by using questionnaires. The Strengths and Difficulties Questionnaire (SDQ) is a valid, rapid measure for emotional and behavioral problems worldwide.[24,25,27–29] SDQ is a 25-item, 3-point Likert scale (0=not true, 1=somewhat true, and 2=certainly true). It measures subscale difficulty problems that are emotional(SDQ3,8,13,16&24), conduct (SDQ5,7,12,18&22), hyperactivity/inattention(SDQ 2,10,15,21&25), peer(SDQ6,11,14,19&23) and pro-social behaviour (SDQ 1,4,9,17&20) problems.[52] The total difficulties score combines conduct, hyperactivity, emotional, and peer relationship behaviour problems. The externalizing

problem manifests emotional and peer behavioural relationship problems. Meanwhile, the internalizing problem manifests the conduct and hyperactivity/inattention problems.

Adding up the first four subscales leads to total difficulty scores (the higher the total difficulties score, the more significant the mental health difficulty).[52] The fifth sub-scale of the SDQ reflects pro-social behaviour (the higher the score, the better the pro-social behaviour).[52] SDQ is widely used in resource-poor countries to measure children and adolescents' mental and emotional problems. The baseline for cut-off scores definition of total and subscales strength and difficulty scores were from the United Kingdom population norms defined during the instrument development. [27,53] The self-administered SDQ is a validated and widely used measurement tool for determining mental health problems in children and adolescents.[24,25,27–29] It has already been translated into over 60 languages, including Ethiopian Amharic.[24,25,27–29] It has reportedly been an effective tool for assessing children's mental health in a recent scoping review in Africa. [35]

Mental health literacy was measured using the mental health literacy questionnaire (MHLQ). This MHLQ tool is freely available and validated for adults, [54] and adolescents [7,55–63] living in low-income countries to measure health literacy scores. In the context of the current investigation, its reliability was evaluated using Cronbach's alpha and found to be 0.834. MHLQ has 33 items with five Likert scale ratings *(1=strongly disagree, 2=slightly disagree, 3=neither agree nor disagree, 4=slightly agree, 5=strongly agree).* The MHLQ consists of 33 questions that measure factors like recognition (10 questions), knowledge (8 questions), attitudes (8 questions), and beliefs (7 questions) of the adolescents on mental health status. [6–10] The range of scores for the mental health literacy tool is 33 to 165. A higher score implies a better level of mental health literacy.

The mental well-being index (WHO-5) is an overarching expression of the quality of the various domains in the life of adolescents subjective to their mental and psychological well-being. Mental well-being was assessed using the WHO's well-being index, which consists of 5 items (WHO-5).[26,30–34] It is a Likert-type scale with a five-point scale (5=all of the time, 4=most of the time, 3=more than half the time, 2=less than half the time, 1= some of the time, 0=at no time).

2.3. Ethical approval

Ethical approval was obtained from KIIT University (Reference: KIIT/KIMS /IEC/63/2019) and Haramaya University (Reference: 00. HMS/10.0/3763/2020. Written informed consent was obtained from all participants. Written informed consent was obtained from parents of adolescents aged under fifteen years had offered their consent and adolescents of fifteen and above years were factored out following the school counsellors and school principals adequate explanation about their level of maturity and decisional capacity. As a duly authorized representative, written informed consent was obtained from school principals.

2.4. Statistical analysis

SPSS version 25 was used to carry out the statistical analysis. Descriptive analysis was done to present the socio-demographic characteristics, cut-off score determination, and prevalence of mental health problems. Chi-square test and binary logistic regression analysis were used to estimate the difference and compare the mental health problems prevalence between males and females across some socio-demographic characteristics. Correlation analysis was performed to assess the associations between mental health literacy, strength difficulties questionnaire scale, and subjective mental well-being. Estimates used a confidence interval of 95 % ($p \leq 0.05$).

The cut-off score for mental health problems(cases) was defined using the original 3 band categorizations (cut-off scores at 80th & 90th).[64–66] Scores of every item in the scale are added from 0–10. The added scores (range of 0–40) were calculated by adding scores on the emotion components, conduct problems, hyperactivity, inattention, and peer problem scales.[27–29] The cut-off scores was defined by percentile into the highest first 10% (abnormal), the next 10% (borderline), and the remaining 80% (normal). The cut-off for mental health problems (caseness) was the highest at first 10 %.

3. Results

3.1. The socio-demographic characteristics of study participants

About 751 participants comprising 50.1% males and 49.9% females, completed the questionnaires, and statistical analysis was performed for the 731 participants after removing 2.66% of respondents from the analysis for significant missing data. It did not affect the sample power because a greater number of participants were participated compared to the calculated minimum sample size (n=565). The mean age with standard deviation of male and female respondents was 16.27 ± 2.19 and 15.95 ± 2.02, respectively. The age range for adolescents and grouping was based on UNICEF adolescents' age categorization as early, middle, and late adolescence age groups, which is helpful to target mental health promotion and prevention efforts during these stages to address their respective mental health demands.

3.2. Strength difficulty scores and prevalence of mental health status

The original 3 bands (cut-off scores at 80th & 90th) and the newer 4 band (cut-off scores at 80th, 90th & 95th) categorization were reported just for comparison as reported in **Table 1**. The original 3 band was used to determine and estimate adolescents' mental health issues. The mental health problems expressed with the total difficulties and in subscales, in general, were higher for female adolescents than male adolescents. The prevalence of mental health problems (cases) was presented across gender and age categories.

Table 1: The cut of scores for baseline reference ranges (caseness) for both the original 3 band and the newer 4 band categorizations

Self-completed SDQ	Original 3 band categories(cut-off scores at 80th & 90th)						Newer 4 band categorisations (cut-off scores at 80th, 90th & 95th)							
	Normal		Borderline		Abnormal		Close to average		Slightly raised slightly lowered		High (/low)		Very high (/very low	
	Baseline norm	Present study range	Baseline norm	Present study range	Baseline norm	Present study ran	Baseline norm	Present study ran	Baseline norm	Present study ran	Baseline norm	Present study ran	Baseline norm	Present study range

						ge		ge		ge		ge		
Total difficulties score	0-15	0-14	16-19	15-17	20-40	18-40	0-14	0-14	15-17	15-17	18-19	18-20	20-40	20-40
Emotional problems score	0-5	0-5	6	6	7-10	7-10	0-4	0-5	5	6	6	7	7-10	8-10
Conduct problems score	0-3	0-3	4	4	5-10	5-10	0-3	0-3	4	4	5	5	6-10	6-10
Hyperactivity score	0-5	0-4	6	5	7-10	6-10	0-5	0-4	6	5	7	6	8-10	7-9
Peer problems score	0-3	0-4	4-5	5	6-10	6-10	0-2	0-4	3	5	4	6	5-10	7-9
Pro-social score	6-10	6-10	5	5	0-4	0-4	7-10	7-10	6	6	5	5	0-4	0-4
Externalising	0-5	0-5	6-10	6-7	11-20	8-20	0-5	0-5	6-10	6-7	11-12	8	13-20	9-20

score														
Internalising score	0-4	0-8	5-8	9-10	9-20	11-20	0-4	0-8	5-8	9-10	9-10	11-12	11-20	13-20

As indicated in **Fig 1**, the prevalence of mental health total difficulties (15.9-25.5%), internalising (14.9-28.4%), emotional (10.4-23.9%), and peer relationship (17.8-25.8%) problems were higher compared to reports of previous studies. ~~These mental health problems were more prevalent for female and male adolescents.~~ These mental health problems were more prevalent for female and male adolescents compared to other age groups in extent of percentage as explicitly visualized in Figure 1 across the three age groups. Prevalence of depression ranged from 18.0%-25.5%, reportedly higher for female adolescents aged 14-16 years.

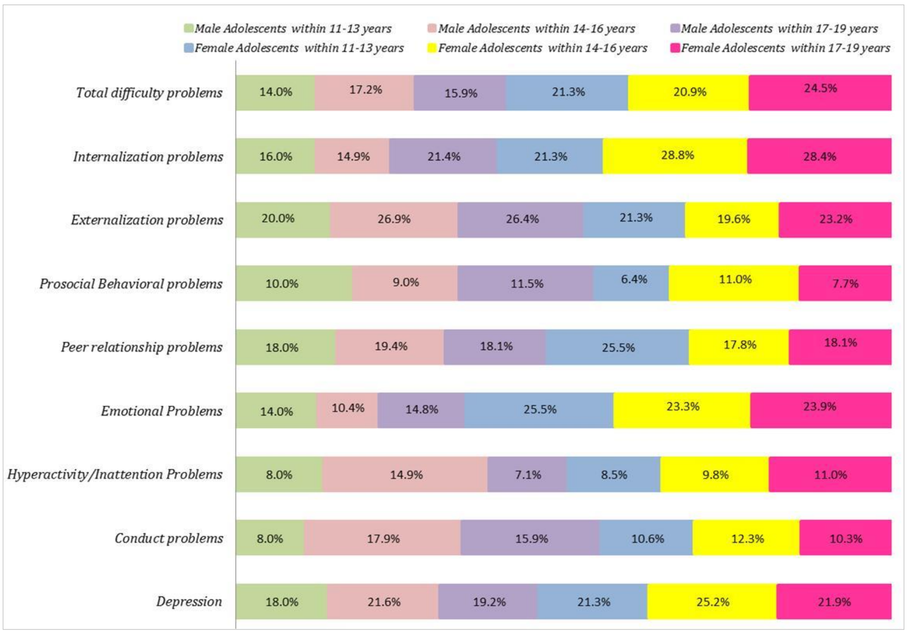

Figure 1: Prevalence of mental health problems among school adolescents in Dire Dawa, Ethiopia.

3.3. Association of mental health status and socio-demographic factors

Statistical difference in prevalence was examined for each banding of subscale using the Chi-square test. The proportion of abnormal females with total difficulties and emotional and internalizing problems was significantly higher ($p<0.05$) than males. The prevalence of conduct and externalizing problems was highly associated with being male. The association of socio-demographic characteristics with mental health status was analyzed using binary logistic regression as reported in **Table 2**.

Table 2: Chi-square test of mental health problems prevalence as per the original 3 band categorization (cut-off scores at 80th & 90th) between males and females

Subscales and bandings	Males (n=366)		Females (n=365)		df	X^2	p
	n	%	n	%			
Total difficulties					2	6.913[a]	0.042
Normal(0-14)	307	83.9%	283	77.5%			
Borderline(15-17)	29	7.9%	48	13.2%			
Abnormal(18-40)	30	8.2%	34	9.3%			
Emotional problems					2	14.442[a]	0.001

score								
Normal0-5)	318	86.9%	278	76.2%				
Borderline=6	23	6.3%	36	9.9%				
Abnormal(7-10)	25	6.8%	51	14.0%				
Conduct problems								
Normal(0-3)	309	84.4%	324	88.8%	2	3.674[a]	0.159	
Borderline=4	30	8.2%	18	4.9%				
Abnormal(5-10)	25.0	7.4%	25.0	6.3%				
Hyperactivity score					2	0.488[a]	0.783	
Normal (0-4)	329	89.9%	328	89.9%				
Borderline=5	21	5.7%	18	4.9%				
Abnormal(6-10)	16	4.4%	19	5.2%				
Peer problems score					2	0.930[a]	0.628	
Normal(0-4)	298	81.4%	296	81.1%				
Borderline=5	41	11.2%	36	9.9%				
Abnormal(6-10)	27	7.4%	33	9.0%				
Internalising score					2	9.332[a]	0.009	
Normal(0-8)	299	81.7%	264	72.3%				
Borderline(9-10)	36	9.8%	50	13.7%				
Abnormal(11-20)	31	8.5%	51	14.0%				
Externalising score					2	1.896[a]	0.387	
Normal(0-5)	272	74.3%	287	78.6%				

Borderline(6-7)	50	13.7%	41	11.2%			
Abnormal(8-20)	44	12.0%	37	10.1%			
Pro-social score					2	0.549[a]	0.760
Normal(6-10)	328	89.6%	332	91.0%			
Borderline=5	18	4.9%	14	3.8%			
Abnormal(0-4)	20	5.5%	19	5.2%			

A binary logistic regression model analysis showed that the odds of mental health problems were significantly associated with some socio-demographic characteristics ($p<0.05$); odds were different across these characteristics as reported in **Table 3**. The prevalence of mental health problems was significantly higher for females in upper elementary (AOR: 2.60 (0.95-7.10)) and lower secondary levels (AOR: 2.73 (1.19-6.29)) compared to upper secondary grade level ($p<0.05$); but it was not significantly different for males. It was twice higher among adolescents with a self and family members experiencing psychoactive substance use ($p<0.05$). Prevalence difference across age, maternal and paternal education, and employment status was insignificant ($p>0.05$).

Table 3: Association of mental health problems and socio-demographic determinants analyzed with binary logistic regression

		Prevalence of mental health problem %			
Predictors		Male		Female	
	Categories	UAOR(95%	AOR(95% CI)	UAOR(95%	AOR(95% CI)

		CI)		CI)	
	11-19	-	-		-
Age group(yrs)	11-13	1	1	1	1
	14-16	1.27(0.51-3.18)	0.959(0.25-3.7)	0.98 (0.44-2.16)	0.65 (0.20-2.06)
	17-19	1.16(0.48-2.84)	0.643(0.06-6.28)	1.20 (0.55-2.64)	0.53 (0.08-3.38)
Grade level	Upper elementary (5-8)	1.08(0.51-2.31)	1.44(0.50-4.15)	1.57 (0.70-3.50)	2.60 (0.95-7.10)
	Lower Secondary (9-10)	1.02(0.48-2.18)	1.11(0.50-2.46)	2.22 (1.00-4.92)*	2.73 (1.19-6.29)*
	Upper secondary (11-12)	1	1	1	1
Experience on psychoactive substances use					
Self-experience	No	1	1	1	1
	Yes	0.45(0.22-0.93*	2.20(1.07-4.52)*	2.17 (0.95-4.95)	2.16 (0.95-

					4.92)
Any family experience	No	1	1	1	1
	Yes	2.01(1.01-4.00)*	2.04(1.01-4.09)*	1.07(0.63-1.84)	1.06 (0.62-1.81)
Parents practicing corporal punishment	No	1	1	1	1
	Yes	1.24 (0.69-2.24)	1.24 (0.69-2.23)	1.43 (0.82-2.51)	1.33 (0.75-2.36)
Adolescents report perceived worry about family problem	No	1	1	1	1
	Yes	1.186(0.64-2.21)	1.17(0.62-2.20)	1.24 (0.73-2.12)	1.20 (0.70-2.06)
Mother education level	Non-educated	1	1	1	1
	Elementary	1.31(0.50-3.43)	1.30 (0.49-3.41)	1.99 (0.57-6.96)	0.73 (0.29-1.87)
	Secondary	1.30(0.66-2.57)	1.30 (0.66-2.58)	1.50 (0.34-6.70)	1.37 (0.78-2.42)
	College or above	0.63(0.18-	0.64 (0.18-	2.71 (0.74-	0.52

		2.20)	2.24)	9.93)	(0.15-1.81)
Father education level	Non-educated	1	1	1	1
	Elementary	1.51 (0.47-4.82)	1.48 (0.46-4.74	1.03 (0.21-5.14)	1.00 (0.20-4.99)
	Secondary	1.60 (0.83-3.09)	1.61 (0.83-3.11	1.04 (0.58-1.84)	1.07 (0.60-1.90)
	college or above	1.42 (0.64-3.16)	1.45 (0.65-3.23	1.23 (0.61-2.46)	1.20 (0.60-2.41)
Mother job	House wife/Unemployed	1	1	1	1
	Work in private	0.79 (0.41-1.51)	0.79 (0.41-1.51)	1.40 (0.77-2.53)	1.40 (0.77-2.54)
	Employed	0.44 (0.16-1.22)	0.44 (0.16-1.21)	0.55 (0.22-1.42)	0.55 (0.21-1.44)
Father job	Unemployed/Jobless	1	1	1	1
	Work in private	1.24 (0.66-2.32)	1.24 (0.66-2.33)	0.75(0.43-1.33)	0.77(0.44-1.36)

	Employed	2.56 (0.75-8.74)	2.52 (0.73-8.70)	ns	ns

** Significant at p<0.01 * Significant at p<0.05

However, the prevalence of mental health problems was not significantly different for males by education level. Prevalence difference was insignificant across the three age groups (11-13, 14-16, and 17-19 years), maternal and paternal education level, and types of jobs or level of employment (p>0.05). The prevalence of mental health problems was twice higher among psychoactive substance users (p<0.05) compared with non-users. Differences in prevalence existed but were insignificant across these three age groups, maternal and paternal education level, and types of jobs or level of employment (p>0.05).

3.4. Correlation of mental health literacy with total difficulty scores and well-being index

The bivariate correlations showed that the mental health literacy score was negatively associated with the total and subscale strength difficulties scores (conduct problems, emotional problems, hyperactivity-inattention, and peer problems) as it is shown in **Table 4**.

Table 4: The correlations between mean mental health literacy score, strength difficulties questionnaire scale, and mental wellbeing index

Variables		Correlation coefficient (r)) with 95%CI								
		1	2	3	4	5	6	7	8	9
1.	SDQ Total difficulties	1	-.31	-.124**	.652**	.700**	.749**	617** (.570-	.825**	.863**

	problems score		9** (-.388-(-.250))	(-.198-(-.052))	(.607-.694)	(.656-.740)	(.714-.783)	.659)	(.798-.848)	(.842-.882)
2.	Mental wellbeing index	-	1	.160** (.084-.234)	-.140** (-.206-(-.076))	-.281** (-.350-(-.210))	-.293** (-.366-(-.215))	-.130** (-.200-(-.060	-.258** (-.329-(-.186))	-.279** (-.347-(-.209))
3.	MHL Total mean score	-	-	1	-.135** (-.211-(-.064))	-.136** (-.212-(-.058))	-0.006 (-.078-.058)	-.088* (-.165-(-.010))	-.166** (-.244-(-.087))	-0.051 (-.120-.016)
4.	SDQ Conduct problems	-	-	-	1	.345** (.275-.412)	.228** (.159-.301)	.294** (.220-.368)	.812** (.785-.837)	.319** (.249-.388)

	subscale mean score									
5.	SDQ Hyperactivity problems subscale mean score	-	-	-	-	1	.385** (.310-.454)	.197** (.125-.265)	.828** (.802-.854)	.380** (.310-.446)
6.	SDQ Emotional problems subscale mean score	-	-	-	-	-	1	.272** (.195-.342)	.376** (.301-.453)	.862** (.844-.880)
7.	SDQ Peer problems subscale mean score	-	-	-	-	-	-	1	.298** (.231-.362)	.721** (.683-.756)
8.	SDQ Externalizing Problems subscale mean score	-	-	-	-	-	-	-	1	.427** (.360-.489)

9.	SDQ Internalizing problems subscale mean score	-	-	-	-	-	-	-	-	1

** Correlation is significant at the 0.01 level (2-tailed); * Correlation is significant at the 0.05 level (2-tailed

4. Discussion

The Ethiopian government has taken several interventions to mitigate the adolescent mental health issue, establishing mental health facilities nationwide.[67] Despite the delayed initiation of the policy and lack of established systems and practices, there has been growing recognition of the importance of adolescent mental health promotion. The Ethiopian government has shown commitment to promoting mental health and addressing mental health issues in schools, for instance, by employing trained school counselors and establishing clubs in schools in secondary schools. The government has also trained health professionals in mental health care who are better equipped to identify and treat mental health problems in adolescents—implementing psycho-education programs in schools, public awareness campaigns, and training for community leaders and media outlets for adolescents, among others.[4] The state mechanism in promoting mental health literacy in Ethiopia is that the Ministry of Health is responsible for developing and implementing mental health policies and programs and provides training for health professionals in mental health care.[4] Stakeholders including professional associations, such as The Ethiopian Mental Health Association and The Ethiopian Psychiatric Association, are involved in policy consultation, provide training and support for health professionals, and promote mental health research in Ethiopia. These policy and programs are cascaded along the government administrative levels for actual implementation. However, there is still a paucity of evidence about the mental health status of adolescents and factors that affect mental health problems and factors that contribute for effective interventions to improve adolescents' mental health.

The SDQ is a screening tool for mental health problems in children and adolescents. It is scored on a scale of 0 to 40, with higher scores indicating more problems. The original cut-

Formatted: Font: (Asian) Chinese (PRC)

off scores for the SDQ are 80 and 90, with scores above 90 being considered abnormal, scores between 80 and 90 being considered borderline, and scores below 80 being considered normal. [27,53] The cut-off score for the present study was slightly lower for total difficulties and subscales for hyperactivity scores than the baseline and relatively higher for internalizing scores (**Table 1**). These cut-off scores for the present study were almost similar to the baseline definition that was taken from the UK child and adolescent population during the first version of the Strengths and Difficulties Questionnaire (SDQ) development. [27,53]

Using cut-off values from local population scores ensures cultural equivalence; that supports relevancy and applicability of the instrument to the cultural groups.[68] As indicated in Table 1, the study showed that the reference scores range taken from a present study for hyperactivity and internalizing scores were higher compared to baseline reference scores ranges. [27,53] The reference scores from a present study using the original 3 band categories (cut-off scores at 80th & 90th) for conduct problems, emotional problems, peer problems, and pro-social scores were similar to the baseline reference scores ranges.[27,53] The reference scores range taken from a present study for total difficulties, hyperactivity, and externalizing scores were lower than baseline reference scores. [27,53] If the cut-off score for a given population is higher than the original cut-off score, it means that a more significant proportion of children in that population are experiencing mental health problems.[69] This could be due to factors such as poverty, social disadvantage, or exposure to trauma.[70]

It is important to note that the SDQ is a screening tool, not a diagnostic tool.[69] If a child scores above the cut-off score, it does not necessarily mean they have a mental health problem.[69] However, it does indicate that they are at an increased risk of having a problem, and further assessment is warranted. Considering these factors is essential when interpreting a lower cut-off score from the baseline. Some evidence indicated that lower cut-off values have

been more effective than the higher cut-off values at identifying child and adolescent mental health problems.[68]

These findings implied that the adolescent population in urban Ethiopia is more disadvantaged than the population on which the original cut-off scores were based. Adolescents in urban Ethiopia have been exposed to stressors like poverty, violence, or trauma. This adolescent population has less access to mental health services and is more likely to underreport mental health problems.

As shown on **Fig. 1,** the present finding was within the same range of the Ethiopian national level prevalence of adolescent mental health problems, reportedly ranging from 17-23%, like other study results in the Ethiopian context.[71–73] Prevalence of total difficulties (15.9-25.5%), internalising (14.9-28.4%), emotional (10.4-23.9%), and peer relationship (17.8-25.8%) was similar that was within the range of global prevalence(10-20%).[74]

Several studies showed discrepancies in adolescent mental health problems prevalence from region to region and country to country.[70,75–77] Similarly, these differences exist across socio-demographic characteristics. [78] From the present study, mental health problems expressed by the total strength difficulties and its subscales of problems were higher among female adolescents than male adolescents. A study among Indian children, the association between the female gender and total strength difficulties score was significant.[75] Being female was associated with emotional problems.[75] A cross-national study across 73 countries on the gender gap in adolescent mental health showed that girls have worse average mental health than boys.[79] The possible explanations include but are not limited to socio-cultural factors, gender stereotypes, trauma, and abuse that disproportionally affect girls. [22,80] In contrast, conduct and hyperactivity/inattention problems were significantly more severe among males than females.[66] Consistent with other studies, the prevalence of conduct

and externalizing problems was more significant among male adolescents than females. For example, a study in Northeast china showed a similar result.[78] A study among children and adolescents from seven European countries (Italy, Netherlands, Germany, Romania, Bulgaria, Lithuania, and Turkey) reported that externalized problems were consistently higher in males than females and reversed for internalized problems.[76] One possible explanation might be that mental health status is shaped by social, economic, and physical environments leading to inequalities that disproportionally affect gender differences and are heavily associated with risk factors for many common mental disorders.[70] According to the WHO, the relationship between the prevalence of mental health problems and poverty indices was statistically significant.[77] These poverty indices are education disparity, low income, a lack of material goods, job, and housing obstacles. For several cultural and traditional reasons, these factors impact females more than boys.[77]

Odds of mental health problems were significantly associated with some socio-demographic characteristics ($p<0.05$) differing in magnitude across these characteristics. In this study, the prevalence of mental health problems was significantly higher for females in upper elementary and lower secondary levels than for upper secondary grade levels ($p<0.05$), consistent with reports of several studies.[70,77] According to WHO report, educational status is one of the major factors determining adolescent mental health outcomes.[77]

However, the difference was insignificant for male adolescents. Furthermore, differences in prevalence have existed but little across the three age groups, maternal and paternal education level, and job types or level of employment ($p>0.05$). The present study's prevalence of mental health problems was twice higher among adolescents with a self and/or family members' experience of psychoactive substance use ($p<0.05$). On the contrary, a study in Northeast China showed that the prevalence of any mental disorders and internalizing disorders was significantly lower in younger adolescents than in elders. Children aged 11–14

years had the highest prevalence of internalizing disorders.[78] It was also inconsistent with a study in developing countries showing that lower maternal education was significantly associated with abnormal total SDQ scores.[81]

The present study revealed mental health literacy was negatively correlated with strength difficulties scores and positively correlated with mental well-being; both were significantly correlated but weaker in magnitude. These findings were consistent with existing theories and the results of previous studies. For instance, a study demonstrated that an inadequate level of mental health literacy was negatively associated with depression. The prevalence of depression was 1.52 times higher in those with levels of inadequate MHL than in those who demonstrated an adequate level of MHL.[50] Another study depicted MHL as an explanatory variable of the mental well-being of adolescents having a positive correlation with it**.** [82]

A possible explanation for the negative association between mental health literacy and strength difficulties scores might be that low mental health literacy contributes to the mental health promotion and prevention gap.[45,83–85] Low mental health literacy exacerbates barriers and difficulties in accessing treatment for mental health problems.[86]

Mental health literacy significantly influences adolescents' perceptions and emotional responses [87], reflecting mental health status and subjective mental well-being. Several studies revealed that mental health literacy had a significant relationship with mental health status. [88–90] A higher level of mental well-being has been reported among adolescents with better know-how about obtaining mental health services.[87] Adolescents' awareness of symptoms, causes, and mental illness treatment contributes to favorable attitudes toward seeking help.[15] Similarly, adolescents with better mental health literacy are less likely to engage in problematic health behaviours and have better help-seeking intention and self-efficacy, contributing to a better mental health and well-being.[91] Mental health literacy is

about the information, attitudes, and awareness of mental health concerns that improve self-efficacy, help-seeking intention, and health behaviour, influencing long-term health outcomes and quality of life. A lack of understanding of the symptoms and nature of mental health disorders and alternate sources of support could explain teenagers' low help-seeking intention and behaviour. [15]

Of course, help-seeking intention and self-efficacy may reflect only one of several mechanisms. Better mental health literacy leads to better mental well-being. Higher help-seeking intention and self-efficacy would only partially mediate the relation between higher mental health literacy and better mental well-being. Components of health behavior expressed in actions, targets, contexts, and time are the most determining factors of mental health outcomes.[92,93]

Mental health literacy influences mental health services and help-seeking for treatment.[94] Hence, mental health literacy is essential to improve and often but not always corresponds to help-seeking behaviours. [95] Anthony F. Jorm and his colleagues revealed that changing knowledge in principle is vital and not problematic; however, changing heartily emotional reactions to practice caring and preventing mental disorders may be much more challenging.[10]

Help-seeking, interchangeably health-seeking, has become one of the essential perspectives in understanding causes and factors for patient delay from appropriate action across different health conditions.[96] Despite the prevalent mental health problems and poor well-being, adolescents' unwillingness and low intention to seek help usually result in delays in timely treatment.[43] Most adolescents with mental health problems are reluctant and miss the use of healthcare for mental health, which increases the complexity of social, mental, and general

health outcomes.[97] The findings from this study implied that promoting mental health literacy can improve subjective mental well-being.[87]

The recently revised policy released by The Ethiopian Ministry of Health in 2017 aimed to improve adolescents' mental health by providing early identification, prevention, and treatment services. [4,21] Following the policy, programs were launched in 2018, including school-based mental health programs, community-based mental health services, and training for health care providers.[21] Empirical evidence on early identification of vulnerable groups, individuals reporting symptoms of mental health difficulties and the associated actors could enhance the impact and effectiveness of these programs implementation.[4,21] In this regard, demographic and socioeconomic sensitive approaches should be applied to mental health policies for mental health promotion and mental illness prevention.

5. Limitations

The study has some inherent limitations. It is not applicable to generalize these findings across the country; because Ethiopia exhibits multicultural and multiethnic diversity and multiple socio-demographic backgrounds. It could not even be generalized to all adolescents of the study area, because the samples were selected from the age group (11-19) and grade level (5-12), adolescents at the age of 10 years and lower grade levels were excluded during the first cycle of elementary schools during school sampling. The present study is conducted on the urban adolescents and did not address rural adolescents. The study was quantitative and lacked the qualitative aspect of how and why these findings were observed.

6. Conclusion

The cut-off scores for the present study were almost similar to the baseline definition of the first version of the Strengths and Difficulties Questionnaire (SDQ) development that was taken from the UK child and adolescent population. However, the strength difficulty cut-off score for total strength difficulty and hyperactivity scores was slightly lower and the cut-off score for internalizing scores was slightly higher. The finding was within the same range as the Ethiopian national level prevalence of adolescent mental health problems, reportedly ranging from 17-23%. The prevalence of total difficulties (15.9-25.5%), internalizing (14.9-28.4%), emotional (10.4-23.9%), and peer relationship problems (17.8-25.8%) were similar that was slightly within the range of the global prevalence(10-20%). These mental health problems were significantly greater among adolescents who had either themselves or their family members used psychoactive substances ($p \leq 0.05$) ($p<0.05$). As depicted from strength difficulties scores, the mental health problems were significantly associated with some socio-demographic characteristics ($p<0.05$) differing in magnitude.

Female adolescents scored higher scores on the total difficulty and its subscales (internalizing and externalizing scores) than males, implying that the prevalence of mental health problems was higher among females than their male counterparts, aligning with previous study findings. These findings highlight the need to improve adolescent mental health literacy in urban Ethiopia to improve mental health status. Gender, grade level, age, maternal and paternal education level, job types, and level of employment are factors that affect adolescents' mental health problems. Hence, promoting adolescent mental health through improving mental health literacy requires congruent approaches considering these socio-demographic factors.

The study showed a correlation between mental health literacy level, the strength difficulty scale, and mental well-being scores. It showed mental health literacy negatively correlated with strength difficulties scores and positively associated with mental well-being. These findings were consistent with existing theories and the results of previous studies. It implied that promoting mental health literacy could enhance adolescents' positive mental health, and priorities should be on vulnerable groups and individuals reporting symptoms of mental health difficulties. Future studies should involve qualitative and include the remaining determinants.

Acknowledgments

The authors would like to thank the survey respondents for their participation and contributions to the study.

Authors' contributions

Hassen, H.M and Behera M.R conceptualized the study design. Hassen, H.M collected, analyzed, and interpreted the data used in this study and was a major contributor to writing the article. Behera, D, Behera, M.R. and Dehury, R. K. assisted in analyzing and interpreting the data and writing the paper. All the authors read, commented, and approved the final article.

References

1. Renwick L, Pedley R, Johnson I, Bell V, Lovell K, Bee P, et al. Mental health literacy in children and adolescents in low- and middle-income countries: a mixed studies systematic review and narrative synthesis. Eur Child Adolesc Psychiatry ~~[Internet]~~

2022;(0123456789). Available from: https://doi.org/10.1007/s00787-022-01997-6
2. Sodi T, Quarshie ENB, Oppong Asante K, Radzilani-Makatu M, Makgahlela M, Nkoana S, et al. Mental health literacy of school-going adolescents in sub-Saharan Africa: a regional systematic review protocol. BMJ Open 2022;12(9):1–7.
3. Kucera M, Tomaskova H, Stodola M, Kagstrom A. A Systematic Review of Mental Health Literacy Measures for Children and Adolescents. Adolesc Res Rev [Internet] 2023;8(3):339–58. Available from: https://doi.org/10.1007/s40894-022-00202-8
4. Ministry of Health Federal Democratic Republic of Ethiopia. Ethiopian National Mental Health Strategy (2020-2025). Fed Minist Heal 2020;2025.
5. Hassen HM, Behera MR, Jena PK, Dewey RS DG. Mental Health Literacy of Adolescents and the Effect of Socio- demographic Characteristics : A Cross-sectional Study. Online J Heal allied Sci [Internet] 2021;2–8. Available from: https://www.ojhas.org/issue80/2021-4-6.html
6. Claire M Kelly, Anthony F Jorm, Anne Marie Wright. Improving mental health literacy as a strategy [Internet]. Med. J. Aust. Med. J. Aust.2007;187(187):25–729. Available from: www.mja.com.au
7. Kelly CM, Jorm AF, Wright A. Improving mental health literacy as a strategy to facilitate early intervention for mental disorders. 2007;187(7):1–5.
8. Jorm AF. Why We Need the Concept of "Mental Health Literacy." Health Commun 2015;30(12):1166–8.
9. Jorm AF, Korten AE, Jacomb PA, Christensen H, Rodgers B, Pollitt P. "Mental health literacy": A survey of the public's ability to recognise mental disorders and their beliefs about the effectiveness of treatment. Med J Aust 1997;166(4):182–6.
10. Jorm A. F. et al. Research on mental health literacy: What we know and what we still need to know. Aust N Z J Psychiatry [Internet] 2006;40(1):3–5. Available from: http://www.embase.com/search/results?subaction=viewrecord&from=export&id=L43337434%5Cnhttp://dx.doi.org/10.1111/j.1440-1614.2006.01734.x%5Cnhttp://sfx.metabib.ch/sfx_locater?sid=EMBASE&issn=00048674&id=doi:10.1111/j.1440-1614.2006.01734.x&atitle=Research+
11. Patton GC, Olsson CA, Skirbekk V, Saffery R, Wlodek ME, Azzopardi PS, et al. Adolescence and the next generation. Nature [Internet] 2018;554(7693):458–66. Available from: http://dx.doi.org/10.1038/nature25759
12. Steel Z, Marnane C, Iranpour C, Chey T, Jackson JW, Patel V, et al. The global prevalence of common mental disorders: A systematic review and meta-analysis 1980-2013. Int J Epidemiol 2014;43(2):476–93.
13. O'Sullivan J and AE. Healthy Lives: Widening The Focus On Mental Health Annual Public Health Report 2015 Camden and Islington. Camden Islingt Annu Public Heal Rep 2015;1–148.
14. David K, Park MJ, Mulye TP. The Mental Health of Adolescents: A National Profile, 2008. San Francisco: Natl. Adolesc. Health Inf. Cent. (NAHIC), Univ. Calif. 2008;
15. Eschenbeck H, Lehner L, Hofmann H, Bauer S, Becker K, Diestelkamp S, et al. The Mental Health of Adolescents: A National children and adolescents with StresSOS using online or face-to-face interventions: study protocol for a randomized controlled trial within the ProHEAD Consortium. Trials [Internet] 2019;20(1):94. Available from: https://trialsjournal.biomedcentral.com/articles/10.1186/s13063-018-3157-7
16. La Maison C, Munhoz TN, Santos IS, Anselmi L, Barros FC, Matijasevich A. Prevalence and risk factors of psychiatric disorders in early adolescence: 2004 Pelotas (Brazil) birth cohort. Soc Psychiatry Psychiatr Epidemiol [Internet] 2018;53(7):685–97. Available from: http://dx.doi.org/10.1007/s00127-018-1516-z
17. Mills KL. Social development in adolescence: brain and behavioural changes

[Internet]. Univ. Coll. London2015;. Available from: https://discovery.ucl.ac.uk/1468865/1/KateMills_Thesis_FINAL_Appendices.pdf
18. Manganello JA. Health literacy and adolescents: A framework and agenda for future research. Health Educ Res 2008;23(5):840–7.
19. Shahraki-Mohammadi A, Panahi S, Ashouri A, Sayarifard A. Important Systemic Factors for Improving Adolescent Mental Health Literacy. Iran J Psychiatry 2023;18(1):45–54.
20. Seboka BT, Hailegebreal S, Negash M, Mamo TT, Ewune HA, Gilano G, et al. Predictors of Mental Health Literacy and Information Seeking Behavior Toward Mental Health Among University Students in Resource-Limited Settings. Int J Gen Med 2022;15(November):8159–72.
21. Hanlon C, Alem A, Lund C, Hailemariam D, Assefa E, Giorgis TW, et al. Moving towards universal health coverage for mental disorders in Ethiopia. Int J Ment Health Syst [Internet] 2019;13(1):1–17. Available from: https://doi.org/10.1186/s13033-019-0268-9
22. Eaton NR, Keyes KM, Krueger RF, Balsis S, Skodol AE, Markon KE, et al. An invariant dimensional liability model of gender differences in mental disorder prevalence: evidence from a national sample. J Abnorm Psychol 2012;121(1):282–8.
23. Fok MSMM, Wong TKSS. What does health literacy mean to children? Contemp nurse a J Aust Nurs Prof 2002;13(2–3):249–58.
24. Goodman R, Ford T, Simmons H, Gatward R, Meltzer H. Using the Strengths and Difficulties Questionnaire (SDQ) to screen for child psychiatric disorders in a community sample. Int Rev Psychiatry 2003;15(1–2):166–72.
25. Skinner D, Sharp C, Marais L, Serekoane M, Lenka M. Assessing the Value of and Contextual and Cultural Acceptability of the Strength and Difficulties Questionnaire (SDQ) in Evaluating Mental Health Problems in HIV/AIDS Affected Children. Int J Ment Health 2014;43(4):76–89.
26. Chongwo E, Ssewanyana D, Nasambu C, Mwangala PN, Mwangi PM, Nyongesa MK, et al. Validation of a Swahili version of the World Health Organization 5-item well-being index among adults living with HIV and epilepsy in rural coastal Kenya. Glob Heal Res Policy 2018;3(1):3–9.
27. Goodman R. The strengths and difficulties questionnaire: A research note. J Child Psychol Psychiatry Allied Discip 1997;38(5):581–6.
28. Atilola O, Singh Balhara YP, Stevanovic D, Avicenna M, Kandemir H. Self-Reported Mental Health Problems Among Adolescents in Developing Countries. J Dev Behav Pediatr [Internet] 2013;34(2):129–37. Available from: http://content.wkhealth.com/linkback/openurl?sid=WKPTLP:landingpage&an=00004703-201302000-00008
29. Goodman A, Lamping DL, Ploubidis GB. When to use broader internalising and externalising subscales instead of the hypothesised five subscales on the strengths and difficulties questionnaire (SDQ): Data from british parents, teachers and children. J Abnorm Child Psychol 2010;38(8):1179–91.
30. Downs A, Boucher LA, Campbell DG, Polyakov A. Using the WHO–5 well-being index to identify college students at risk for mental health problems. J Coll Stud Dev 2017;58(1):113–7.
31. De Wit M, Pouwer F, Gemke RJBJ, Delemarre-Van De Waal HA, Snoek FJ. Validation of the WHO-5 well-being index in adolescents with type 1 diabetes. Diabetes Care 2007;30(8):2003–6.
32. Kusier AO, Folker AP. The Well-Being Index WHO-5: Hedonistic foundation and practical limitations. Med Humanit 2019;1–7.

33. Garland AF, Deyessa N, Desta M, Alem A, Zerihun T, Hall KG, et al. Use of the WHO's perceived Well-Being Index (WHO-5) as an efficient and potentially valid screen for depression in a low income country. Fam Syst Heal 2018;36(2):148–58.
34. Topp CW, Østergaard SD, Søndergaard S, Bech P. 1. The WHO-5 well-being index: A systematic review of the literature. Psychother Psychosom 2015;84(3):167–76.
35. Hoosen N, Davids EL, Vries PJ De, King MS. The Strengths and Difficulties Questionnaire (SDQ) in Africa : a scoping review of its application and validation. Child Adolesc Psychiatry Ment Health [Internet] 2018;1–39. ~~Available from: https://doi.org/10.1186/s13034-017-0212-1~~
36. Hassen HM, Behera MR, Jena PK, Dewey RS. Effectiveness and Implementation Outcome Measures of Mental Health Curriculum Intervention Using Social Media to Improve the Mental Health Literacy of Adolescents. J Multidiscip Healthc ~~[Internet]~~ 2022;15~~(May)~~:979–97. ~~Available from: http://www.ncbi.nlm.nih.gov/pmc/articles/pmc9078434/~~
37. Maher MS and BA. Philosophy and Psychopathology. Springer-Verlag New York Inc.: Springer-Verlag New York Heidelberg Berlin London Paris Tokyo Hong Kong; 1990.
38. Brown TA, Barlow DH. Dimensional Versus Categorical Classification of Mental Disorders in the Fifth Edition of the Diagnostic and Statistical Manual of Mental Disorders and Beyond : Comment on the Special Section. 2005;114(4):551–6.
39. Tusaie KR. Epidemiology of child psychiatric disorders in Addis Ababa, Ethiopia. Division of Child and Adolescent Psychiatry Department of Clinical Sciences; 2013.
40. Ling J, Tay YF, Klainin-Yobas P. Mental health literacy levels. Arch Psychiatr Nurs 2018;
41. World Health Organization. Adolescents: health risks and solutions: https://www.who.int/news-room/fact-sheets/detail/adolescents-health-risks-and-solutions. 2018;
42. Polanczyk G V., Salum GA, Sugaya LS, Caye A, Rohde LA. Annual research review: A meta-analysis of the worldwide prevalence of mental disorders in children and adolescents. J Child Psychol Psychiatry Allied Discip 2015;56(3):345–65.
43. Divin N, Harper P, Curran E, Corry D, Leavey G. Help-Seeking Measures and Their Use in Adolescents: A Systematic Review. Adolesc Res Rev ~~[Internet]~~ 2018;3(1):113–22. ~~Available from:~~ http://dx.doi.org/10.1007/s40894-017-0078-8
44. Atilola O. Level of community mental health literacy in sub-Saharan Africa: Current studies are limited in number, scope, spread, and cognizance of cultural nuances. Nord J Psychiatry 2015;69(2):93–101.
45. Cortina M, Sodha A, Fazel M, Ramchandani PG. Prevalence of Child Mental Health Problems in Sub-Saharan Africa _ Global Health _ JAMA Pediatrics _ The JAMA Network. Arch Pediatr Adolesc Med 2012;166(3):276–81.
46. Zeleke WA, Nichols LM, Wondie Y. Mental Health in Ethiopia: an Exploratory Study of Counseling Alignment with Culture. Int J Adv Couns 2019;41(2):214–29.
47. CSA and UNICEF Ethiopia. Multidimensional Child Deprivation in Ethiopia. 2018;~~Available from:~~ www.unicef.org/Ethiopia and http://www.csa.gov.et
48. Jorm AF. Mental health literacy. Public knowledge and beliefs about mental disorders - University College London. Br J psychiatry J Ment Sci ~~[Internet]~~ 2000;177:396–401. ~~Available from: http://ucl-primo.hosted.exlibrisgroup.com/primo_library/libweb/action/display.do?tabs=detailsTab&ct=display&fn=search&doc=TN_medline11059991&indx=1&recIds=TN_medline11059991&recIdxs=0&elementId=0&renderMode=poppedOut&displayMode=full&frbrVersion=&dscnt=1&~~

49. Johnson RL. Pathways to adolescent health: Early intervention. J Adolesc Heal [Internet] 2002;31(6 SUPPL.):240–50. Available from: http://ovidsp.ovid.com/ovidweb.cgi?T=JS&PAGE=reference&D=emed5&NEWS=N&AN=2002445167
50. Lam LT. Mental health literacy and mental health status in adolescents: A population-based survey. Child Adolesc Psychiatry Ment Health 2014;8(1):1–8.
51. Hassen HM, Behera MR, Jena PK, Satpathy SK. Validity and Reliability of the Amharic Version of the HLS-EU-Q47 Survey Questionnaire among Urban School Adolescents and University students in Dire Dawa, Ethiopia. Prepr Res Sq 2020;
52. Kersten P, Czuba K, McPherson K, Dudley M, Elder H, Tauroa R, et al. A systematic review of evidence for the psychometric properties of the Strengths and Difficulties Questionnaire. Int J Behav Dev 2016;40(1):64–75.
53. Mathai J, Anderson P, Bourne A. The Strengths and Difficulties Questionnaire (SDQ) as a screening measure prior to admission to a Child and Adolescent Mental Health Service (CAMHS). Aust e-Journal Adv Ment Heal 2002;1(3):235–46.
54. Heizomi H, Kouzekanani K, Jafarabadi MA, Allahverdipour H. Psychometric properties of the persian version of mental health literacy scale. Int J Womens Health 2020;12:513–20.
55. O'Connor M, Casey L, Clough B. Measuring mental health literacy-a review of scale-based measures. J Ment Heal 2014;23(4):197–204.
56. Campos L, Dias P, Palha F, Duarte A, Veiga E. Development and psychometric properties of a new questionnaire for assessing mental health literacy in young people. Univ Psychol 2016;15(2):61–72.
57. Bale J, Grové C, Costello S. I. Building a mental health literacy model and verbal scale for children: results of a Delphi study Ment Heal Prev 2018;12:26–35.
58. Lam LT. Mental health literacy and mental health status in adolescents: a population-based survey. Child Adolesc Psychiatry Ment Health 2014;8(26):1–8.
59. Dias P, Campos L, Almeida H, Palha F. Mental health literacy in young adults: Adaptation and psychometric properties of the mental health literacy questionnaire. Int J Environ Res Public Health 2018;15(7).
60. Wei Y, McGrath PJ, Hayden J, Kutcher S. Mental health literacy measures evaluating knowledge, attitudes and help-seeking: A scoping review. BMC Psychiatry [Internet] 2015;15(1). Available from: http://dx.doi.org/10.1186/s12888-015-0681-9
61. Tomé G, Gaspar de Matos M, Camacho I, Gomes P, Reis M, Branquinho C. Mental health promotion in school context – Validation of the ES ' COOL scale for teachers. J Psychiatry Behav Sci [Internet] 2018;2:(1009). Available from: http://meddocsonline.org/
62. Bjørnsen HN, Eilertsen MEB, Ringdal R, Espnes GA, Moksnes UK. Positive mental health literacy: Development and validation of a measure among Norwegian adolescents. BMC Public Health 2017;17(1):1–10.
63. Reilly MO, Svirydzenka N, Adams S, Dogra N. Review of mental health promotion interventions in schools. Soc Psychiatry Psychiatr Epidemiol [Internet] 2018;53(7):647–62. Available from: http://dx.doi.org/10.1007/s00127-018-1530-1
64. Hoofs H, Jansen NWH, Mohren DCL, Jansen MWJ, Kant IJ. The context dependency of the self-report version of the Strength and Difficulties Questionnaire (SDQ): A cross-sectional study between two administration settings. PLoS One [Internet] 2015;10(4):1–21. Available from: http://dx.doi.org/10.1371/journal.pone.0120930
65. Koskelainen M, Sourander A, Kaljonen A. The Strengths and Difficulties Questionnaire among Finnish school-aged children and adolescents. Eur Child Adolesc Psychiatry 2000;9(4):277–84.

Formatted: Font: (Default) Times New Roman, 12 pt, Not Bold, Font color: Custom Color(RGB(50,50,50)), English (India), Check spelling and grammar

66. De Vries PJ, Davids EL, Mathews C, Aarø LE. Measuring adolescent mental health around the globe: Psychometric properties of the self-report Strengths and Difficulties Questionnaire in South Africa, and comparison with UK, Australian and Chinese data. Epidemiol Psychiatr Sci 2018;27(4):369–80.
67. Hughes TL, Quinn C, Tiberi A, Zeleke WA. Developing a Framework to Increase Access to Mental Health Services for Children With Special Needs in Ethiopia. Front Sociol 2020;5(December):1–6.
68. Renshaw TL. Screening Using the SDQ Total Difficulties Scale: An Analog Test of Three Cutoff Values. J Psychoeduc Assess 2019;37(8):1030–6.
69. Editor S, Kamphaus RW. Contemporary Issues in Psychological Assessment. Springer Berlin Heidelberg; 2015.
70. Carod-Artal FJ. Social determinants of mental health. Glob Ment Heal Prev Promot 2017;(2):33–46.
71. FDREMH. Federal Democratic Republic of Ethiopia Ministry of Health: National Mental Health Strategy (2012/13- 2015/16). Heal Sect Dev Program IV 2010/11 – 2014/15 2011;(October 2010):1–114.
72. Lu W. Child and Adolescent Mental Disorders and Health Care Disparities: Results from the National Survey of Children's Health, 2011– 2012. 2017;28:988–1011.
73. Dachew BA, Bifftu BB, Tiruneh BT, Anlay DZ, Wassie MA, Betts K. Prevalence and determinants of mental distress among university students in Ethiopia : a systematic review protocol. 2019;8–11.
74. Baker-henningham H, Belfer M, Conti G, Ertem I, Omigbodun O, Rohde LA, et al. Global Mental Health 2 Child and adolescent mental health worldwide : evidence. Lancet [Internet] 2011;378(9801):1515–25. Available from: http://dx.doi.org/10.1016/S0140-6736(11)60827-1
75. Nair S, Ganjiwale J, Kharod N, Varma J, Nimbalkar SM. Epidemiological survey of mental health in adolescent school children of Gujarat, India. BMJ Paediatr Open 2017;1(1):e000139.
76. Kovess V, Carta MG, Pez O, Bitfoi A, Koc C, Goelitz D, et al. The School Children Mental Health in Europe (SCMHE) Project: Design and First Results. Clin Pract Epidemiol Ment Heal 2015;11(1):113–23.
77. Patel V, Kleinman A. Poverty and common mental disorders in developing countries. Bull World Health Organ [Internet] 2003;81(8):609–15. Available from: http://www.ncbi.nlm.nih.gov/pubmed/14576893%0Ahttp://www.pubmedcentral.nih.gov/articlerender.fcgi?artid=PMC2572527
78. Lijuan T, Chao J, Wenming X, Huijuan M, Lianzheng Y, Ming L, et al. Prevalence of Psychiatric Disorders among Children and Adolescents in Northeast China. PLoS One 2014;9(10):e111223.
79. Campbell OLK, Bann D, Patalay P. SSM - Population Health The gender gap in adolescent mental health : A cross-national investigation of 566 , 829 adolescents across 73 countries. SSM - Popul Heal [Internet] 2021;13(December 2020). Available from: https://doi.org/10.1016/j.ssmph.2021.100742
80. Tedstone Doherty D, Kartalova-O'Doherty Y. Gender and self-reported mental health problems: Predictors of help seeking from a general practitioner. Br J Health Psychol 2010;15(1):213–28.
81. Atilola O, Pal Y, Balhara S, Avicenna M. Self-Reported Mental Health Problems Among Adolescents in Developing Countries : Results from an International Pilot. J Dev Behav Pediatr 2013;34(2):129–37.
82. Bjørnsen HN, Espnes GA, Eilertsen MEBEB, Ringdal R, Moksnes UK. The Relationship Between Positive Mental Health Literacy and Mental Well-Being Among

Adolescents: Implications for School Health Services. J Sch Nurs [Internet] 2019;35(2):107–16. Available from: http://journals.sagepub.com/doi/10.1177/1059840517732125
83. World Health Organization. WHO Adolescent mental health [Internet]. 2018;Available from: http://www.who.int/news-room/fact-sheets/detail/adolescent-mental-health
84. Belfer ML. Child and adolescent mental disorders: The magnitude of the problem across the globe. J Child Psychol Psychiatry Allied Discip 2008;49(3):226–36.
85. Atilola O, Pal Y, Balhara S, Avicenna M, Singh Balhara YP, Stevanovic D, et al. Self-Reported Mental Health Problems Among Adolescents in Developing Countries. J Dev Behav Pediatr [Internet] 2013;34(2):129–37. Available from: http://content.wkhealth.com/linkback/openurl?sid=WKPTLP:landingpage&an=00004703-201302000-00008
86. Ibrahim N, Mohd Safien A, Siau CS, Shahar S. The Effectiveness of a Depression Literacy Program on Stigma and Mental Help-Seeking Among Adolescents in Malaysia: A Control Group Study With 3-Month Follow-Up. Inq (United States) 2020;57.
87. Bjørnsen HN, Espnes GA, Eilertsen MEB, Ringdal R, Moksnes UK. The Relationship Between Positive Mental Health Literacy and Mental Well-Being Among Adolescents: Implications for School Health Services. J Sch Nurs 2019;35(2):107–16.
88. Lam LT. Mental health literacy and mental health status in adolescents: a population-based survey. Child Adolesc Psychiatry Ment Health [Internet] 2014;8(26):126–9. Available from: http://www.capmh.com/content/8/1/26
89. Wu C, Liu S, Chang S. Surveys of medical seeking preference , mental health literacy , and attitudes toward mental illness in Taiwan , 1990 e 2000. J Formos Med Assoc [Internet] 2014;113(1):33–41. Available from: http://dx.doi.org/10.1016/j.jfma.2013.03.004
90. Furnham A, Lee V, Kolzeev V. Mental health literacy and borderline personality disorder (BPD): what do the public "make" of those with BPD? Soc Psychiatry Psychiatr Epidemiol 2015;50(2):317–24.
91. Oldfield J, Stevenson A, Ortiz E, Haley B. Promoting or suppressing resilience to mental health outcomes in at risk young people: The role of parental and peer attachment and school connectedness. J Adolesc 2018;64:(April 2017):13–22.
92. Presseau J, Mccleary N, Lorencatto F, Patey AM, Grimshaw JM, Francis JJ. Action , actor , context , target , time (AACTT): a framework for specifying behaviour. 2019;1–13.
93. Kan MPH, Fabrigar LR, Fishbein M. Theory of Planned Behavior. 2017;1–8.
94. Kessler RC, M. A, Anthony JC, De Graaf R, Demyttenaere K, Gasque I, et al. Lifetime prevalence and age-of-onset distributions of mental disorders in the World Health Organization' s. World Psychiatry 2007;6:168-176) 2007;6(October):168–76.
95. Wei Y, Mcgrath PJ, Hayden J, Kutcher S, Wei Y, Mcgrath PJ, et al. Measurement properties of mental health literacy tools measuring help-seeking : a systematic review. J Ment Heal [Internet] 2017;0(0):000. Available from: http://dx.doi.org/10.1080/09638237.2016.1276532
96. Cornally N, Mccarthy G. Help-seeking behaviour: A concept analysis. Int J Nurs Pract 2011;17(3):280–8.
97. Bonabi H, Müller M, Ajdacic-Gross V, Eisele J, Rodgers S, Seifritz E, et al. Mental health literacy, attitudes to help seeking, and perceived need as predictors of mental health service use: A longitudinal study. J Nerv Ment Dis 2016;204(4):321–4.

Response to Reviewers

Title: Mental health issues and the association of mental health literacy among adolescents in urban Ethiopia (PONE-D-23-13300)

Dear Editor,

We thank you for your and the reviewers' constructive feedback and the opportunity to revise and resubmit our manuscript. We carefully considered the comments, and the manuscript was revised. We appreciate and thank you very much for your feedback on the suggestion. We followed PLOS ONE's style requirements and moved the ethics statement into the Methods section of the manuscript. We referred to Figure 1 and the tables in the text as part of the main manuscript. We reviewed the reference list to ensure it is complete and correct to check for errors. We have addressed the feedback accordingly.

Three separate papers are submitted to the system: one has a marked-up copy of the manuscript that highlights changes with track changes, an unmarked version of the revised paper without tracked changes, and a rebuttal letter for each point raised by the academic Editor and reviewers. We are confident that the updated version is appropriate for publishing and anticipate hearing from you soon.

With regards,
Hailemariam Mamo Hassen (PhD)
On behalf of the authors.

Point-by-point response for Reviewers' comments

Dear reviewers, we are grateful for the feedback and constructive comments helpful for improving the manuscript quality.

Reviewer #1 Comments

Reviewer comment: *The article is quite an important work that can be a valuable addition to the literature on mental health, especially in the Ethiopian region. However, the article needs minor revision before being accepted for publication. The comments are addressed below.*

Author's response: Thank you. We appreciate your constructive comments and agree on that, and *the comments are addressed below.*

Reviewer comment: The precise location of the focus of the study is better mentioned in the abstract to maintain transparent claims and ground of study.
Author's response: Thank you very much, dear reviewer. We mentioned the precise location of the focus of the study in the abstract on page 2, line #1, *that the study was conducted in Dire Dawa City, Eastern Ethiopia. The details are also already presented in the main body of the paper.*

Reviewer comment: *The article needs justification for the themes and methods used. For example, it is always better to connect adolescents'' mental health with life span development and justify the argument in such a line of thought.*
Authors' response: Thank you for your comment**.** We have added more justifications for the choice of themes and methods used to connect adolescents" mental health with life span development. You may kindly refer to the revised manuscript (**Page 4)**

Reviewer comment: *Similarly, more justification is needed in the choice of SDQ and WHO-5 to strengthen the method section.*
Authors' response: Once again, we appreciate your concern. We justified the choice of SDQ, WHO-5, for the measurement of mental health status in the method section. You may kindly refer to the revised manuscript (**Page 5)**

Reviewer comment: In the discussion section, the claim "the cut-off score ... for internalizing scores" needs expanded rationalization and justification to make it more relevant to the article's purpose.
Authors' response: Thank you, dear reviewer. We explained the claim and rationalization/justification on "the cut-off score ... for internalizing scores" and other subscales in the discussion section; you may kindly refer to the revised manuscript (**Page 23)**

Reviewer comment: Page 8, third paragraph last sentence - "A cross-sectional study across 73 countries..." p.11 conclusion statement "Female adolescents ..." need to strengthen rationalization for what it implies for this study.
Authors' response: Thank you so much for your important concern. *We Strengthened rationalization for what it implies for this study for the sentences on:*

- On the revised manuscript, page 24, fourth paragraph - "A cross-sectional study across 73 countries..."(comment incorporated on page line in the revised version. You may kindly refer to the revised manuscript (**Page 24)**

- On the revised manuscript page 29, second paragraph conclusion statement You may kindly refer to the revised manuscript (**Page 29)**

Reviewer comment: More focus is needed on following the prescribed reference style accurately and consistently for in-text citation (P.10) and reference lists (Ref 4,5, 12,16).
Authors' response: We appreciate your concern. We checked and revised all the references, including these commented references, as per the PLOS ONE reference style for its accuracy and consistency, including the commented references in-text citation (P.10) and reference list (Ref 4,5, 12,16)
Reviewer comment: Overall, it is a good work to be accepted for publication with these minor revisions.
Authors' response: Thank you very much.

Reviewer #2 Comments:

Authors' response to Reviewer #2 Comments:

Dear reviewer #2, we acknowledge all insightful comments and fully agree with the suggestions. We addressed the comments one by one.
Reviewer comment: The introduction provides an exhaustive literature review on mental health issues. When explaining a scenario, start with the global scenario followed by Africa, Ethiopia, and mental health literacy in the study area.
Authors' response: Thank you very much, dear reviewer. We added more explanations, starting with the global scenario followed by Africa, Ethiopia, and mental health literacy in the study area. You may kindly refer to the revised manuscript (**Introduction Page 3, first paragraph)**

Reviewer comment: What is the government intervention to mitigate the issue? What are the state mechanisms promoting mental health literacy in Ethiopia? The author can discuss these interventions in discussion sections.
Authors' response: Thank you so much for your essential concern. We discussed the government intervention to mitigate the issue, state mechanisms promoting mental health literacy in Ethiopia, and their policy and programme related to mental health for a more comprehensive understanding in the discussion sections with additional references to the national health policy and programme. You may kindly refer to the revised manuscript (throughout the discussion and mainly in the first paragraph of the discussion part, **Page 22)**

Reviewer comment: The author should discuss their policy and programme related to mental health for a more comprehensive understanding.

Authors' response: We appreciate your crucial concern. *We* discuss the policy and programme related to mental health in Ethiopia throughout the manuscript for a more comprehensive understanding and, most importantly, discussion in the last paragraph of the discussion. (Pages 27-28)

Reviewer comment: The author should highlight the key inference of the present study, how this study enhances the knowledge of mental health literacy among young adolescents in Ethiopia.

Authors' response: Thank you so much. We revised and highlighted the key inference of the study, noting the limitations of generalization in the limitation and conclusion parts (**pages 28&29**).

Reviewer comment: Suggest a few major findings, how the demographic and socioeconomic factors are leading in addressing the current mental health literacy issues in Ethiopia.

Authors' response: Thank you, dear reviewer. We revised the manuscript, presenting the significant findings and how the demographic and socioeconomic factors imply addressing the current mental health literacy (Page 29 paragraph 2)

Reviewer comment: *Is it applicable across Ethiopia, irrespective of all ethnic groups?*

Authors' response: We appreciate your crucial concern. As it is presented in the limitation section, *It is not applicable to generalize these findings across the country because Ethiopia exhibits multicultural and multiethnic diversity and multiple socio-demographic backgrounds. It could not even be generalized to all adolescents of the study area*. (Page 28, limitation, paragraph 1)

Response to Reviewers

Title: Mental health issues and the association of mental health literacy among adolescents in urban Ethiopia (PONE-D-23-13300)

Dear Editor,

We thank you for your and the reviewers' constructive feedback and the opportunity to revise and resubmit our manuscript. We carefully considered each point raised by the academic editor and reviewers and revised the manuscript. We appreciate and thank you very much for the feedbacks and comments. We followed PLOS ONE's style requirements.

Point raised by the academic editor were regarding whether consent obtained from parents or guardians of the minors, ethics approval letter from two institutions of which one was missing and discrepancy in date difference for secretary and chair person for ethics approval letter obtained from Haramaya University and concern to ensure completeness and correctness of references.

Dear Academic editor, regarding consent from parents or guardians of the minors, written informed consent was obtained from parents of adolescents aged under fifteen years had offered their consent and adolescents of fifteen and above years were factored out following the school counsellors and school principals adequate explanation about their level of maturity and decisional capacity ((page 10) with due reference of the guideline International Ethical Guidelines for Health-related Research Involving Humans: Prepared by the Council for International Organizations of Medical Sciences (CIOMS) in collaboration with the World Health Organization (WHO,2016). However, respective school principals as a duly authorized representative had expressed their consent both orally and signed on consent forms for all participants.

Regarding the ethics approval letters, ethics approval letter from the two institutions (KIIT University and Haramaya University) are merged and attached in this revised submission. The discrepancy of the date of signature from the Chairperson (dated 11/02/2018) and Secretary (dated 11/02/2020) on same document was confirmed as typo while the typist was writing and the letter issued unnoticed. We understand that it requires a correction. However, our application for correction from the Institutional review board of Haramaya University failed because the Institutional review board has

restructured and the Signers were changed by other individuals. We sincerely request your esteemed editor to understand that this discrepancy is typo while the typist was writing and we kindly request you recognize this issue.

We reviewed the reference lists to ensure it is complete and correct.

Dear editor, we appreciate the comments raised by the reviewers and we accommodate each comment one by one in the manuscript indicated by track change.

Three separate papers are submitted to the system: one has a marked-up copy of the manuscript that highlights changes with track changes, an unmarked version of the revised paper without tracked changes, and a rebuttal letter for each point raised by the academic Editor and reviewers. We are confident that the updated version is appropriate for publishing and anticipate hearing from you soon.

With regards,
Hailemariam Mamo Hassen (PhD), (First and corresponding author)
On behalf of the authors.

Point-by-point response for Reviewers' comments

Dear reviewers, we are grateful for the feedback and constructive comments helpful for improving the manuscript quality.

Reviewer #2 Comments

Thank you very much, dear reviewers.

Reviewer #2 commented that we authors have addressed all the comments and suggestions stating that mental health literacy is an emerging issue which demand more attention for overall health and well-being.

Authors' response Thank you very much***:***

Reviewer #3 Comments

Author's response: Thank you. We appreciate your constructive comments and agree on *the comments and addressed accordingly.* .

Reviewer #3 comment: Would you please review your claims on the discussion section regarding government actions taken at school level with regard to mental health promotion and prevention as there are no promising activities? These are actually policy promises than actual practices.

Authors' response: Dear Reviewer, thank you so much for your essential concern. We agree that the government actions taken at school level with regard to mental health promotion and prevention are policy promises than actual practices. Accordingly, we discussed these points. *Despite the delayed initiation of the policy and lack of established systems and practices, there has been growing recognition of the importance of adolescent mental health promotion. The Ethiopian government has shown commitment to promoting mental health and addressing mental health issues in schools, for instance, by employing trained school counselors and establishing clubs in schools in secondary schools.* (stated on page 25)

Reviewer #3 comment: I think you better consider including the predictor variables in the method part.

Author's response: Thank you very much, dear reviewer. We included the predictor variables briefly in the method part (page 8). "The predictor variables mainly focusing on age, grade level, self or any family member experience with psychoactive substance use, parents practicing corporal punishment, perceived worry about family problems, parents education, and job status were collected by using questionnaires."

Reviewer #3 comment: Your sampling strategy should discuss the clusters selected all the stages precisely.

Author's response: Thank you. We mentioned the sampling strategy briefly in the method part (page 8). "A combination of multistage (schools, classrooms, then individual students) and systematic and random (using the list of the students in fixed intervals of their roll numbers) was used to select study participants from public and private schools."

Reviewer #3 comment: The statement on page 13 that begins with "These mental health problems were..." does not provide a clear comparison.

Author's response: Once again, we appreciate your concern. We have compared and indicated in the figure 1 in the revised manuscript, page 14. We stated that **"**These mental health problems were more prevalent for female and male adolescents compared to other age groups in extent of percentage as explicitly visualized in Figure 1 across the three age groups. Prevalence of depression ranged from 18.0%-25.5%, reportedly higher for female adolescents aged 14-16 years (page 14).

Reviewer #3 comment: Would you please mention any bases for your age categorization and its subsequent implication for mental health promotion and prevention?

Author's response: Thank you very much, dear reviewer. We already mentioned the bases for your age categorization and its subsequent implication on page 11 of the last 4 lines #6-9; "*The age range for adolescents and grouping was based on UNICEF adolescents' age categorization as early, middle, and late adolescence age groups, which is helpful to target mental health promotion and prevention efforts during these stages to address their respective mental health demands*."
