# Supplementary material for: Mental health issues and the association of mental health literacy among adolescents in urban Ethiopia
Source: PLoS One. 2024 Oct 24;19(10):e0295545. doi: 10.1371/journal.pone.0295545 (PMC11500858; doi:10.1371/journal.pone.0295545)
Supplement: Attachment — Submitted filename: Point-by-point response_file .pdf [file pone.0295545.s005.pdf]

A rebuttal letter

Dear Editor and Reviewers,

Thank you for your valuable feedback on our manuscript "Mental health issues and the association of mental health literacy among adolescents in urban Ethiopia (PONE-D-23-13300)". We are pleased to learn that all reviewers are satisfied with the responses provided to their initial comments. We believe the revised manuscript now fully meets PLOS ONE's publication criteria. For the current version the manuscript, we have carefully considered all of the comments and have made required revisions to address your concerns. All comments have been addressed one by one. The comments raised by the editor and reviewers and authors responses are presented in tabular form for easy reference on the comments and responses. The A marked-up copy of the manuscript with Track Changes is attached for your review.

We believe that the revised manuscript now addresses all of the reviewers' comments and is suitable for publication in PLOS ONE. Thank you again for your time and consideration.

Sincerely,

Hailemariam Mamo Hassen(Ph.D)

Corresponding author

Comments by the editor and reviewers and Authors response

**Comments**	**Authors responses**
**Editor comment**	
Please review your reference list to ensure that it is complete and correct. If you have cited papers that have been retracted, please include the rationale for doing so in the manuscript text, or remove these references and replace them with relevant current references. Any changes to the reference list should be mentioned in the rebuttal letter that accompanies your revised manuscript. If you need to cite a retracted article, indicate the article's retracted status in the References list and also include a citation and full reference for the retraction notice.	**Thank you for your careful review of our manuscript.**<br>We have thoroughly reviewed our reference list to ensure its completeness and accuracy. There are no any retracted references papers.
We request that you make some minor revisions. Please respond to each point raised in the attached reviews and indicate the changes made in your revised manuscript.	Thank you for your prompt review of our manuscript. We appreciate the thoughtful comments from the editor and the reviewers and have carefully considered each point raised incorporated the suggested revisions into the revised manuscript attached. Please find below a detailed response to each of the reviewer's comments:

Reviewers Comments	
Reviewer #2 Comments: *All comments have been addressed. The literature review is meticulously organized, establishing a strong basis for the research. The technique is thoroughly clarified, ensuring clarity and the capacity to replicate the study. The study is rigorously supported by suitable statistical methodologies, and the findings are clearly presented and explained.*<br>*All the best!*	We would like to express our sincere gratitude. Thank you for your valuable feedback.
Reviewer #4 Comments: *Thank you author(s) for your valuable research. Because mental health concerns is crucial for policy makers & any concerned bodies of the intended areas especially regarding adolescents.*<br><br>***My concern in general is that;*** *you indicated that the policy makers in the area were you did your research more or less on striving to strengthen the strategy to tackle mental health problems. Thus it is not your concern to mention it in detailed.*	**Thank you** for your thoughtful comments and appreciation of our research. We acknowledge that mental health concerns are indeed critical for policymakers and relevant stakeholders, particularly among adolescents.<br><br>We understand your concern regarding the level of detail provided on the policy makers' efforts to strengthen mental health strategies.<br>We believe that it's important to acknowledge the broader context within which our research is situated. Statements on policy makers and strategies were mentioned regarding a paucity of evidence about the increasing demand for adolescents' mental health and well-being outcome measures to inform the public and policymakers and professionals involved in policy consultation, and how our study contribute evidences mentioning the delayed initiation of the policy and lack of established systems and practices despite government growing recognition and commitment to promoting mental health. Additionally, we mentioned the existing recently drafted policy released by The Ethiopian Ministry of Health in 2017 aimed to improve adolescents' mental health by providing early identification, prevention, and treatment services. [4,21] and programs launched in 2018, including school-based mental health programs, community-based mental health services, and training for health care providers.[21]<br>Therefore, we kindly note that presence of the evidence statement would be essential.<br>Thank you.
**Reviewer #5 Comments:**	
The authors have undertaken important work and the conclusions could make far reaching contributions to policy. However, I do not believe that adequate rigour has been applied in the analysis and write-up, and the manuscript needs to be edited for grammar and readability. The specific comments will be found in the attached copy of the manuscript. Please find a few key points below:	Thank you for your valuable feedback. We have carefully addressed your specific comments and made significant improvements to our manuscript. We've strengthened our methodology, clarified our writing, and refined our conclusions to better reflect our findings and the policy implications. We believe these revisions have enhanced the quality of our work and hope it meets your journal's high standards.<br>Hence, please find below the detailed responses to each of the comments (a-d).

a. **Reviewer #5 Comments:** *Please be specific where you can be. Words like "about" when talking about your own data suggest that you are not sure of the data you are presenting.*	Thank you for your careful review of our manuscript. We appreciate your attention to detail and your suggestion to replace the word "about" where appropriate. We have carefully reviewed our manuscript and have made the necessary changes to provide more precise and accurate information. We have replaced "about" with specific terms where appropriate (**page 2 line27**), ensuring that our data is presented clearly and accurately. Additionally, we have provided exact numbers or percentages whenever possible to enhance the precision of our reporting.<br>We believe that these revisions have strengthened the clarity and accuracy of our manuscript and have addressed your concerns effectively. Thank you again for your valuable feedback.
b. **Reviewer #5 Comments:** *Avoid extraneous details in the discussion. There was too much written about the study tool in the discussion that should be cut or moved to the methods section*	Thank you very much. Details were presented about the SDQ to the methods section. As the given comment we provided a brief overview in the discussion. We have streamlined the discussion and focused on the key findings and implications of our study removing the *extraneous details*(**page23 line 432-442)**
c. **Reviewer #5 Comments:** *It is curious that a multivariable analysis was not done to test the independence of the observed association between mental health literacy and wellbeing and mental health problems, when several variables that were in your dataset (like age and proxies for socioeconomic status) could easily be confounders. I strongly recommend performing this analysis.*	Thank you for your valuable feedback regarding the inclusion of a multivariable analysis in our study. We understand your recommendation and acknowledge the potential benefits of such an analysis in controlling for confounding variables.<br>While we agree that a multivariable analysis could provide additional insights into the relationship between mental health literacy, well-being, and mental health problems, we believe that the extensive number of tables required for such an analysis would exceed the limitations imposed by PLOS ONE.<br>However, we have carefully considered your suggestion and plan to include a multivariable analysis in a future study with a larger sample size. This will allow us to more comprehensively explore the complex relationships between these variables and provide a deeper understanding of the factors influencing adolescent mental health.<br>We include recommendation that reads *"Future studies with larger sample sizes should conduct multivariable analyses to further explore the complex relationships between mental health literacy, well-being, and mental health problems while controlling for potential confounding factors"* ***(Page29 Line 637-640)***<br>We believe that the correlation analysis presented in this study provides a valuable foundation for future research and contributes significantly to our understanding of the topic. Thank you again for your insightful comments.
d. **Reviewer #5 Comments:** *I recommend that the authors seek independent editing of the manuscript.*	Thank you for your valuable feedback and recommendation for independent editing. We have carefully considered your suggestion and have taken steps to enhance the clarity and coherence of our manuscript.<br>In addition to the independent editing conducted by a colleague, all authors have engaged in a thorough review and revision process. We have carefully examined the discussion section and have made necessary adjustments to ensure that our statements are clear, concise, and supported by the evidence presented in the study.<br>We believe that these combined efforts have significantly improved the overall quality and readability of our manuscript. We are confident that our revised work meets the high standards of your journal.<br>Thank you again for your time and consideration. We look forward to your further feedback.

**Reviewer #6 Comments:**	
Thank you for the opportunity to review this work. The manuscript titled under review present important evidence for adolescent mental health in Africa and by extension low-middle income countries. It applied sound analytical approaches to answer the object of the study. However, the manuscript requires revisions before being accepted for publications.<br>*Kindly find attached my review comments.*	Thank you for your valuable feedback on our manuscript. We appreciate your constructive comments and have carefully considered your comments. We have carefully addressed each of your specific comments and have provided detailed explanations for the changes we have made. Please refer to the revised manuscript for a comprehensive overview of these revisions.
Reviewer #6 Comments on Introduction section	
a. ***Comment1*** *Page 5 paragraph 3: The description and justification of the SDQ and WHO wellbeing 5 should be included in the methods section when discussing the instruments used.*	Thank you for your valuable feedback. We have carefully considered your comment regarding the placement of the description and justification of the SDQ and WHO-5 wellbeing index.<br>In response, we have removed the unnecessary repetition of these details from this section (**page 5, lines 98-99**) that is already incorporated into the methods section. We believe that this change enhances the clarity and organization of our manuscript.<br>We appreciate your continued attention to detail and your contributions to improving the quality of our work.
b. ***Comment2*** *Page 7: Study objective 3 "to examine the impact of socio-demographic characteristics on mental health issues" should be revised as the cross-sectional nature of the study does not allow for cause-and-effect relationships to be established. Also, the analytical framework used could only examine the association between socio-demographic characteristics and health issues.*	Thank you for your valuable feedback on our study objective 3. We acknowledge your comment that the cross-sectional nature of our study does not allow for the establishment of cause-and-effect relationships.<br>In response to your suggestion, we have revised study objective 3 to read as follows: "To examine the **association** between socio-demographic characteristics and mental health issues." This revised objective more accurately reflects the limitations of our study design and the nature of the analysis we conducted.<br>We believe that this revision addresses your concerns and provides a more accurate representation of our study's scope and limitations. (**Page 7 line 167**)
c. ***Comment3*** *Consider revising the introduction, be succinct and thoughts should follow in a logical sequence.*	Thank you for your valuable feedback on our introduction. We acknowledge your suggestion to revise it for greater succinctness and logical flow. We have carefully reviewed the introduction and made the necessary adjustments removing unnecessary repetition of these details to ensure that our thoughts are presented in a clear and concise manner. (**page 5, lines 98-99**)<br>We believe that these revisions have significantly improved the clarity and coherence of our introduction. Thank you again for your helpful feedback
Reviewer #6 Comments on Results section	
a. ***Comment1*** *It will be helpful to present a table on the socio-demographic characteristics of the participants before presenting other results. This should indicate the frequencies, percentage, and confidence intervals Page 11 - Table 1 should added to supplementary tables or removed from the manuscript.*	Thank you for your valuable feedback regarding the presentation of socio-demographic characteristics in our manuscript. We appreciate your suggestion to provide a more comprehensive overview of our participants' demographics.<br>In response to your comment, we have added a new Table 1 to the main body of the manuscript, presenting the socio-demographic characteristics of our participants, including frequencies and percentages. We believe that this table will enhance the clarity and understanding of our study's findings.

	While we have added Table 1 as you suggested with descriptions (**Page 10-11 line 257-286)**, and we have retained table 1 from previous version**.** We believe that the information contained in Table 2 (current version) remains crucial for a deeper understanding of our research and its implications providing context and details for the readers. ***Previous tables number 1,2,3, and 4 became table 2,3,4 and 5 in the current version.*** We hope that these revisions address your concerns and improve the overall quality of our manuscript. Thank you again for your
b. ***Comment2*** *Page 13- the prevalence estimates as stated in the manuscript does not correspond with results in fig 1. E.g. Prevalence of total difficulties ranged from 14.0%-24.5% not "15.9-25.5%", internalizing 14.9-28.8% not "14.8-28.4%" Please check and revise all the results in the manuscript.*	Thank you for your careful review of our manuscript. We appreciate your attention to detail and your insightful comments. We have carefully reviewed your comment regarding the discrepancies between the prevalence estimates reported in the text and those depicted in Figure 1. You are absolutely correct. There were errors in the text.<br>We have thoroughly checked and corrected all the prevalence estimates in the manuscript to accurately reflect the data presented in Figure 1. The revised manuscript now includes the correct prevalence estimates for total difficulties, internalizing difficulties, and other relevant variables. We believe that these changes have strengthened the clarity and accuracy of our findings. **(Page 14 line 296, 297& 301)**
c. Comment3 Report the results in table 2 in the manuscript. Provide a description beneath the table for "a" as denoted in the table.	Thank you for your valuable feedback. We have carefully considered your comments and have revised the manuscript accordingly. Your suggestion to report the results in Table 2 [current version of the manuscript **table 3** because of addition of a table] was addressed providing more. (**Page 17 line 320-327**). We believe this enhancement will improve the clarity and understanding of our findings."
d. ***Comment4*** *"It was twice higher among adolescents with a self and family members experiencing psychoactive substance use (p0.05)" … state the AOR and CI's.*	Thank you for your careful review of our manuscript. We apologize for any oversight in providing the specific adjusted odds ratios (AORs) and confidence intervals (CIs) for the association between psychoactive substance use and self and family members experiencing such use. We have now included this information in the revised manuscript. Thank you again for your valuable feedback. **Page 18 line 333&334)**
e. ***Comment5*** *Table 3: Use Ref instead of "1" to indicate the reference group in present the results for the logistic regression*	Thank you for your careful review of our manuscript. We appreciate your suggestion to use a more specific reference group indicator in previous Table 3 of table 4 in this current revised version.<br>In the revised manuscript, we have replaced the "1" with "Ref" to clearly indicate the reference group for the logistic regression analysis. (**Page 19**). This change enhances the clarity and interpretability of our results.
f. ***Comment6*** *Present the results for table 4 correlation analysis in the manuscript. The table format is a bit cumbersome. I suggest you review your tables and present them in APA format if not otherwise indicated.*	Thank you for your valuable feedback regarding Table 4 of table 5 in the current version. We apologize for the cumbersome presentation of the correlation analysis results. We have carefully reviewed your comments and have revised Table 4 of table 5 in current version to adhere to APA formatting standards with its brief descriptions. (**Page 21 Table 5)**. We have briefly presented *the results for table 5 correlation analysis. (**Page 22 line 389-398**)*<br>We believe that these changes have significantly improved the clarity and readability of Table 5. Thank you again for your helpful suggestions.

Reviewer #6 Comments on Discussion section *While the authors have provided sufficient references to support the findings of the study, the presentation appears not well structured making it difficult to follow. Consider beginning your discussion by first providing a summary of your key findings based on the objectives for the study. Then you can proceed to discuss them one after the other in an organized and precise manner.*	Thank you for your valuable feedback regarding the presentation of our discussion section. We appreciate your suggestion to restructure our discussion to improve its clarity and coherence.<br>In response to your comment, we have revised the discussion section to begin with a concise summary of our key findings, aligning with the objectives of the study. **(Page 22 line 401-410)** We have then proceeded to discuss each finding in a more organized and precise manner, ensuring that the flow of the discussion is logical and easy to follow.<br>We believe that these changes have significantly enhanced the clarity and readability of our discussion section. Thank you again for your helpful suggestions.
Reviewer #6 Comments on Conclusions section *The conclusion appears a bit elaborate. Please summarize the key objective and key findings and possible recommendation in a precise manner..*	Thank you for your valuable feedback regarding the conclusion of our manuscript. We have carefully considered your comments and have completely revised the conclusion to provide a more concise and focused summary. (**Page 27**)<br>We believe that these changes have significantly improved the clarity and effectiveness of our conclusion. Thank you again for your helpful suggestions.
a. **Comment1** Page 30 paragraph 3 Kindly revise in-text citation. “Anthony F. Jorm and his colleagues” should read Jorm and his colleagues.	Thank you for your careful review of our manuscript. We appreciate your attention to detail and your suggestion regarding the in-text citation on page 30, paragraph 3.<br>We have carefully reviewed your comment and have made the necessary corrections. The in-text citation now reads "Jorm and his colleagues" as you suggested. **(Page 27 Line 589)**<br>This change ensures that our citations are consistent and accurate throughout the manuscript. Thank you again for your valuable feedback.
b. ***Comment2*** *Kindly review the entire manuscript to ensure that all in-text citations are properly cited.*	Thank you for your careful review of our manuscript. We appreciate your suggestion to ensure that all in-text citations are properly cited.<br>We have conducted a thorough review of the entire manuscript to verify the accuracy and consistency of our in-text citations. We have made any necessary corrections to ensure that all references are cited correctly and in accordance with the PLOS ONE style guidelines.<br>We believe that these revisions have strengthened the academic integrity of our work. Thank you again for your valuable feedback.
c. ***Comment3*** *The authors should revise the manuscript to improve the flow and readability of the text.*	Thank you for your valuable feedback regarding the flow and readability of our manuscript. We appreciate your suggestion and have carefully reviewed the entire text to identify areas where improvements could be made.<br>We believe that these revisions have significantly improved the overall readability of our manuscript. Thank you again for your helpful suggestions.
