# Supplementary material for: Mental health issues and the association of mental health literacy among adolescents in urban Ethiopia
Source: PLoS One. 2024 Oct 24;19(10):e0295545. doi: 10.1371/journal.pone.0295545 (PMC11500858; doi:10.1371/journal.pone.0295545)
Supplement: Attachment — Submitted filename: Response to Reviewers.docx [file pone.0295545.s002.docx]

**Response to Reviewers**

**Title: Mental health issues and the association of mental health literacy among adolescents in urban Ethiopia (PONE-D-23-13300)**

**Dear Editor,**

We thank you for your and the reviewers' constructive feedback and the opportunity to revise and resubmit our manuscript. We carefully considered the comments, and the manuscript was revised. We appreciate and thank you very much for your feedback on the suggestion. We followed PLOS ONE's style requirements and moved the ethics statement into the Methods section of the manuscript. We referred to Figure 1 and the tables in the text as part of the main manuscript. We reviewed the reference list to ensure it is complete and correct to check for errors. We have addressed the feedback accordingly.

Three separate papers are submitted to the system: one has a marked-up copy of the manuscript that highlights changes with track changes, an unmarked version of the revised paper without tracked changes, and a rebuttal letter for each point raised by the academic Editor and reviewers. We are confident that the updated version is appropriate for publishing and anticipate hearing from you soon.

With regards,
Hailemariam Mamo Hassen (PhD)
On behalf of the authors.

**Point-by-point response for Reviewers' comments**

Dear reviewers, we are grateful for the feedback and constructive comments helpful for improving the manuscript quality.

**Reviewer #1 Comments**

***Reviewer comment:*** *The article is quite an important work that can be a valuable addition to the literature on mental health, especially in the Ethiopian region. However, the article needs minor revision before being accepted for publication. The comments are addressed below.*

**Author's response**: Thank you. We appreciate your constructive comments and agree on that, and *the comments are addressed below.*

**Reviewer comment:** The precise location of the focus of the study is better mentioned in the abstract to maintain transparent claims and ground of study.

**Author's response:** Thank you very much, dear reviewer. We mentioned the precise location of the focus of the study in the abstract on page 2, line #1, *that the study was conducted in Dire Dawa City, Eastern Ethiopia. The details are also already presented in the main body of the paper.*

***Reviewer comment:*** *The article needs justification for the themes and methods used. For example, it is always better to connect adolescents'' mental health with life span development and justify the argument in such a line of thought.*

***Authors' response:*** Thank you for your comment***.*** We have added more justifications for the choice of themes and methods used to connect adolescents" mental health with life span development. You may kindly refer to the revised manuscript (**Page 4)**

***Reviewer comment:*** *Similarly, more justification is needed in the choice of SDQ and WHO-5 to strengthen the method section.*

***Authors' response:*** Once again, we appreciate your concern. We justified the choice of SDQ, WHO-5, for the measurement of mental health status in the method section. You may kindly refer to the revised manuscript (**Page 5)**

**Reviewer comment:** In the discussion section, the claim "the cut-off score ... for internalizing scores" needs expanded rationalization and justification to make it more relevant to the article's purpose.

***Authors' response:*** Thank you, dear reviewer. We explained the claim and rationalization/justification on "the cut-off score ... for internalizing scores" and other subscales in the discussion section; you may kindly refer to the revised manuscript (**Page 23)**

**Reviewer comment:** Page 8, third paragraph last sentence - "A cross-sectional study across 73 countries..." p.11 conclusion statement "Female adolescents ..." need to strengthen rationalization for what it implies for this study.

***Authors' response:*** Thank you so much for your important concern. *We Strengthened rationalization for what it implies for this study for the sentences on:*

- On the revised manuscript, page 24, fourth paragraph - "A cross-sectional study across 73 countries..."(comment incorporated on page line in the revised version. You may kindly refer to the revised manuscript (**Page 24)**
- On the revised manuscript page 29, second paragraph conclusion statement You may kindly refer to the revised manuscript (**Page 29)**

**Reviewer comment:** More focus is needed on following the prescribed reference style accurately and consistently for in-text citation (P.10) and reference lists (Ref 4,5, 12,16).

**Authors' response:** We appreciate your concern. We checked and revised all the references, including these commented references, as per the PLOS ONE reference style for its accuracy and consistency, including the commented references in-text citation (P.10) and reference list (Ref 4,5, 12,16)

**Reviewer comment:**  Overall, it is a good work to be accepted for publication with these minor revisions.

**Authors' response:** Thank you very much.

**Reviewer #2 Comments:**

**Authors' response to Reviewer #2 Comments:**

Dear reviewer #2, we acknowledge all insightful comments and fully agree with the suggestions. We addressed the comments one by one.

**Reviewer comment:** The introduction provides an exhaustive literature review on mental health issues. When explaining a scenario, start with the global scenario followed by Africa, Ethiopia, and mental health literacy in the study area.

***Authors' response:*** Thank you very much, dear reviewer. We added more explanations, starting with the global scenario followed by Africa, Ethiopia, and mental health literacy in the study area*.* You may kindly refer to the revised manuscript (**Introduction** **Page 3, first paragraph)**

**Reviewer comment:**  What is the government intervention to mitigate the issue? What are the state mechanisms promoting mental health literacy in Ethiopia? The author can discuss these interventions in discussion sections.

***Authors' response:*** Thank you so much for your essential concern. We discussed the government intervention to mitigate the issue, state mechanisms promoting mental health literacy in Ethiopia, and their policy and programme related to mental health for a more comprehensive understanding in the discussion sections with additional references to the national health policy and programme. You may kindly refer to the revised manuscript ( throughout the discussion and mainly in the first paragraph of the discussion part, **Page 22)**

**Reviewer comment:**  The author should discuss their policy and programme related to mental health for a more comprehensive understanding.

***Authors' response:*** We appreciate your crucial concern*. We* discuss the policy and programme related to mental health in Ethiopia throughout the manuscript for a more comprehensive understanding and, most importantly, discussion in the last paragraph of the discussion. (Pages 27-28)

**Reviewer comment:**  The author should highlight the key inference of the present study, how this study enhances the knowledge of mental health literacy among young adolescents in Ethiopia.

***Authors' response:*** Thank you so much. We revised and highlighted the key inference of the study, noting the limitations of generalization in the limitation and conclusion parts (**pages 28&29**).

**Reviewer comment:** Suggest a few major findings, how the demographic and socioeconomic factors are leading in addressing the current mental health literacy issues in Ethiopia.

***Authors' response:*** Thank you, dear reviewer. We revised the manuscript, presenting the significant findings and how the demographic and socioeconomic factors imply addressing the current mental health literacy (Page 29 paragraph 2)

**Reviewer comment:** *Is it applicable across Ethiopia, irrespective of all ethnic groups?*

***Authors' response:*** We appreciate your crucial concern. As it is presented in the limitation section, *It is not applicable to generalize these findings across the country because Ethiopia exhibits multicultural and multiethnic diversity and multiple socio-demographic backgrounds. It could not even be generalized to all adolescents of the study area*. (Page 28, limitation, paragraph 1)
