# Supplementary material for: Mental health issues and the association of mental health literacy among adolescents in urban Ethiopia
Source: PLoS One. 2024 Oct 24;19(10):e0295545. doi: 10.1371/journal.pone.0295545 (PMC11500858; doi:10.1371/journal.pone.0295545)
Supplement: Attachment — Submitted filename: Response to Reviewers.docx [file pone.0295545.s003.docx]

**Response to Reviewers**

**Title: Mental health issues and the association of mental health literacy among adolescents in urban Ethiopia (PONE-D-23-13300)**

**Dear Editor,**

We thank you for your and the reviewers' constructive feedback and the opportunity to revise and resubmit our manuscript. We carefully considered each point raised by the academic editor and reviewers and revised the manuscript. We appreciate and thank you very much for the feedbacks and comments. We followed PLOS ONE's style requirements.

Point raised by the academic editor were regarding whether consent obtained from parents or guardians of the minors, ethics approval letter from two institutions of which one was missing and discrepancy in date difference for secretary and chair person for ethics approval letter obtained from Haramaya University and concern to ensure completeness and correctness of references.

Dear Academic editor, regarding consent from parents or guardians of the minors, written informed consent was obtained from parents of adolescents aged under fifteen years had offered their consent and adolescents of fifteen and above years were factored out following the school counsellors and school principals adequate explanation about their level of maturity and decisional capacity ((page 10) with due reference of the guideline International Ethical Guidelines for Health-related Research Involving Humans: Prepared by the Council for International Organizations of Medical Sciences (CIOMS) in collaboration with the World Health Organization (WHO,2016). However, respective school principals as a duly authorized representative had expressed their consent both orally and signed on consent forms for all participants.

Regarding the ethics approval letters, ethics approval letter from the two institutions (KIIT University and Haramaya University) are merged and attached in this revised submission. The discrepancy of the date of signature from the Chairperson (dated 11/02/2018) and Secretary (dated 11/02/2020) on same document was confirmed as typo while the typist was writing and the letter issued unnoticed. We understand that it requires a correction. However, our application for correction from the Institutional review board of Haramaya University failed because the Institutional review board has restructured and the **Signers** were changed by other individuals. We sincerely request your esteemed editor to understand that this discrepancy is typo while the typist was writing and we kindly request you recognize this issue.

We reviewed the reference lists to ensure it is complete and correct.

Dear editor, we appreciate the comments raised by the reviewers and we accommodate each comment one by one in the manuscript indicated by track change.

Three separate papers are submitted to the system: one has a marked-up copy of the manuscript that highlights changes with track changes, an unmarked version of the revised paper without tracked changes, and a rebuttal letter for each point raised by the academic Editor and reviewers. We are confident that the updated version is appropriate for publishing and anticipate hearing from you soon.

With regards,
Hailemariam Mamo Hassen (PhD), (First and corresponding author)
On behalf of the authors.

**Point-by-point response for Reviewers' comments**

Dear reviewers, we are grateful for the feedback and constructive comments helpful for improving the manuscript quality.

**Reviewer #2 Comments**

Thank you very much, dear reviewers.

Reviewer #2 commented that we authors have addressed all the comments and suggestions stating that mental health literacy is an emerging issue which demand more attention for overall health and well-being.

***Authors' response*** Thank you very much***:***

**Reviewer #3 Comments**

**Author's response**: Thank you. We appreciate your constructive comments and agree on *the comments and addressed accordingly. .*

**Reviewer #3 comment:** Would you please review your claims on the discussion section regarding government actions taken at school level with regard to mental health promotion and prevention as there are no promising activities? These are actually policy promises than actual practices.

***Authors' response:*** Dear Reviewer, thank you so much for your essential concern. We agree that the government actions taken at school level with regard to mental health promotion and prevention are policy promises than actual practices. Accordingly, we discussed these points. *Despite the delayed initiation of the policy and lack of established systems and practices, there has been growing recognition of the importance of adolescent mental health promotion. The Ethiopian government has shown commitment to promoting mental health and addressing mental health issues in schools, for instance, by employing trained school counselors and establishing clubs in schools in secondary schools*. (stated on page 25)

**Reviewer #3 comment**: I think you better consider including the predictor variables in the method part.

**Author's response:** Thank you very much, dear reviewer. We included the predictor variables briefly in the method part (page 8). “The predictor variables mainly focusing on age, grade level, self or any family member experience with psychoactive substance use, parents practicing corporal punishment, perceived worry about family problems, parents education, and job status were collected by using questionnaires.”

**Reviewer #3 comment:** Your sampling strategy should discuss the clusters selected all the stages precisely.

**Author's response:** Thank you. We mentioned the sampling strategy briefly in the method part (page 8). “A combination of multistage (schools, classrooms, then individual students) and systematic and random (using the list of the students in fixed intervals of their roll numbers) was used to select study participants from public and private schools.”

**Reviewer #3 comment:** The statement on page 13 that begins with "These mental health problems were..." does not provide a clear comparison.

**Author's response:** Once again, we appreciate your concern. We have compared and indicated in the figure 1 in the revised manuscript, page 14. We stated that **“**These mental health problems were more prevalent for female and male adolescents compared to other age groups in extent of percentage as explicitly visualized in Figure 1 across the three age groups. Prevalence of depression ranged from 18.0%-25.5%, reportedly higher for female adolescents aged 14-16 years ( page 14).

**Reviewer #3 comment:** Would you please mention any bases for your age categorization and its subsequent implication for mental health promotion and prevention?

**Author's response:** Thank you very much, dear reviewer. We already mentioned the bases for your age categorization and its subsequent implication on page 11 of the last 4 lines #6-9; “*The age range for adolescents and grouping was based on UNICEF adolescents' age categorization as early, middle, and late adolescence age groups, which is helpful to target mental health promotion and prevention efforts during these stages to address their respective mental health demands*.”
